# Phylogeny, Evolution and Classification of Gall Wasps: The Plot Thickens

**DOI:** 10.1371/journal.pone.0123301

**Published:** 2015-05-20

**Authors:** Fredrik Ronquist, José-Luis Nieves-Aldrey, Matthew L. Buffington, Zhiwei Liu, Johan Liljeblad, Johan A. A. Nylander

**Affiliations:** 1 Department of Bioinformatics and Genetics, Swedish Museum of Natural History, Stockholm, Sweden; 2 Departamento de Biodiversidad y Biología Evolutiva, Museo Nacional de Ciencias Naturales (CSIC), Madrid, Spain; 3 Systematic Entomology Lab, USDA, c/o Smithsonian Institution, Washington DC, United States of America; 4 Department of Biological Sciences, Eastern Illinois University, Charleston, IL, United States of America; 5 Swedish Species Information Centre, Swedish University of Agricultural Sciences, Uppsala, Sweden; 6 Bioinformatics Infrastructure for Life Sciences, Linköping University, Linköping, Sweden; Institut National de la Recherche Agronomique (INRA), FRANCE

## Abstract

Gall wasps (Cynipidae) represent the most spectacular radiation of gall-inducing insects. In addition to true gall formers, gall wasps also include phytophagous inquilines, which live inside the galls induced by gall wasps or other insects. Here we present the first comprehensive molecular and total-evidence analyses of higher-level gall wasp relationships. We studied more than 100 taxa representing a rich selection of outgroups and the majority of described cynipid genera outside the diverse oak gall wasps (Cynipini), which were more sparsely sampled. About 5 kb of nucleotide data from one mitochondrial (COI) and four nuclear (28S, LWRh, EF1alpha F1, and EF1alpha F2) markers were analyzed separately and in combination with morphological and life-history data. According to previous morphology-based studies, gall wasps evolved in the Northern Hemisphere and were initially herb gallers. Inquilines originated once from gall inducers that lost the ability to initiate galls. Our results, albeit not conclusive, suggest a different scenario. The first gall wasps were more likely associated with woody host plants, and there must have been multiple origins of gall inducers, inquilines or both. One possibility is that gall inducers arose independently from inquilines in several lineages. Except for these surprising results, our analyses are largely consistent with previous studies. They confirm that gall wasps are conservative in their host-plant preferences, and that herb-galling lineages have radiated repeatedly onto the same set of unrelated host plants. We propose a revised classification of the family into twelve tribes, which are strongly supported as monophyletic across independent datasets. Four are new: Aulacideini, Phanacidini, Diastrophini and Ceroptresini. We present a key to the tribes and discuss their morphological and biological diversity. Until the relationships among the tribes are resolved, the origin and early evolution of gall wasps will remain elusive.

## Introduction

Galls represent one of the most remarkable products of biological evolution. They are structures formed by plants entirely for the benefit of another organism, the gall inducer. Not only do the galls provide shelter and nutrition for the gall inducer, they can also protect the gall inducer from its natural enemies by sophisticated mechanical devices or by toxic tissue layers. In the late 19^th^ century, many biologists believed that the formation of the gall was under control of the plant. If so, it was recognized, galls posed a major threat to Darwin’s theory of natural selection because they apparently were of no selective advantage to the plant [1–4]. Now it is commonly believed that the formation of the gall is essentially controlled by the foreign organism, although the exact mechanism is unknown in most cases.

Next to gall midges, gall wasps (Hymenoptera: Cynipidae) constitute the largest radiation of gall-inducing organisms with roughly 1,400 described species. They occur on all continents, except for the Antarctic, but the largest number of species are found in the temperate areas of the Northern Hemisphere. The gall is induced after the female wasp has laid her eggs in the plant tissue. The gall serves as protection but also provides nourishment for the developing larva inside. Cynipid galls range in complexity from cryptic chambers inside herb stems, to distinct swellings on various plant organs or complex structures that bear no resemblance to the attacked plant organ. Each species of gall wasp typically attacks a single host-plant species, or a set of very closely related plant species, and induces a particular type of gall on a particular plant organ. The most important hosts are trees or bushes: usually oaks (*Quercus*) and other trees of the family Fagaceae (*Lithocarpus*, *Chrysolepis*, *Castanopsis* and *Castanea*) or roses (*Rosa*). However, there are also a significant number of herb gallers, which favor host plants in the families Asteraceae, Lamiaceae, Rosaceae and Papaveraceae. Some gall wasps do not induce galls. Instead, their larvae develop inside the galls of other species, typically those of other gall wasps. These forms are termed *inquilines*. For reasons that are unclear, they exclusively attack galls on woody plants.

The current classification of the Cynipidae places all extant forms in a single subfamily, with the majority of species falling into one of four tribes: oak gallers (Cynipini), herb gallers (Aylacini sensu lato), rose gallers (Diplolepidini), and inquilines (Synergini sensu lato) (Table 1). In addition to herb gallers, the tribe Aylacini sensu lato also includes *Diastrophus*, the members of which attack both rosaceous herbs (mainly *Potentilla*) and bushes of *Rubus*. Looking beyond the four big tribes, the Cynipidae have also traditionally included two minor tribes: the Pediaspidini (maple gallers), and the Eschatocerini (gallers of *Acacia* and *Prosopis* in the Fabacae) (Table 1). In recent years, two additional tribes have been described based on morphologically divergent forms from the Southern Hemisphere: Qwaqwaiini, including a single gall inducer on *Scolopia* (Salicaceae) in South Africa [5] and Paraulacini, including two genera (*Paraulax* and *Cecinothofagus*) of inquilines (or possibly parasitoids) in chalcidoid galls on *Nothofagus* (Nothofagaceae) in southern South America [6].

**Table 1:** Overview of the taxonomy, diversity and biology of Cynipoidea.

Family	Diversity^1^	Distribution	Biology
Austrocynipidae	1/1	Australia	Parasitoids of Lepidoptera in cones
Ibaliidae	3/22	Holarctic	Parasitoids of Hymenoptera in wood
Liopteridae	10/170	Widespread, mainly tropical	Parasitoids of Coleoptera in wood
Figitidae	140/1569	Cosmopolitan	Parasitoids of Diptera, Hymenoptera and Neuroptera
Parnipinae	1/1	Mediterranean	Parasitoid of gall-inducing Aylacini (*Barbotinia*)
Euceroptrinae	1/4	Nearctic	Parasitoids (or possibly inquilines) of gall-inducing Cynipini
Mikeiinae	1/5	Australia	Parasitoids (or possibly inquilines) of gall-inducing chalcidoids
Plectocynipinae	2/6	South America	Parasitoids of gall-inducing chalcidoids
Thrasorinae	4/20	Widespread	Parasitoids of gall-inducing chalcidoids
Aspicerinae	10/126	Cosmopolitan	Parasitoids of Diptera in aphid communities
Figitinae	14/144	Cosmopolitan	Parasitoids of Diptera
Anacharitinae	8/83	Widespread, mainly Holarctic	Parasitoids of Neuroptera in aphid communities
Charipinae	8/168	Cosmopolitan	Parasitoids of Hymenoptera in aphid communities
Eucoilinae	83/987	Cosmopolitan	Parasitoids of Diptera
Emargininae	5/15	Cosmopolitan	Unknown
Pycnostigminae	3/10	South Africa & Mediterranean	Unknown
Cynipidae	72/1439	Mainly Holarctic	Phytophagous gall inducers or inquilines
Synergini s. lat.	8/202	Mainly Holarctic	Inquilines in galls of other insects, usually other cynipids
Aylacini s. lat.	22/170	Holarctic	Gallers on eudicot herbs, one genus also on *Smilax* vines and *Rubus* bushes
Diplolepidini	2/55	Holarctic	Gallers on *Rosa*
Eschatocerini	1/3	South America	Gallers on *Acacia* and *Prosopis* (Fabaceae)
Pediaspidini	2/2	Palearctic	Gallers on *Acer*
Paraulacini	2/6	South America	Inquilines or parasitoids in chalcidoid galls on *Nothofagus* (Nothofagaceae)
Qwaqwaiini	1/1	South Africa	Gallers on *Scolopia* (Salicaceae)
Cynipini	34/1000	Mainly Holarctic	Gallers on Fagaceae, mostly on *Quercus*

All parasitoids attack the larval stage of the host.

^1^Number of genera / species.

Gall wasps belong to the parasitic-wasp superfamily Cynipoidea, the other members of which are classified into four families (Austrocynipidae, Ibaliidae, Liopteridae, and Figitidae). As far as is known, the parasitic forms are all early-internal late-external parasitoids of other endopterygote insect larvae (Table 1). Whereas the Austrocynipidae, Ibaliidae, and Liopteridae all attack larvae tunneling in wood or in cones (based on circumstantial evidence in the case of Austrocynipidae and Liopteridae), the Figitidae attack hosts in various microhabitats. The Figitidae are thought to be the sister group of Cynipidae [7], and several figitid lineages are associated with galls. These forms include the Parnipinae [8], Euceroptrinae [9], Thrasorinae [10], Mikeiinae [11] and Plectocynipinae [12–13]. The biology of most of these forms is poorly known but they are presumably all parasitoids of gall-inhabiting larvae, which is likely to be the ancestral life history of both families [14, 15].

The origin and early evolution of gall wasps have fascinated biologists for a long time. Alfred Kinsey, probably more well-known for his research on human sexual behavior, presented the first hypothesis of higher gall-wasp relationships based on morphological and biological features [16]. Kinsey considered oak and rose gallers to be derived from herb-galling lineages and thought that the first cynipids were “plant-tissue inhabiting, not gall-making insects” ([16], p. 400) similar to the extant gall wasps that develop in cryptic gall chambers inside stems of various herbs in the family Asteraceae. The subsequent evolution of cynipid galls supposedly involved irreversible trends from structurally simple to complex galls, from multi- to single-chambered galls, and from integral to detachable galls [16]. Other early workers similarly speculated on the first cynipid galls being multi-chambered stem swellings, and subsequent evolution leading to an increase in gall complexity [17–18]. Malyshev [19], on the other hand, argued that the first cynipids were more likely to have been associated with oaks than with herbs belonging to Asteraceae, since the latter represent a more recent radiation, and proposed that the gallers evolved from seed rather than stem feeders, the first galls being induced in reproductive buds or developing seeds. However, most subsequent authors have accepted Kinsey’s rather than Malyshev’s scenario as being more probable. For instance, Roskam [20] argued that an ancestral association with the Asteraceae is possible since the gall wasps constitute a recent radiation, consistent with Kinsey’s view [21] that cynipids are not much older than the Oligocene.

The origin of the inquilines has also been debated in the literature. Early systematists recognized the similarities among inquilines and grouped them together [22–23], indicating that they might have had a single origin. Others have suggested that the inquilines are polyphyletic, with each inquiline being more closely related to its particular host gall inducer [24–25]. A third possibility that has been raised is that the inquilines represent ancient forms that never evolved the ability of inducing galls on their own [19, 26].

**Fig 1:**
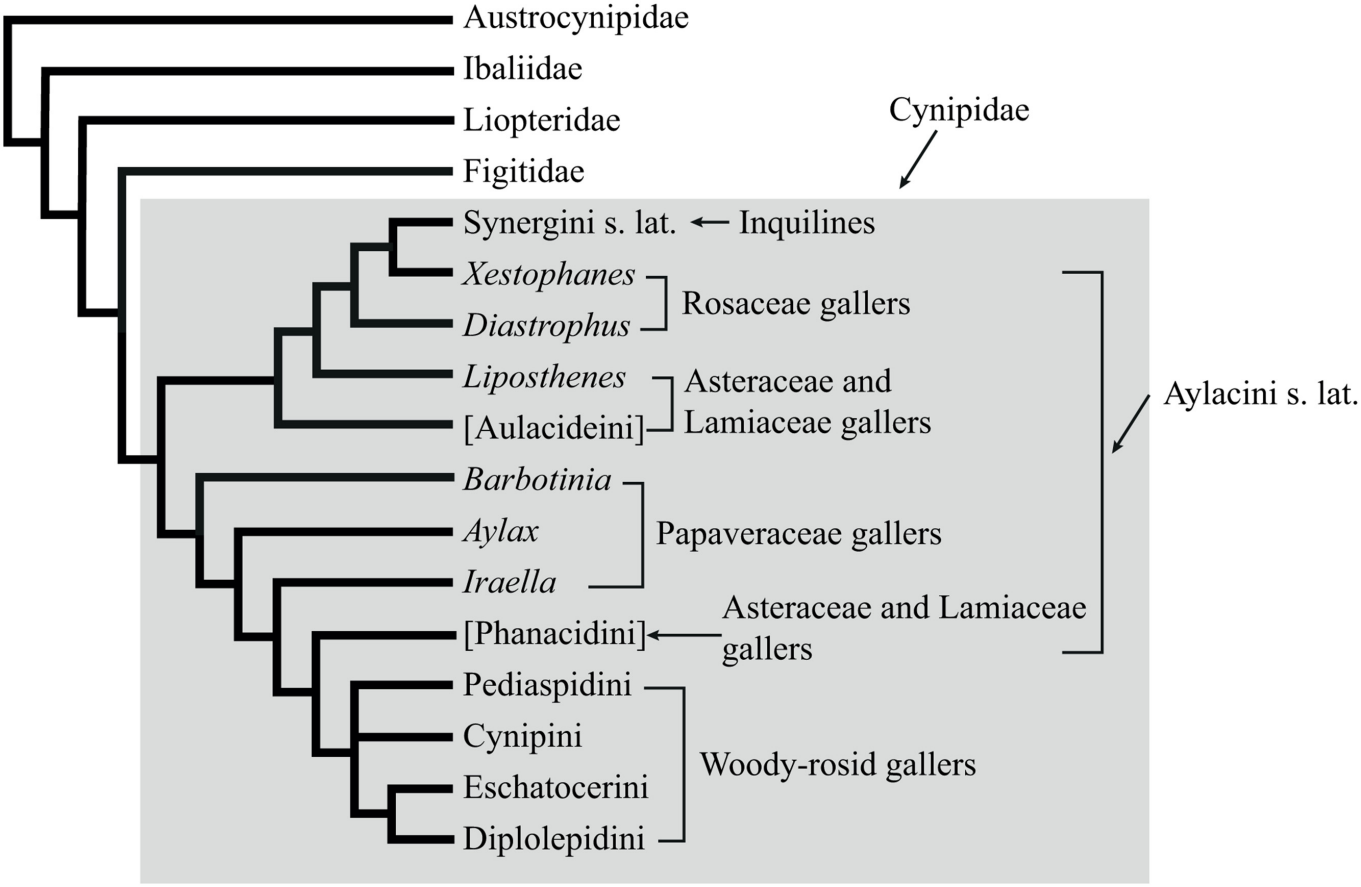
Relationships among gall wasps and their insect-parasitic relatives based on previous analyses of adult morphology [7, 14, 27, 28, 34]. Tribal concepts introduced in the current paper are placed in square brackets. Note that the genus Liposthenes was placed outside the Aulacideini in these analyses, but subsequent analyses have shown that it should be placed inside it.

Analyses based on extensive datasets of morphological characters of adults [7, 14, 27–29] have lent strong support to the idea that the inquilines had a single origin and are most closely related to the Rosaceae gallers in the tribe Aylacini, *Xestophanes* and *Diastrophus*. They have also suggested that the Aylacini are paraphyletic and form the basal lineages in the Cynipidae, whereas the remaining cynipid tribes (Cynipini, Diplolepidini, Eschatocerini, and Pediaspidini) are each monophyletic and together form a lineage termed the *woody-rosid gallers* (WRG) because they induce galls on woody members of the eudicot subclade Rosidae [28, 30] (Fig 1). Unfortunately, these studies did not include representatives from the recently described tribes Paraulacini and Qwaqwaiini.

Mapping life-history traits onto this phylogeny implies that gall wasps originated in the Palearctic, and that they initially induced single-chambered, distinct swellings in the reproductive parts of Papaveraceae or possibly Lamiaceae [29]. Similar studies of the insect-parasitic relatives of gall wasps suggest that this ancestor evolved from a parasitoid of a hymenopteran gall inhabitant [14, 15, 31]. Mapping studies also indicate that the host-plant preferences of gall wasps are extremely conservative but that there have nevertheless been some remarkable cases of independent radiation onto the same set of distantly related herbaceous host plants in separate cynipid lineages [29].

Nylander et al. [32] presented the first molecular analyses of higher-level cynipid relationships based on four genes (about 3.0 kb of COI, EF1aF1, LWRh, and 28S rDNA data) sequenced for 32 taxa. These results challenged the morphology-based conclusions on gall-wasp evolution in several important respects. Most surprisingly, the molecular data indicated that the WRG and the inquilines are both polyphyletic assemblages of basal cynipid lineages. If this is true, then the first cynipids might have been associated with woody hosts rather than herbs, and the inquilines might have been ancestral to the gall inducers, as suggested by Malyshev [19], rather than derived from them. The small number of taxa and sequences, however, cast some doubt on the results from this pioneering study.

Here, we analyze a considerably extended molecular dataset for cynipids, both alone and combined with partly new morphological and life history data, to shed further light on the evolution of the group. The analysis includes 103 taxa in total, for 97 of which we were able to obtain molecular data. The studied taxa include representatives of all eight cynipid tribes, including the recently described Paraulacini and Qwaqwaiini, and at least one exemplar of virtually all described genera outside of the Cynipini. Outgroup representatives include, among others, three of the four gall-associated figitid subfamilies. The analyses are based on five molecular markers (about 5 kb of EF1aF1, EF1aF2, LWRh, COI and 28S data), 228 morphological characters, and 11 life-history traits. Based on the results of phylogenetic analyses of these data, we reanalyze the evolution of gall wasps and propose a new tribal classification of the family, including four new tribes and significantly revised circumscriptions of two tribes.

## Materials and Methods

### Data

We assembled DNA data for a total of 97 exemplars representing all families of cynipoids except for the Australian endemic Austrocynipidae (only known from five specimens) (S1 Table). The vast majority of the described cynipid genera were included except for the tribe Cynipini (the oak gallers), which was represented by only seven of the 34 recognized genera since they are widely considered monophyletic [14, 16, 25, 27–29, 33–38]. Among the insect-parasitic forms we had representatives from eight of twelve figitid subfamilies (Thrasorinae, Mikeiinae, Emargininae and Pycnostigminae missing), two of four liopterid subfamilies (Liopterinae and Oberthuerellinae missing), and one of three ibaliid genera.

We sequenced parts of five genes (S1 Table). The majority of taxa were sequenced for the mitochondrial gene cytochrome oxidase *c* subunit I (COI, 1,078 bp), the nuclear protein-coding gene elongation factor 1 alpha, F1 copy (EF1aF1, 367 bp), and the nuclear ribosomal gene 28S (1,246 bp). This was complemented for some taxa by the nuclear protein-coding genes elongation factor alpha, F2 copy (EF1aF2, 1,101 bp; 23 taxa), long-wavelength rhodopsin (LWRh, 481 bp; 22 taxa), and longer sequences of EF1aF1 (1,069 bp; 17 taxa). Details of the DNA amplification protocols and primers appear elsewhere [39–41]. The protein-coding genes were easily aligned by eye (introns were removed). The ribosomal (28S) sequences were aligned using MUSCLE [42], with default settings.

Morphological data were assembled from the literature [7, 14, 28, 43, 44] with some modification, correction and additional coding of new taxa (S1 Appendix). Terminology follows the Hymenoptera Anatomy Ontology [45]. Morphological data were available for 43 of the 76 sequenced cynipids, 19 of the 21 sequenced outgroups, and six cynipids that were not sequenced. Taxa with alternating generations were coded as polymorphic when we had data from both generations and they differed in their morphological traits.

Eleven biological (life-history) characters were coded from information in the literature [14, 28, 29] complemented with data for the taxa added in this study (S1 Appendix). Taxa with alternating generations were coded as polymorphic when the generations differed in their biological traits. The combined molecular and morphological dataset is available from TreeBase, accession number 15832 (http://purl.org/phylo/treebase/phylows/study/TB2:S15832) and as S1–S3 Datasets.

### Phylogenetic analysis

Phylogenetic analysis was performed using MrBayes versions 3.2.1 and 3.2.2 [46]. For the morphological and biological data, we used the Mk model [47], extended to deal with multi-state ordered and unordered characters, with gamma-shaped rate variation across characters. DNA data were modeled using four-by-four nucleotide models, where we estimated stationary state frequencies and integrated across all possible ways of grouping or ungrouping exchangeability rate parameters [48]. Rates across sites were modeled using the invariable sites plus gamma model. Each protein-coding gene was divided into two partitions: first and second codon positions versus third codon positions. The base rate and all substitution model parameters were uncoupled across the eleven data partitions (morphology, biology, 28S, and two partitions each for COI, EF1aF1, EF1aF2, and LWRh). Default priors were used for all parameters.

In addition to analyzing the complete data set, we also ran separate analyses for each of the genes, and for the morphological and biological data partitions. We also ran analyses for the morphological and biological data combined, for the molecular data combined, and for the morphological and molecular data combined (without the biological data). The molecular data were partly incomplete, and to examine the effect of the missing data we ran the complete data set and the combined molecular data without EF1aF2, LWRh, and the longer EF1aF1 sequences (the markers available for the smallest number of taxa). To examine alternative topological hypotheses, we ran four analyses under topological constraints: (1) Figitidae forced to be monophyletic; (2) Core Figitidae (Figitidae excluding the gall-associated taxa *Parnips*, *Plectocynips*, and *Euceroptres*) forced to be monophyletic; (3) Woody rosid gallers (Cynipini, Pediaspidini, Diplolepidini, Eschatocerini, and Qwaqwaiini) forced to be monophyletic, with Paraulacini allowed to float (to be inside or outside); and (4) Inquilines forced to be monophyletic, with Paraulacini allowed to float.

To evaluate diagnostic features of the tribes we propose here, and to evaluate different scenarios for the early evolution of the group, we ran a separate analysis where we constrained Cynipidae and all tribes to be monophyletic, and then inferred the ancestral states for all the constrained nodes using the “report ancstates = yes” option in MrBayes [46]. We also constructed two indices to indicate the distinctness and uniqueness of the reconstructed ancestral traits for the twelve cynipid tribes. The *distinctness index* was calculated as the posterior probability (PP) of an ancestral trait of a tribe from which was subtracted the mean PP of the same trait among the other tribes and in the most recent common ancestor of the Cynipidae. The *uniqueness index* was calculated as the PP of an ancestral trait of a tribe from which was subtracted the maximum PP of the same trait among the other tribes and in the Cynipidae ancestor. In both cases, the PP of a trait (a particular state of a character) for a node was estimated as the mean of the sampled PP values for that state and node. Both indices have a maximum value of 1.0. The distinctness index will be high for unusual traits (potentially shared with one or a few other nodes considered), whereas the uniqueness index will be high only for unique traits (among the nodes considered).

Each analysis involved four independent runs, each using four Metropolis-coupled chains, under default settings. The runs were stopped after 100 M generations. The chains were sampled once every 1,000 generations and the initial 25% of samples were discarded as burn in. All analyses converged to an average standard deviation of split frequencies below 0.05, usually below 0.01, and all branch lengths and substitution model parameters had potential scale reduction factors less than 1.01. The support for individual clades is specified in the text as the mean of the estimated PP values across the four independent runs. In the figures, we also give the standard deviation across runs if it was larger than or equal to 0.01.

Topological hypotheses were compared using posterior model probabilities, which provide a more appropriate way of assessing monophyly hypotheses than simple Bayes factor tests [49]. Posterior model probabilities can be estimated more accurately than Bayes factors, to the required precision, and they are easy to interpret. For instance, a posterior probability smaller than 0.001 for a topology hypothesis simply means that the probability that the hypothesis is correct, given the model and the prior, is less than 0.1%. This can be considered very strong evidence against the hypothesis, comparable to the 0.001 confidence level of traditional statistical tests. The MrBayes command blocks for all analyses are provided as S1 Commands.

### Nomenclatural acts

In the revised tribal classification of the Cynipidae presented in this paper based on the phylogenetic results, four new tribes are proposed. The electronic edition of this article conforms to the requirements of the amended International Code of Zoological Nomenclature, and hence the new names contained herein are available under that Code from the electronic edition of this article. This published work and the nomenclatural acts it contains have been registered in ZooBank, the online registration system for the ICZN. The ZooBank LSIDs (Life Science Identifiers) can be resolved and the associated information viewed through any standard web browser by appending the LSID to the prefix "http://zoobank.org/". The LSID for this publication is: urn:lsid:zoobank.org:pub:5D133F08-EDA6-464C-ADDC-16C936297C44. The electronic edition of this work was published in a journal with an ISSN, and has been archived and is available from the digital repositories PubMed Central, LOCKSS and Digital CSIC.

## Results

### Combined morphological, biological and molecular data

**Fig 2:**
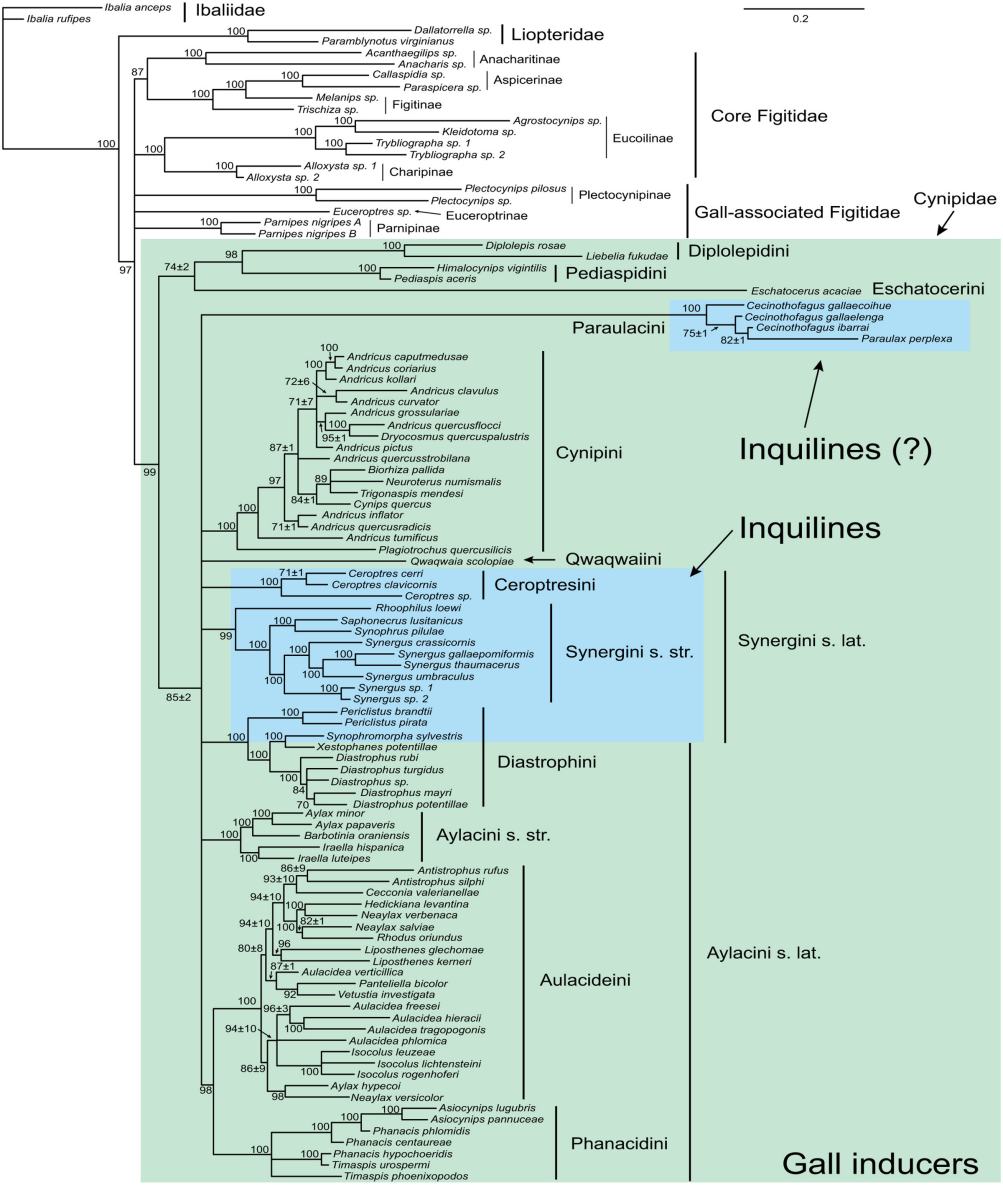
Results from analysis of the combined molecular, morphological and life-history data. Numbers on branches indicate estimated posterior clade probabilities (in % units) ± Monte Carlo error (error smaller than 1% not shown) across four separate analyses. Only groups with posterior probability above 70% shown.

When rooted on ibaliids, the analysis of the combined data shows the expected sister-group relationship between Liopteridae and microcynipoids (Figitidae + Cynipidae) (Fig 2). There is strong evidence for the monophyly of Cynipidae (PP 0.99) but not for the monophyly of the Figitidae. Relationships among figitid subfamilies are largely unresolved, with two notable exceptions. First, the Charipinae and Eucoilinae form sister groups (PP 1.00 for the clade consisting of both subfamilies). Second, the Aspicerinae and Figitinae together constitute a monophyletic group (PP 1.00), with the Aspicerinae nested within a paraphyletic Figitinae.

Within the cynipids, a number of higher clades are strongly supported. These include four currently recognized tribes: the oak gallers (Cynipini; PP 1.00), the rose gallers (Diplolepidini; 1.00), the maple gallers (Pediaspidini; PP 1.00), and the inhabitants of galls on southern beeches, *Nothofagus* (Paraulacini; PP 1.00). However, neither the Aylacini (the herb gallers) nor the Synergini (the inquilines) appear as monophyletic as circumscribed currently (sensu lato). Instead, we found strong support (PP 1.00) for a clade including some herb gallers (*Xestophanes* and *Diastrophus*) and some inquilines (*Synophromorpha* and *Periclistus*), all of which are associated with galls on Rosaceae. This clade will be named Diastrophini below. The remaining inquilines fall into two distinct clades: (1) the *Synergus* complex including *Synergus*, *Saphonecrus*, *Synophrus* and *Rhoophilus* (PP 0.99) (Synergini sensu stricto); and (2) the genus *Ceroptres* (PP 1.00) (proposed below as Ceroptresini). The remaining herb gallers fall into three major clades: (1) a small clade of Papaveraceae gallers, including *Barbotinia*, *Iraella* and *Aylax* (Aylacini sensu stricto; PP 1.00) (2) a medium-size clade of stem gallers, mostly on Asteraceae, including *Timaspis*, *Phanacis*, and *Asiocynips* (named Phanacidini below; PP 1.00); and (3) a large clade mostly including gallers on Lamiaceae and Asteraceae (*Liposthenes*, *Aulacidea*, *Isocolus*, and related genera) (named Aulacideini below; PP 1.00).

Relationships among these clades remain uncertain with two exceptions. There is fairly strong support (PP 0.98) for a sister-group relationship between the two main herb-galling lineages, Phanacidini and Aulacideini. Second, it appears that the Diplolepidini and Pediaspidini form a monophyletic clade (PP 0.98). There is some indication that the tribes other than Diplolepidini, Pediaspidini and Eschatocerini form a clade, but the support value is relatively low for a Bayesian phylogenetic analysis (PP 0.85).

Within the Diastrophini, it is worth noting that the genus *Periclistus*, inquilines in *Diplolepis* galls on roses, appears to be the sister group of the remaining taxa (the latter having PP 1.00), and that *Synophromorpha* (inquilines in *Diastrophus* galls on *Rubus* bushes) and *Xestophanes* (gall inducers on *Potentilla*) appear to form a clade (PP 1.00). Within the Cynipini, the genus *Plagiotrochus* forms the sister-group of the remainder of the tribe (the latter having PP 1.00).

Relationships among the Aulacideini are only partly resolved by our analysis, but several interesting patterns emerge nevertheless. In particular, it appears that the tribe falls into three subgroups that can be characterized by host plant preferences. The smallest group includes gallers of *Hypecoum* and *Fumaria* (subfamily Fumarioideae of Papaveraceae) (*Aylax hypecoi* and *Neaylax versicolor*), two species that form a strongly supported clade (PP 0.98). The second is a large clade of Lamiaceae gallers, somewhat less strongly supported (PP 0.80), including *Aulacidea verticillica*, *Panteliella*, *Vetustia*, *Liposthenes*, *Neaylax* (other than *N*. *versicolor*), *Rhodus* and *Hedickiana*. Interestingly, *Cecconia valerianellae*, which is unique among cynipids in galling Valerianacae (*Valerianella*), is nested deep inside this clade. The third and final group is a strongly supported (PP 0.94) clade of Asteraceae gallers, including the species-rich genera *Aulacidea* (except *A*. *verticillica*) and *Isocolus*. It also includes a single species galling Lamiaceae (*Aulacidea phlomica*).

### Data partitions

**Fig 3:**
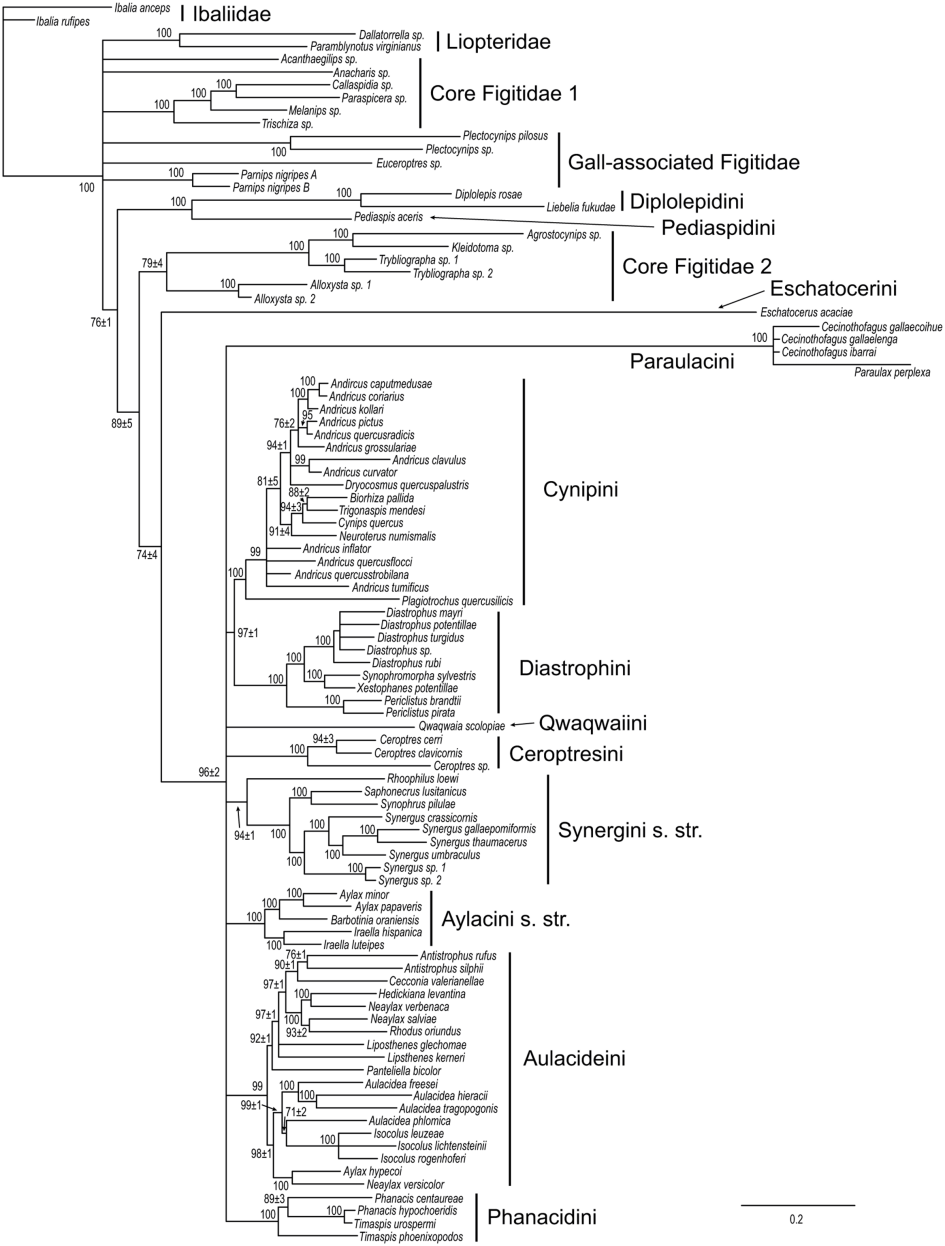
Results from analysis of the combined molecular data. Numbers on branches indicate posterior clade probabilities (in % units) ± Monte Carlo error (error smaller than 1% not shown)across four separate analyses. Only groups with posterior probability above 70% shown.

The combined molecular tree (Fig 3) was almost identical to the total-evidence tree (including also morphology and life-history traits) with two exceptions. First, it did not support a sister-group relationship between Aulacideini and Phanacidini but grouped Diastrophini with Cynipini (PP 0.97) instead. More surprisingly, it did not support cynipid monophyly because Charipinae + Eucoilinae (part of the core Figitidae) were placed inside the Cynipidae, among the basal lineages. Thus, the support for cynipid monophyly in the total-evidence analysis is partly due to signal in the morphological and life-history characters. The major cynipid clades and the relationships within them were the same in the molecular tree as in the total-evidence tree (Figs 2 and 3).

Individual gene trees (S1–S5 Figs) were much less resolved but nevertheless agreed well with the combined molecular tree and with the total-evidence tree. Nearly all the signal in the nuclear protein-coding genes originated from variation in the third codon position. In the mitochondrial COI, however, there was signal also in first and second codon positions. The signal in the first and second codon positions of COI was less resolved but otherwise agreed well with the signal from the analyses of the complete sequences, including the third codon position.

Of the twelve major cynipid clades recognized as tribes in this paper, eight were strongly supported as monophyletic in two or more independent gene data partitions (Table 2). The remaining four tribes—Pediaspidini, Eschatocerini, Qwaqwaiini, and Ceroptresini—were represented by single taxa in the molecular analyses, and their monophyly could therefore not be tested. The only exception was the Ceroptresini, which were represented by two taxa in the 28S analysis. However, this analysis placed the Ceroptresini exemplars separately in a large basal polytomy, among many other cynipid and figitid taxa, so it was uninformative with respect to the monophyly of Ceroptresini.

**Table 2:** Support for the cynipid tribes recognized in this paper across data partitions.

	Data partition					
Clade	Morph-bio^1^		28S	COI^2^	EF1aF1	EF1aF2	LWRh
Cynipini	0.90		<0.50	0.99 (0.91)	0.99^3^	–	1.00^4^
Diplolepidini	<0.50		0.93	1.00 (1.00)	0.99	1.00	single
Pediaspidini	1.00		single	single	single	single	single
Eschatocerini	single		single	single	single	single	single
Qwaqwaiini	single		single	single	–	–	–
Paraulacini	1.00		1.00	1.00 (1.00)	single	–	–
Aylacini s. str.	<0.50		1.00	0.84 (0.62)	1.00	single	0.69
Aulacideini	<0.50		0.70	<0.50 (<0.50)	0.99	1.00	1.00
Phanacidini	<0.50		0.98	1.00 (0.99)	0.98	–	single
Diastrophini	<0.50		0.63	0.99 (0.83)	1.00	1.00	single
Synergini s. str.	0.88^5^		1.00^6^	0.98 (0.60)	1.00^6^	single	single
Ceroptresini	single		<0.50	1.00 (0.98)	single	single	single

^1^Morphology and life-history traits.

^2^Values in parentheses refer to analysis excluding third codon position.

^3^Including *Eschatocerus* but excluding *Plagiotrochus*.

^4^Excluding *Plagiotrochus*.

^5^Including *Plectocynips*, *Qwaqwaia* and Paraulacini.

^6^Excluding *Rhoophilus*.

**Fig 4:**
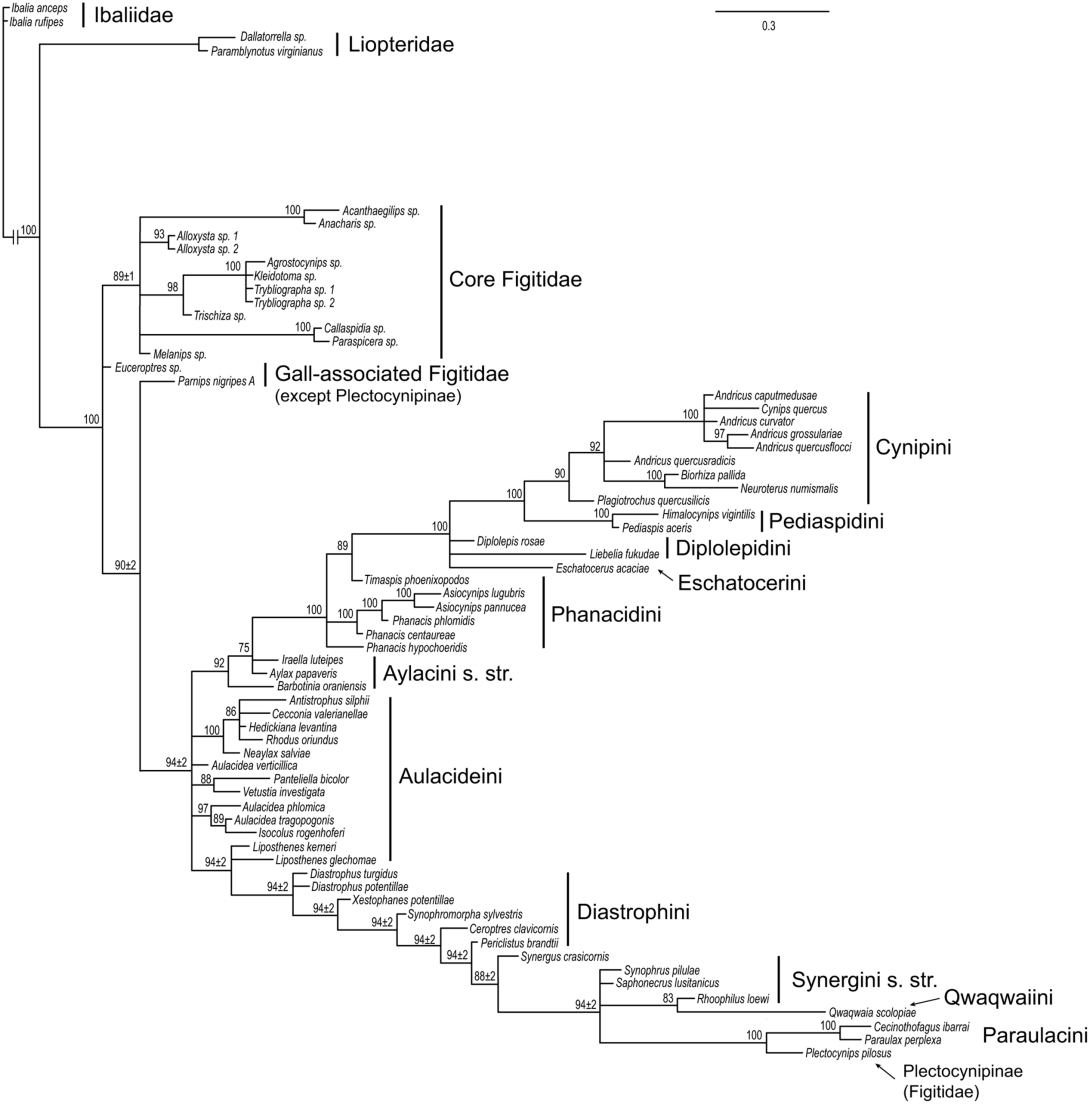
Results from analysis of the morphological and biological data. Numbers on branches indicate posterior clade probabilities (in % units) ± Monte Carlo error (error smaller than 1% not shown) across four separate analyses. Only groups with posterior probability above 70% shown.

The tree based on morphological and life-history traits (Fig 4) was consistent with the molecular tree in providing support for the monophyly of Cynipini (Table 2), but differed in many other respects. Perhaps the most striking difference is that it placed Synergini sensu lato within a paraphyletic Diastrophini. The Synergini clade also included *Plectocynips* (one of the gall-associated figitids), Qwaqwaiini, and Paraulacini, in addition to the taxa placed there traditionally (those belonging to Synergini sensu lato). *Plectocynips* (like other outgroups) was scored only for select morphological characters in our analysis, which could have contributed to uncertainty or biases in its placement. However, it does resemble the inquilines in some morphological features, particularly the Paraulacini. Qwaqwaiini and Paraulacini display somewhat more extensive similarities to the inquilines, but their placement inside the Synergini sensu lato was nevertheless surprising.

Other differences between the morphology + biology tree and the molecular tree include the support for the monophyly of the woody rosid gallers (excluding Qwaqwaiini) in the former, a clade that did not appear in the molecular tree. Also, the Aylacini sensu stricto and Phanacidini both emerged as paraphyletic grades in the lineage leading to woody rosid gallers (Cynipini, Diplolepidini, Eschatocerini, and Pediaspidini), while the Aulacideini appeared as an unresolved assemblage of basal cynipid lineages, with *Liposthenes* grouping fairly strongly (PP 0.94) with Diastrophini and the groups nested within that clade.

Separate analysis of the life-history data (11 characters) failed to provide any phylogenetic resolution at all (no groups with PP ≥ 0.75). The morphological data (228 characters) alone, however, generated a tree that was similar to the one from the combined morphology + biology analysis, but less well resolved (S6 Fig). In particular, the nesting of Plectocynipinae, Paraulacini, Qwaqwaiini and Synergini sensu stricto inside Diastrophini was not supported, demonstrating that this signal was due to the interaction between morphological and biological data.

Finally, we examined the effects of deleting the life-history traits from the total-evidence analysis (S7 Fig), and also removing the assumption of state ordering for some morphological characters (S8 Fig). In both cases, the results were almost identical to the combined analysis (Fig 2), although the support decreased slightly for a few clades.

### Missing data and constraints

The results from the combined-data and molecular-data analyses with incompletely coded gene regions removed indicated no problems with missing data. In the combined-data analysis (S9 Fig), the support for Aulacideini + Phanacidini forming a clade disappeared, relationships among the Aulacideini were more poorly resolved, and the PP for cynipid monophyly dropped from 0.99 to 0.85. Otherwise, the results were very similar to the analysis with all gene regions included. Results from the analysis of the molecular data with incompletely coded gene regions (S10 Fig) were also similar to those of the complete molecular data, except that it was *Euceroptres* (one of the gall-associated figitids) that ended up inside the Cynipidae instead of the Eucoilinae + Charipinae.

Because there was weak support in our analyses for relationships within the Figitidae, the supposed sister-group of the Cynipidae, we tried combined-data analyses where we constrained either the Figitidae or the core Figitidae (figitids excluding the gall-associated forms) to be monophyletic. Relationships among cynipids did not change at all in these analyses compared to the unconstrained total-evidence analysis; even the support values were very similar. When the Figitidae were constrained to be monophyletic, the gall-associated lineages (Plectocynipinae, Euceroptrinae and Parnipinae) appeared as a basal polytomy in the family together with the monophyletic core figitids (PP for the latter 0.87).

**Fig 5:**
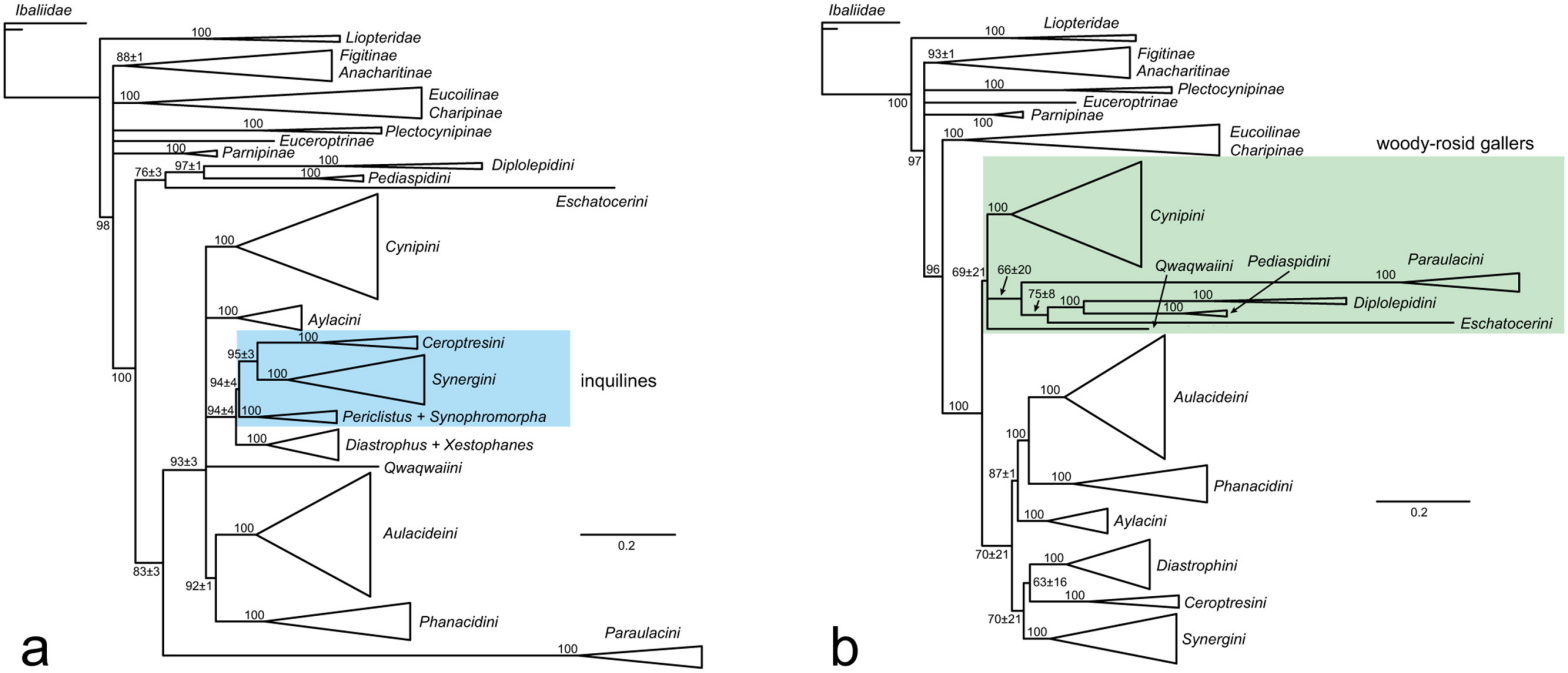
Constrained analyses. Results from analyses in which (a) the inquilines were constrained to be monophyletic or (b) the woody-rosid gallers were constrained to be monophyletic. In both cases, Paraulacini was allowed to ‘float’, that is, it was allowed to be placed either inside or outside the constrained clade.

When we forced cynipid inquilines to be monophyletic (Fig 5a), with Paraulacini allowed to float, the Paraulacini did not group with the rest of the inquilines. Instead, the Synergini and Ceroptresini were nested inside a paraphyletic Diastrophini, similar to the tree from the analysis of the morphological and life-history data (cf. Fig 4). Inside the inquilines, the Synergini + Ceroptresini (PP 0.95) ended up as the sister group to *Periclistus* + *Synophromorpha* (PP 1.00), while *Diastrophus* + *Xestophanes* (PP 1.00) together constituted the sister group of the inquilines. The woody rosid gallers did not end up as a monophyletic group in this analysis. The posterior probability of the inquilines being monophyletic, with or without Paraulacini included, was estimated to be less than 0.001, showing strong evidence against this hypothesis.

Constraining woody rosid gallers to be monophyletic (Fig 5b), again with Paraulacini allowed to float, resulted in support for a clade including all woody rosid gallers and the Paraulacini, but the support was relatively weak (PP 0.69) showing that there was significant posterior probability on trees where Paraulacini was placed outside of the woody rosid gallers. No supertribal groupings within the woody rosid gallers were highly supported. The rest of the cynipid tree was unaffected by the constraint; in particular, the inquilines did not form a monophyletic group. The posterior model probability of the woody rosid gallers being monophyletic, with or without Paraulacini included, was estimated to be less than 0.001, indicating strong evidence also against this hypothesis.

### Ancestral state reconstruction

**Fig 6:**
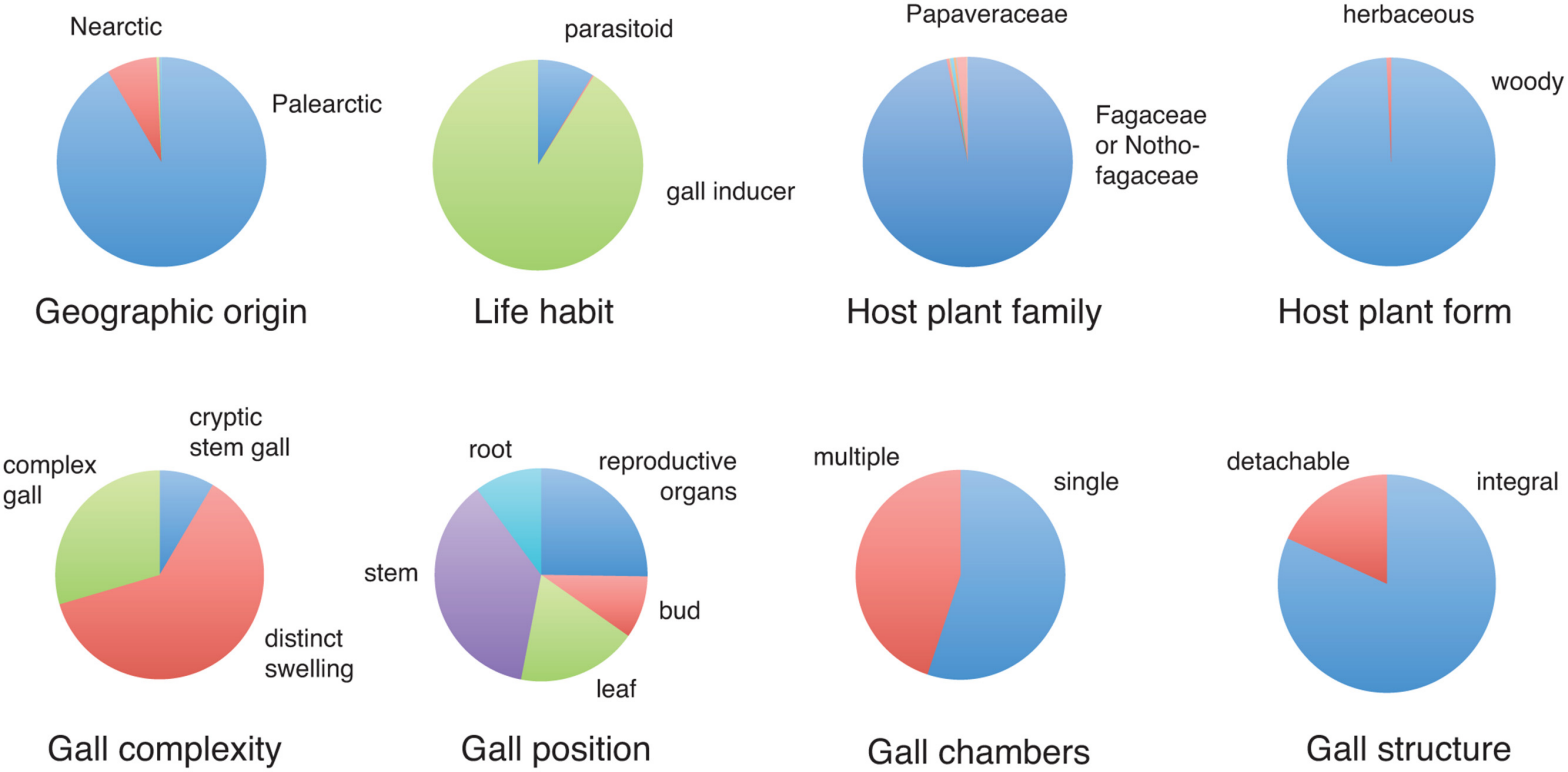
Posterior probability of various biological traits in the ancestor of extant Cynipidae. Note that the ancestor was unlikely to have been an inquiline according to this analysis (PP 0.002).

The inferred ancestral states of the Cynipidae on the total-evidence tree (using all data) (Fig 6) suggest that the most recent common ancestor of gall wasps occurred in the Palearctic (PP 0.92), much less likely the Nearctic (PP 0.08) or the Southern Hemisphere (PP 0.008 for Australia, South Africa and the Neotropics combined). It was a gall inducer (PP 0.91) or possibly a parasitoid (PP 0.087) but very unlikely an inquiline (PP 0.002). The host plant was probably Fagaceae or Nothofagaceae (PP 0.97), or possibly Papaveraceae (PP 0.015), and it was most likely woody (PP 0.99) and not herbaceous (PP 0.007). The evidence is more ambiguous when it comes to gall structure. However, the most likely ancestral gall is a distinct, single-chambered, integral stem swelling (Fig 6).

It is fairly easy to identify distinct morphological or life-history traits of the twelve cynipid tribes, that is, traits that are unusual among the other tribes (Table 3). However, it is more difficult to identify potential unique synapomorphies (Table 4). In particular, it is difficult to find unique synapomorphies for the Diplolepidini, but also the Synergini and Ceroptresini lack traits with a uniqueness index above 0.50. The Eschatocerini stand out as having the largest number of unique autapomorphies, but also the Pediaspidini, Qwaqwaiini and Paraulacini have a large number of unique or highly unusual features. Remaining tribes are intermediate in terms of their distinctness and uniqueness. Individual traits are discussed in more detail in the taxonomic section of the paper.

**Table 3:** Most distinctive traits of the 12 cynipid tribes recognized in this paper.

Tribe	Traits in order of distinctness index^1^					
Cynipini	63:1 (0.86)	28:2 (0.85)	120:1 (0.82)	39:1 (0.76)	156:1 (0.75)	54:1 (0.72)
Diplolepidini	233:1 (0.88)	108:1 (0.87)	110:1 (0.84)	104:1 (0.76)	59:2 (0.75)	166:1 (0.75)
Pediaspidini	88:1 (1.00)	15:1 (0.98)	66:1 (0.95)	70:1 (0.93)	122:1 (0.93)	62:1 (0.93)
Eschatocerini	84:3 (1.00)	136:0 (1.00)	233:2 (1.00)	8:1 (1.00)	132:3 (1.00)	139:1 (1.00)
Qwaqwaiini	176:1 (1.00)	229:4 (1.00)	41:1 (0.96)	180:1 (0.95)	137:0 (0.93)	216:1 (0.92)
Paraulacini	227:1 (0.94)	152:1 (0.92)	60:0 (0.92)	229:2 (0.91)	216:1 (0.85)	238:1 (0.85)
Aylacini s. str.	233:7 (0.99)	36:1 (0.85)	234:1 (0.83)	236:0 (0.82)	51:2 (0.79)	14:1 (0.76)
Aulacideini	106:1 (0.94)	91:2 (0.92)	234:1 (0.83)	14:1 (0.80)	93:1 (0.78)	29:0 (0.78)
Phanacidini	70:3 (0.95)	233:5 (0.88)	91:1 (0.85)	90:2 (0.84)	234:1 (0.83)	55:2 (0.83)
Diastrophini	233:1 (0.91)	49:1 (0.89)	124:1 (0.83)	59:0 (0.82)	3:1 (0.77)	125:1 (0.77)
Synergini s. str.	144:1 (0.87)	18:1 (0.82)	22:1 (0.81)	230:1 (0.79)	91:2 (0.76)	198:1 (0.73)
Ceroptresini	230:1 (0.80)	124:1 (0.77)	235:0 (0.75)	125:1 (0.74)	3:1 (0.74)	22:1 (0.73)

The traits were ranked according to their distinctness index, measured as the probability of the tribe having the trait as ancestral subtracted by the average probability of the other tribes and the Cynipidae having the trait as ancestral (see Materials and Methods for details). A unique autapomorphy for a higher taxon would have a distinctness index of 1.0. A complete list of distinctness values is provided in S2 Table.

^1^Each entry specified as character number: state number (distinctness index value).

**Table 4:** Most unique traits of the 12 cynipid tribes recognized in this paper.

Tribe	Traits in order of uniqueness index^1^					
Cynipini	28:2 (0.83)	130:2 (0.60)	120:1 (0.39)	156:1 (0.36)	32:0 (0.11)	47:0 (0.00)
Diplolepidini	173:1 (0.38)	83:0 (0.30)	168:1 (0.29)	128:1 (0.20)	45:3 (0.15)	129:1 (0.14)
Pediaspidini	88:1 (1.00)	233:4 (0.92)	15:1 (0.86)	70:1 (0.75)	7:2 (0.55)	66:1 (0.50)
Eschatocerini	84:3 (1.00)	136:0 (1.00)	233:2 (1.00)	8:1 (0.99)	132:3 (0.98)	139:1 (0.97)
Qwaqwaiini	176:1 (1.00)	229:4 (0.99)	137:0 (0.69)	4:1 (0.64)	180:1 (0.53)	41:1 (0.50)
Paraulacini	227:1 (0.94)	152:1 (0.86)	19:2 (0.74)	60:0 (0.74)	193:1 (0.70)	230:0 (0.33)
Aylacini s. str.	233:7 (0.96)	51:2 (0.41)	2:1 (0.18)	236:0 (0.07)	36:1 (0.04)	138:1 (0.02)
Aulacideini	106:1 (0.66)	29:0 (0.54)	233:6 (0.49)	148:1 (0.24)	91:2 (0.14)	64:0 (0.10)
Phanacidini	70:3 (0.74)	84:2 (0.40)	20:1 (0.10)	132:1 (0.08)	38:0 (0.07)	47:1 (0.05)
Diastrophini	49:1 (0.61)	148:2 (0.06)	21:1 (0.06)	164:0 (0.06)	52:0 (0.06)	124:1 (0.05)
Synergini s. str.	76:0 (0.48)	57:2 (0.44)	18:1 (0.39)	187:1 (0.39)	189:1 (0.39)	191:1 (0.39)
Ceroptresini	235:0 (0.48)	90:0 (0.38)	160:1 (0.35)	43:1 (0.27)	109:1 (0.22)	146:1 (0.21)

The traits were ranked according to their uniqueness index, measured as the probability of the tribe having the trait as ancestral subtracted by the maximum probability that the trait was ancestral in the other tribes or in the Cynipidae (see Materials and Methods for details). A unique autapomorphy for a higher taxon would have a uniqueness index of 1.0, and the value decreases more rapidly than the distinctness index if the trait is shared with one or more other tribes or if it is likely to be ancestral in the Cynipidae. A complete list of uniqueness values is provided in S2 Table.

^1^Each entry specified as character number: state number (uniqueness index value).

## Discussion

### Cynipid monophyly

It is somewhat surprising that the molecular data presented here do not support the monophyly of Cynipidae as currently circumscribed. However, when individual gene trees are examined (S1–S5 Figs), it is obvious that the signal that conflicts with cynipid monophyly is weak and inconsistent across genes. Mixed figitid and cynipid groups only occur in the COI and EF1aF2 trees, they do not include the same taxa, and they have PP in the range 0.64–0.68. The 28S and LWRh trees are not informative on the question of cynipid monophyly, and the EF1aF1 tree actually supports monophyly quite strongly (PP 0.77). It is no surprise then that the Cynipidae appear as monophyletic when molecular data are combined with morphological and biological data.

It might be tempting to conclude that the gall-forming habit itself plays an important role in forcing cynipid monophyly in our analyses, but this idea can be safely dismissed. First, there is considerable variation among basal cynipid lineages in general life history (eight clades are gall formers, two are inquilines, and two are of uncertain origin; see Fig 2). Second, because our coding treats all transitions among parasitic, inquilinous and gall-inducing forms as equally likely, it does not provide any evidence for grouping inquilines and gall inducers into a monophyletic Cynipidae. Finally, excluding all life-history characters from the analysis does not remove the support for cynipid monophyly (S7 and S8 Figs). In conclusion, then, we see no reason, based on our results, to doubt that gall wasps form a natural group.

### The origin of cynipids

**Fig 7:**
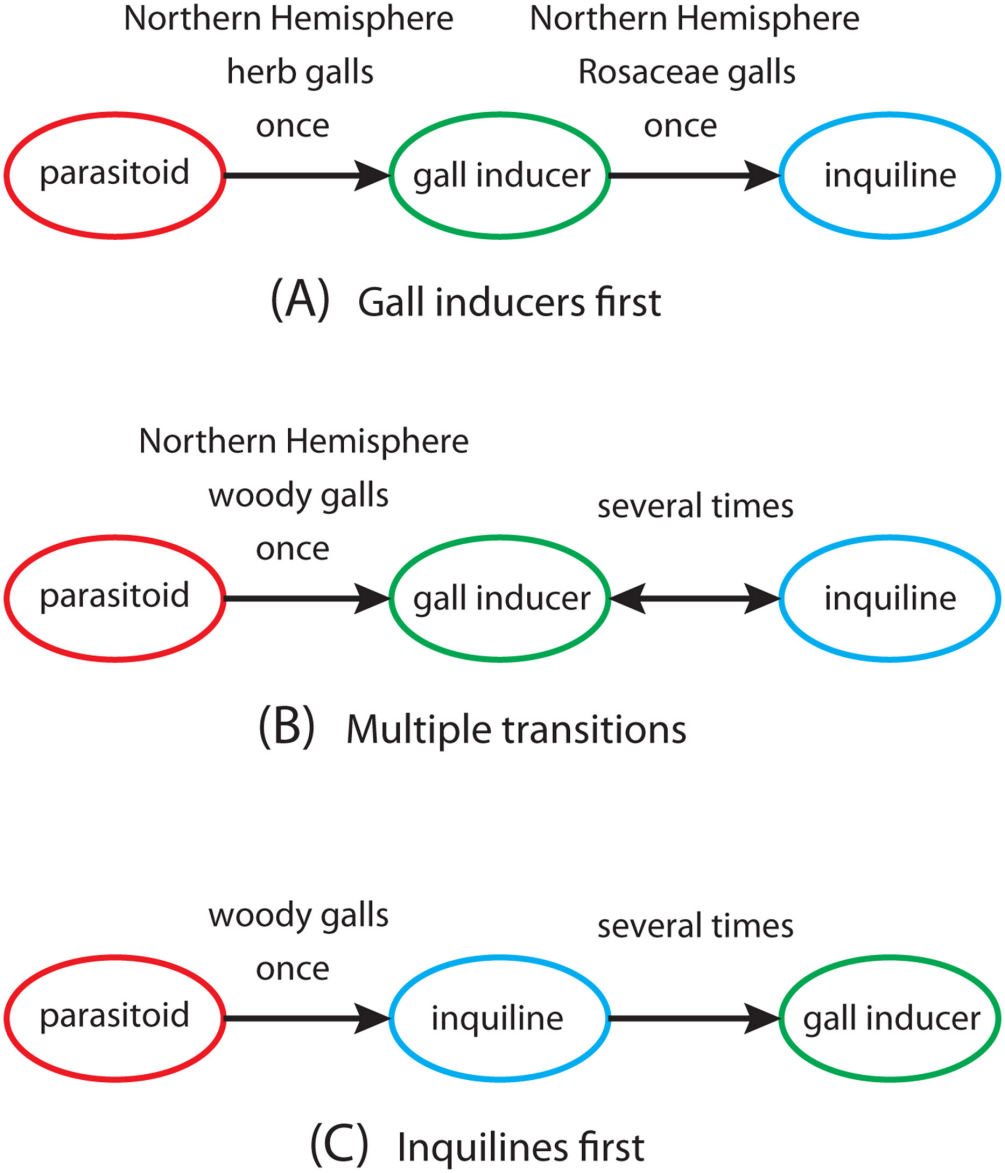
Different scenarios for the origin of cynipid gall inducers and inquilines. In the gall inducers first scenario supported by previous morphology-based analyses (A), gall wasps originated in the Northern Hemisphere and were originally herb gallers. The inquilines evolved once from Rosaceae gallers. The current study supports a multiple transitions scenario (B), in which the first gall inducers were associated with woody hosts. This was followed by multiple transitions between gall inducers and inquilines. The alternative inquilines first scenario (C), which has become more plausible given recent discoveries even though it is still not the favored one, postulates that the first gall wasps were inquilines, possibly in the Southern Hemisphere, and that gall inducers evolved independently in several lineages of cynipids.

The most surprising results of the present analysis concern the origin and early evolution of gall wasps. Previous studies based on morphology have painted a rather complete picture that can be described as the “gall inducers first” scenario (Fig 7A). It holds that gall wasps evolved in the Palearctic from parasitoids of other gall inducers [29]. The first gall wasps were likely associated with herbaceous plants, possibly in the Papaverales lineage of primitive eudicots, and they induced single-chambered galls in the reproductive structures (fruits or seed capsules) of their host plants. Most cynipid gallers of woody host plants originated from a species-rich monophyletic clade that appeared later, the woody-rosid gallers. The inquilines evolved once from Rosaceae gallers (*Diastrophus*, *Xestophanes*) unrelated to the main clade of woody-rosid gallers [27]. The inquilines apparently lost the ability to initiate galls, and initially parasitized their closest relatives, but later radiated to exploit several different lineages of woody-rosid gallers [27–29].

The results of the current study (Figs 2 and 6) conflict in several ways with gall inducers first, and instead support a scenario that might be called “multiple transitions” (Fig 7B). It holds that the ancestral cynipids were associated with woody host plants and not herbs, and that the woody-rosid gallers do not represent a recent radiation but instead comprise some of the oldest cynipid lineages. Even though the phytophagous habit arose once, there must have been multiple origins of inquilines, gall inducers or both. Some inquilines (*Periclistus* and *Synophromorpha*) are indeed closely related to Rosaceae gallers (*Diastrophus* and *Xestophanes*), as suggested by morphology-based analyses but they form the basal lineages in this clade (the Diastrophini), suggesting that the ancestors of the clade were inquilines and not gall inducers.

An alternative scenario that is at least consistent with some of our results could be described as “inquilines first”. According to this scenario, gall wasps were originally inquilines; gall inducers then originated independently several times in different lineages of inquilines (Fig 7C). The relationships within the Diastrophini clearly seem to support an inquilines first scenario, and there is additional support for the scenario in the Synergini, as detailed below. It is at least conceivable that additional discoveries of basal cynipid lineages would support a single origin of inquilines from parasitoids at the base of the cynipid tree, thus challenging the multiple transitions scenario by removing all transitions in the other direction, from gall inducers to inquilines. If so, it is unclear whether the inquiline stem form originated in the Northern or Southern Hemisphere.

There are two major reasons for the shift in support for the different scenarios. First, we now have much better taxon sampling and more information on the biology of basal cynipid and figitid lineages, including several rare and isolated taxa from the Southern Hemisphere. These include the Paraulacini and Qwaqwaiini among the cynipids, and the Euceroptrinae and Plectocynipinae among the gall-associated lineages in the Figitidae, the sister group of the Cynipidae. Unfortunately, our analysis did not include Thrasorinae and Mikeiinae, two additional subfamilies of gall-associated figitids from the Southern Hemisphere. Although we still have scant information on the biology of these lineages, all of them are associated with woody rosids (Table 1). The Paraulacini are phytophagous inquilines, or possibly parasitoids, in chalcidoid galls on southern beech, *Nothofagus*, in southern South America [6], and the Qwaqwaiini are gall inducers on *Scolopia* (Salicaceae) in South Africa [6]. The gall-associated figitids are all parasitoids in chalcidoid galls (Table 1), or so it is presumed in the absence of evidence to the contrary. Second, the molecular data contradict the resolution of basal cynipid lineages suggested previously by morphology. Even though it is based on substantially better taxon sampling and more sequence data, our present study largely confirms the early molecular results obtained by Nylander et al. [32].

Although our ancestral state reconstructions (Fig 6) still support a cynipid origin in the Northern Hemisphere, it may turn out that a Southern Hemisphere origin is more likely when more data become available. There are very few gall wasps known from the Southern Hemisphere and some of them have only been described or characterized very recently. It is striking, then, that all known Southern Hemisphere cynipids apparently form isolated basal lineages in the family (Fig 2). Future exploration of the poorly known cynipid fauna of the Southern Hemisphere may well result in the discovery of more of these taxa, helping tip the balance in analyses of the geographic origin of gall wasps to that part of the world.

When assessing the conclusion that cynipids were originally gall inducers and not inquilines (Figs 6 and 7B), one should bear in mind that it depends to a large extent on assumptions concerning the evolution of this life-history trait. If one were willing to assume that the transition from parasitoids to gall inducers did not occur instantaneously but likely involved some intermediate stage when the ancestors were inquilines, then the most likely inferred ancestral state of cynipids might well be inquilinism. The inferred ancestral state may also change when the relationships among cynipid tribes are better resolved. Thus, it is too early to dismiss the possibility that inquilines are usually ancestors of gall inducers, as postulated by inquilines-first, and not derived from them.

### The Papaveraceae connection

If it is true that gall wasps were originally associated with galls on woody host plants, as our results suggest, then the association of some basal cynipid and figitid lineages with Papaveraceae must at least partly be convergent. Among the gall-associated figitids, the Mediterranean *Parnips* is undoubtedly the most cynipid-like [8]. It is a parasitoid of *Barbotinia* gall inducers in seed capsule galls on poppies, *Papaver* (Ronquist, Nieves-Aldrey, Vårdal and Nylander, unpublished data). *Barbotinia* is the sister-group of all other taxa in the Aylacini sensu stricto, possibly one of the most basal cynipid lineages (Fig 2). *Parnips* and *Barbotinia* are so remarkably similar morphologically that it is difficult to escape the impression that they are ‘living-fossil’ remnants of an early Papaveraceae-associated microcynipoid (figitid + cynipid) fauna [8]. Intriguingly, the Papaveraceae belong to the Ranunculales, one of the earliest offshoots in the eudicot clade [30], which includes the host plants of virtually all other cynipids and gall-associated figitids, and it is quite possible that the Papaveraceae were more dominant among angiosperm lineages when cynipids and figitids originated than they are today.

Since the earlier morphology-based studies of cynipid relationships, Papaveraceae-associated taxa have also unexpectedly appeared in one of the other major cynipid clades, the Aulacideini. For a long time, the host plant of *Neaylax versicolor* in the Aulacideini was unknown, but it was recently discovered [50] that it galls *Fumaria*, previously classified in the Fumariaceae but now included among the Papaveraceae [30]. Our analysis shows that *N*. *versicolor* is closely related to *Aylax hypecoi*, a galler of *Hypecoum* (also in Papaveraceae), and that the current generic placement of both taxa is incorrect. Herb galls are undoubtedly less well studied than tree and bush galls, and it is quite possible that future studies will turn up more herb-associated basal figitid and cynipid lineages, possibly challenging the current conclusion that the first cynipids were associated with woody host plants.

### The evolutionary significance of inquilines

The inquilines share many morphological traits, partly explaining why they tend to be supported as a monophyletic group in morphology-based analyses. For instance, *Periclistus*, *Ceroptres*, and *Rhoophilus* are basal members of the three major inquiline clades identified here: the Diastrophini (also including some gall makers), the Ceroptresini and the Synergini (sensu stricto), respectively. Females of all three genera have 12 antennal articles and a strongly reduced third abdominal tergum (fused to the fourth in *Periclistus* and *Rhoophilus*), highly unusual traits among gall-inducing cynipids and also rare in figitids. Given the results of the current study, one would have to conclude that these are convergent similarities, even though it is difficult to see how they could be related to the larval habit of being an inquiline.

The uncertainty concerning basal splits in the Cynipidae in our analyses leave a possibility that the inquiline tribes (Synergini, Ceroptresini, and Diastrophini) actually form a monophyletic group but such a clade would also include the Rosaceae gallers *Diastrophus* and *Xestophanes* in addition to the inquilines, and very unlikely in a basal position given the strong molecular signal across multiple genes placing them deeply within the Diastrophini.

Thus, regardless of the relationships among cynipid tribes, our results clearly imply multiple transitions between gall inducers and inquilines. However, the direction of these transitions is unclear in most cases. The idea of an initial inquiline phase in the origin of gall inducers (Fig 7C) is quite appealing in that it leaves much more time for the origin of the complicated adaptions necessary to control plant development well enough to induce galls [19, 26]. There is also some evidence suggesting that several groups of cynipid inquilines may originally have been associated with non-cynipid galls, as required by a scenario in which they are ancestral to cynipid gall inducers rather than secondarily derived from them. For instance, we now know that *Rhoophilus*, a basal member in Synergini sensu stricto, is an inquiline in lepidopteran galls [51]. The Paraulacini are inquilines (or parasitoids) in chalcidoid galls [6], and there are also records of *Ceroptres* and *Periclistus* attacking non-cynipid galls, even though these records are poorly documented and often contested. An additional observation that seems to support multiple origins of gall inducers from inquilines is the recent discovery of a gall-inducer in the large inquiline genus *Synergus*, deeply nested within the inquiline tribe Synergini sensu stricto [52]. Multiple origins of gall-inducing cynipids might appear unlikely at first sight, given that gall inducers are completely absent from the rest of the superfamily Cynipoidea. However, gall inducers have obviously evolved independently multiple times in the Apocrita, primarily in the Chalcidoidea [53] but also in the Braconidae [54], so the idea that it happened repeatedly in the Cynipidae certainly merits serious consideration.

The transition in the other direction, from gall inducer to inquilines, also appears quite plausible. Inquiline larvae develop in larval chambers of their own inside the host gall, and many of them modify the host gall significantly. Thus, they are sometimes described as gallers of galls, and it is easy to imagine how such a specialized habit could evolve from primary gall inducers. If the first cynipids were gall inducers, as our results suggest, then there must have been at least one transition from gall inducers to inquilines (Fig 7B). Even that conclusion, however, could easily change when basal cynipid relationships are better resolved. More definite conclusions on the exact life-history transitions that occurred in the early radiation of cynipids will thus have to await future studies.

### Woody-rosid gallers: monophyletic or paraphyletic?

Our conclusion that the woody-rosid gallers form a paraphyletic or polyphyletic assemblage of basal cynipid lineages rhymes well with the extreme morphological, molecular and geographical diversity of woody-rosid gallers, exceeding the diversity of all other cynipids taken together. The Cynipini alone may represent the largest radiation of phytophagous insects onto a single host plant genus, with more than 1,000 species almost exclusively specialized on oaks (*Quercus*) [55]. The Pediaspidini, Eschatocerini, Diplolepidini, and Qwaqwaiini are species-poor but highly morphologically (and genetically) distinct lineages, suggesting that they are the products of a long time of evolution in isolation. If the woody-rosid gallers form a secondarily derived, monophyletic group, as suggested by previous studies, then morphological and molecular evolution must have been significantly accelerated in all of these lineages.

Is it possible that evolution has been accelerated to such a dramatic extent in woody-rosid gallers? In fact, there may be a biological explanation for such a phenomenon. Because trees and bushes are more long-lived than herbs, they provide more stable environments, which might be associated with increased spatial structure in populations and elevated diversification rates. Elevated evolutionary rates may lead to long branches in the phylogeny of cynipids, possibly causing phylogenetic inference errors through the phenomenon of long-branch attraction (LBA). A much-cited LBA review [56] specifically mentions the cynipid phylogeny study of Nylander et al. [32] as a possible example of the phenomenon, noting that removal of the outgroup from the analysis resulted in a tree that could be rooted such that both woody-rosid gallers and inquilines were monophyletic. It was concluded that the cynipid dataset was influenced by LBA between the outgroup and some of the woody-rosid gallers.

**Fig 8:**
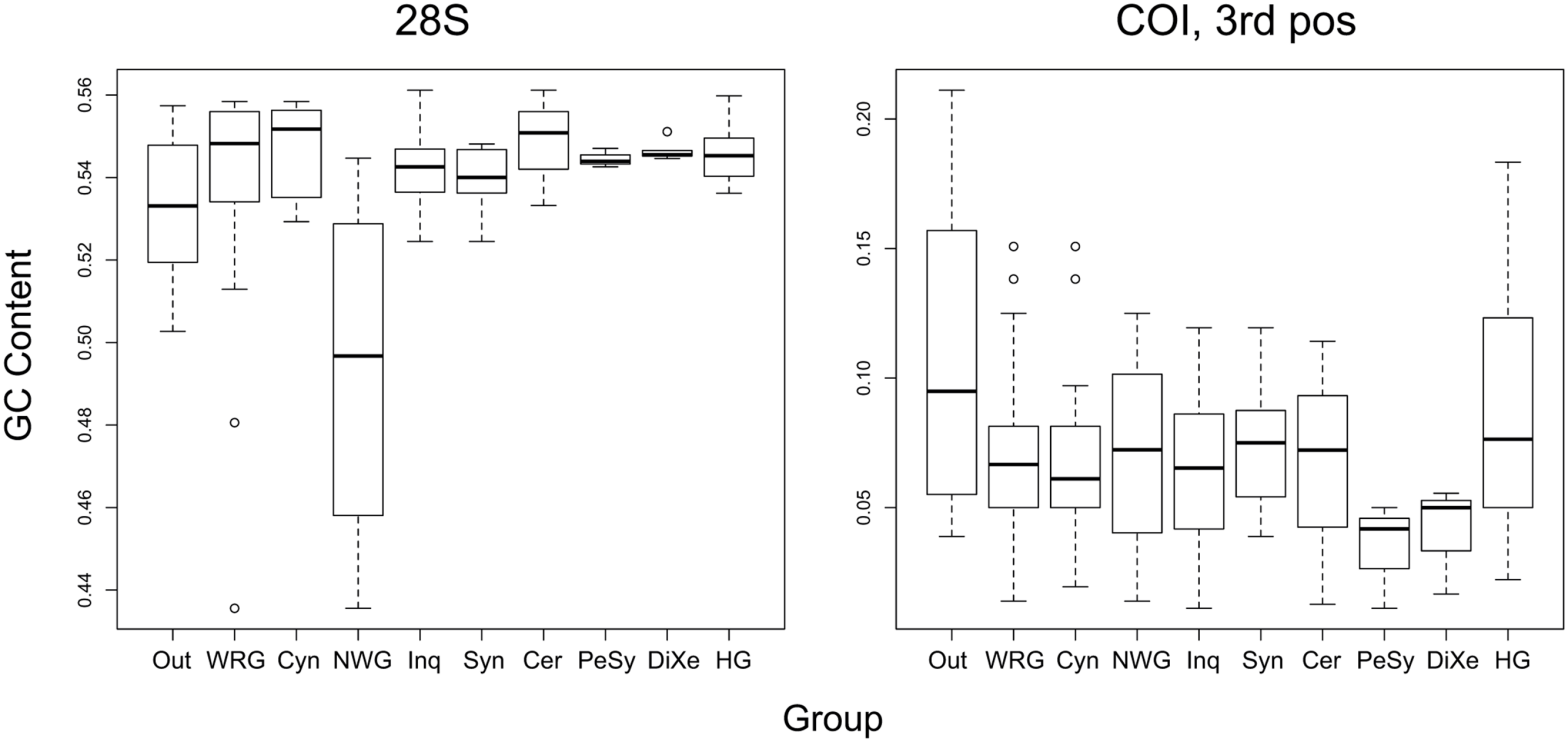
Base composition bias and cynipid phylogeny. GC composition bias across the tree is unlikely to explain why woody-rosid gallers and inquilines, supported as monophyletic in previous morphology-based analyses, do not appear as monophyletic groups in our analyses. To exemplify, we show the GC composition bias for 28S (left) and COI (right). For the latter, we only show the third codon position, containing almost all of the informative variation; the pattern was similar but less extreme for the other codon positions. The non-oak woody-rosid gallers (NWG) are more similar to the outgroups (Out) than to the Cynipini (Cyn) in GC content of 28S, possibly a convergent similarity explaining why the woody-rosid gallers (WRG) are not monophyletic in the 28S analysis. There is no such potential bias in COI but the WRG nevertheless fail to appear as monophyletic in separate analysis of this marker. For the inquilines, the pattern is the reverse. In COI, there is some heterogeneity in composition among inquilines (Inq), such that *Periclistus* and *Synophromorpha* (PeSy) deviate from the Synergini (Syn) and Ceroptresini (Cer). Similarly, *Diastrophus* and *Xestophanes* (DiXe) deviate from herb gallers (HG, including Aylacini, Aulacideini and Phanacidini). If the bias arose independently in the two groups, it could explain the support for Diastrophini in the COI analysis. There is no such bias in 28S but the Diastrophini nevertheless appear as monophyletic in analysis of this marker.

The considerably larger taxon sample in the present study should have helped resolve LBA issues, but instead of decreasing the conflict between morphology and molecules, the expanded dataset supports the previous results. Almost all of the phylogenetic signal in the present study comes from loop regions of 28S and from third-codon-position sites of protein-coding genes, characters believed to be largely neutral and less affected by LBA than strongly selected sites. Compositional bias, especially bias that varies across sites or across the tree, could increase the risk of LBA artifacts [57]. We examined whether differences in GC content across the tree might bias against monophyly of the woody-rosid gallers or the inquilines. There is indeed compositional bias in some data partitions that would be compatible with this hypothesis (Fig 8), but the evidence against the monophyly of woody-rosid gallers and inquilines is equally strong in data partitions where the GC content is homogeneous in the critical taxa (Table 2).

Rate heterogeneity across the tree in different data partitions could also cause LBA, but since individual gene trees largely support the same clades, such heterogeneity would have to be within genes and nevertheless cause similar errors across genes, an improbable albeit not impossible scenario. In conclusion, it seems unlikely that there are major errors in the current analysis due to LBA.

### Evolution of host-plant preferences

Despite the conflicting signals with respect to the origin and early evolution of the Cynipidae, our results largely confirm previous conclusions on the evolution of host-plant preferences in the group. For instance, Ronquist and Liljeblad [29] emphasize the extreme phylogenetic conservatism of host-plant preferences in gall wasps compared to most other phytophagous insects. The molecular and total-evidence results of the current study actually strengthen that result by suggesting minor changes in the tree consistent with host-plant preferences. The molecular data move *Liposthenes* from the Diastrophini, where it was placed in morphology-based analyses, to the Aulacideini, grouping it with other Lamiaceae gallers instead of placing it with Rosaceae gallers. Furthermore, the current results support monophyly rather than paraphyly for the Diastrophini (Rosaceae gallers and inquilines), Aylacini sensu stricto (Papaverales gallers), and Phanacidini (Asteraceae and Lamiaceae gallers).

In some of these cases, reanalysis of the morphological data or more recent morphological work appear to support the molecular signal. For instance, the Aylacini sensu stricto have a unique convoluted process ventrally on the petiole [28] which was previously interpreted as being secondarily lost in some other cynipid lineages, but it is more parsimoniously understood as a synapomorphy of the Aylacini sensu stricto. Another possible synapomorphy of the Aylacini sensu stricto (Nieves-Aldrey unpublished data) is that the salivary opening in the terminal instar larva has the form of a vertical crevice without a surrounding tuberculate area. Furthermore, the placement of *Liposthenes* in Aulacideini is now supported by a putative synapomorphy in larval morphology [58].

Ronquist and Liljeblad [29] also pointed out the remarkable parallel radiation of Aulacideini and Phanacidini lineages onto the same, apparently arbitrary collection of distantly related host-plant lineages. Among others, affected host plants include the genera *Centaurea* (Asteraceae) and *Phlomis* (Lamiaceae). These results are supported also by the current analysis. The reasons for this pattern are unclear, but one particularly intriguing possibility is that there is some form of inter-specific parasitism across lineages involved in facilitating host-plant shifts [29, 59].

## Conclusion

Although the analyses presented in this paper have thrown new light on the higher relationships and early evolution of cynipids, it is clear that additional data are needed to resolve the basal splits in the cynipid tree. Given that the true phylogeny apparently combines a set of short, deep branches with long or extremely long terminal branches, it may take both genomic-scale data and more accurate statistical modeling to resolve these deep relationships. Further efforts to chart the cynipid fauna of little explored corners of the world should also be strongly encouraged; such efforts might in the end be even more important than more molecular data in shedding additional light on the roots of the cynipid tree.

## Taxonomy

We conclude this paper by formally characterizing and naming as tribes all twelve major clades of cynipids that consistently appeared as strongly supported across gene partitions in our analyses. Many of them have also appeared in previous studies of cynipid relationships [27, 28, 32]. Although we are still unable to resolve the relationships among them, they obviously form natural units, and future discussion will benefit from having proper taxonomic names for these morphologically, biologically and geographically rather cohesive groups of gall wasps.

**Fig 9:**
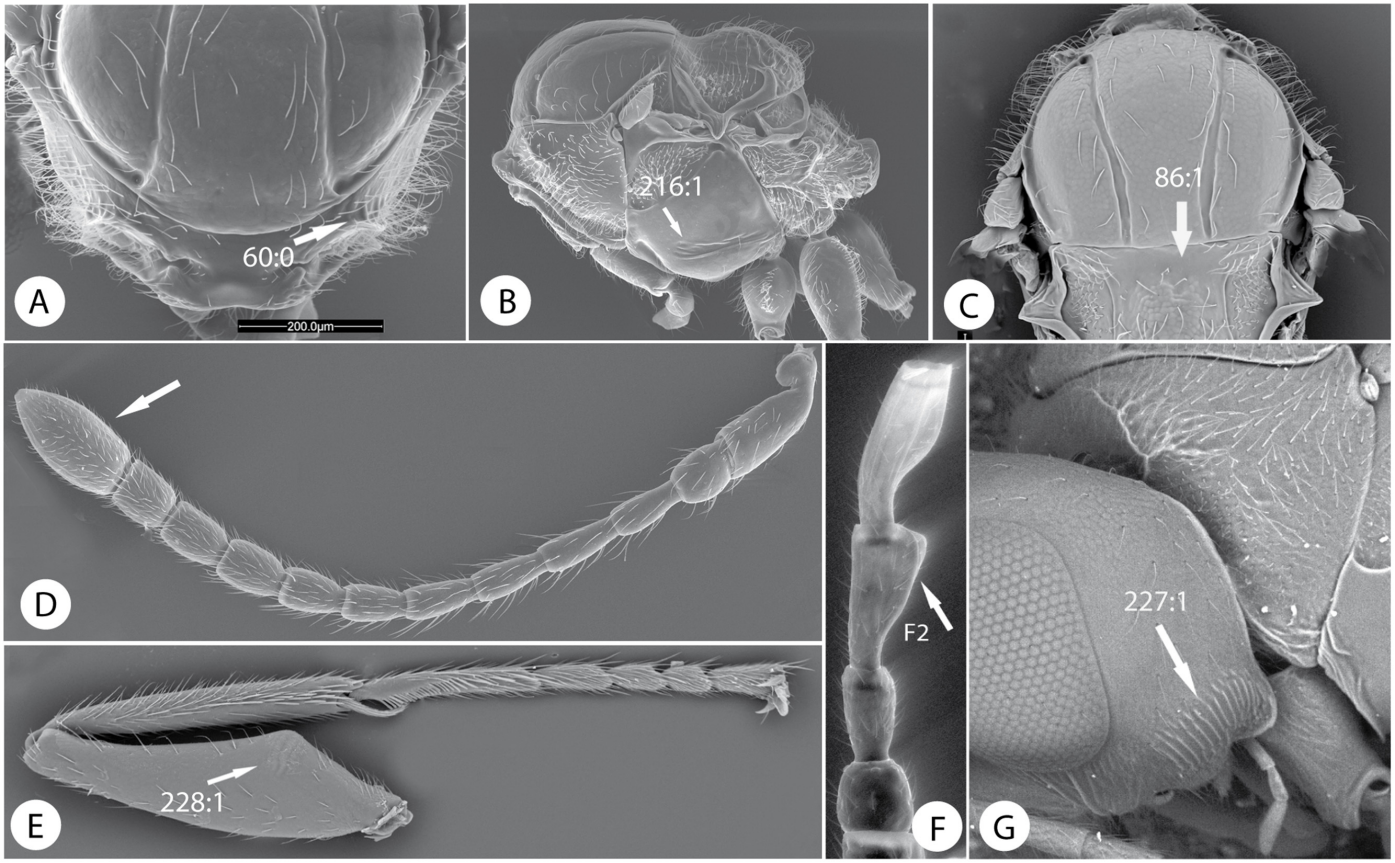
Morphological characters of Paraulacini. *Cecinothofagus gallaelenga* Nieves-Aldrey & Liljeblad: A) pronotum, anterior view. B) mesosoma lateral view. C) mesosoma dorsal view. D) antenna of female. E) profemur. F) first flagellomeres of male antenna. G) head lateral view, showing vertical carinae on gena.

**Fig 10:**
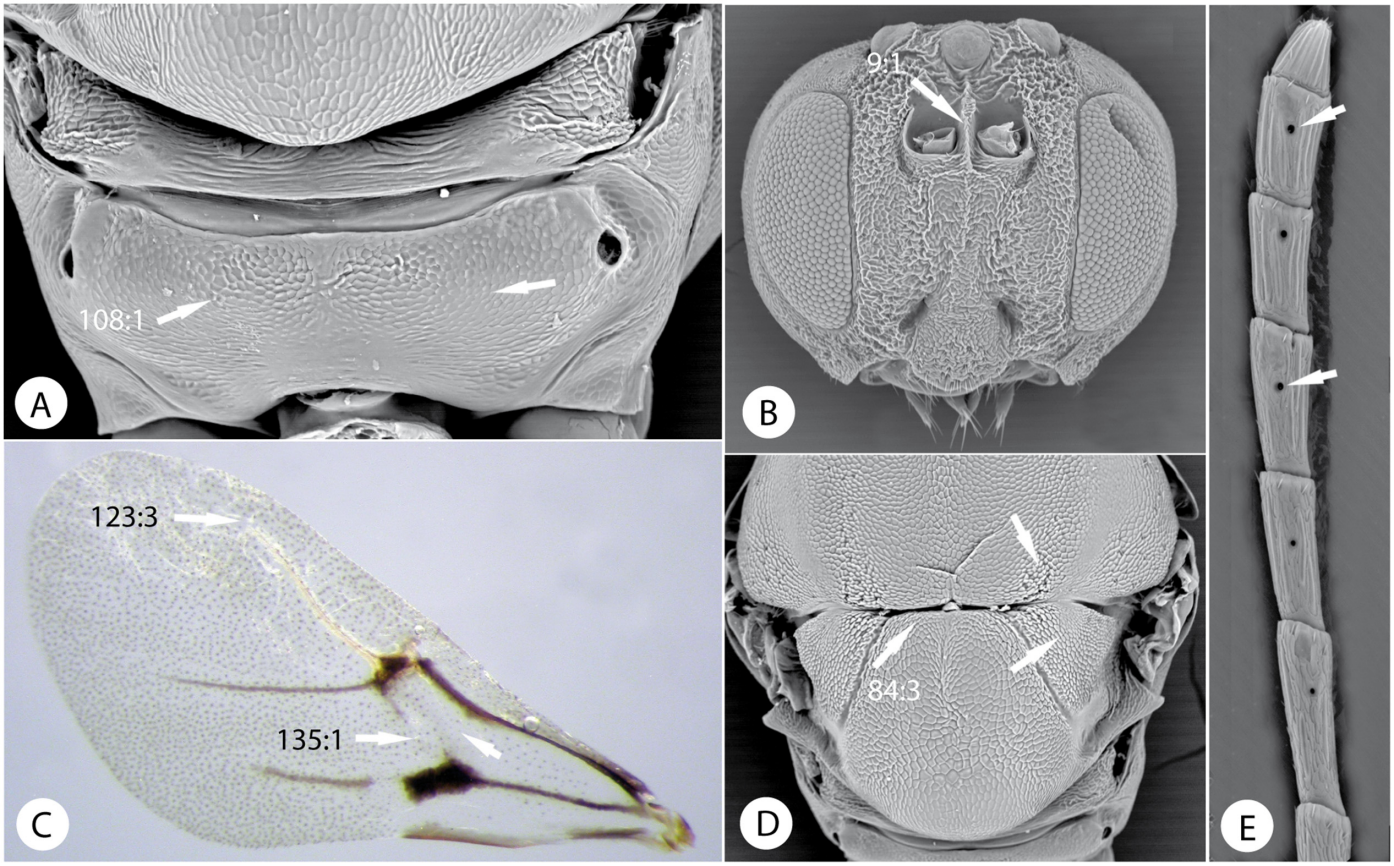
Morphological characters of Eschatocerini. *Eschatocerus acaciae* Mayr: A) propodeum. B) head, anterior view. C) fore wing. D) mesoscutellum in dorsal view. E) last flagellomeres of female antenna.

**Fig 11:**
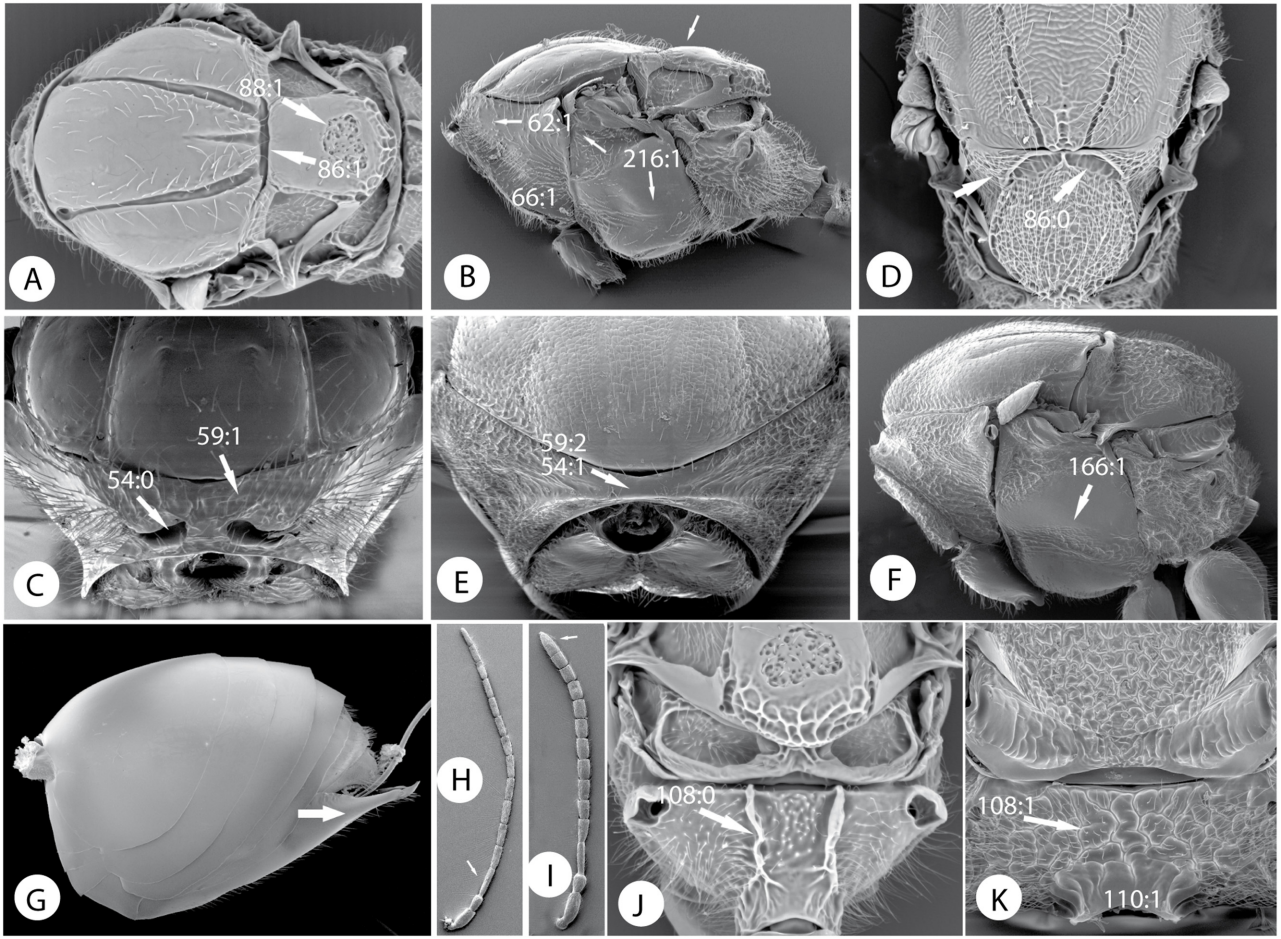
Morphological characters of Pediaspidini, Cynipini and Diplolepidini. A) *Pediaspis aceris* (Gmelin) bisex. gen., mesosoma in dorsal view. B) mesosoma in lateral view. C) pronotum in anterior view. D) *Plagiotrochus razeti* Barbotin parth. gen., mesosoma in dorsal view. E) *Diplolepis rosae* (L.), pronotum in anterior view. F) mesosoma in lateral view. G) *Diplolepis rosae* (L), metasoma in lateral view. H) *Pediaspis aceris*, antenna of male. I) antenna of female. J) *Pediaspis aceris*, propodeum. K) *Diplolepis rosae*, propodeum.

**Fig 12:**
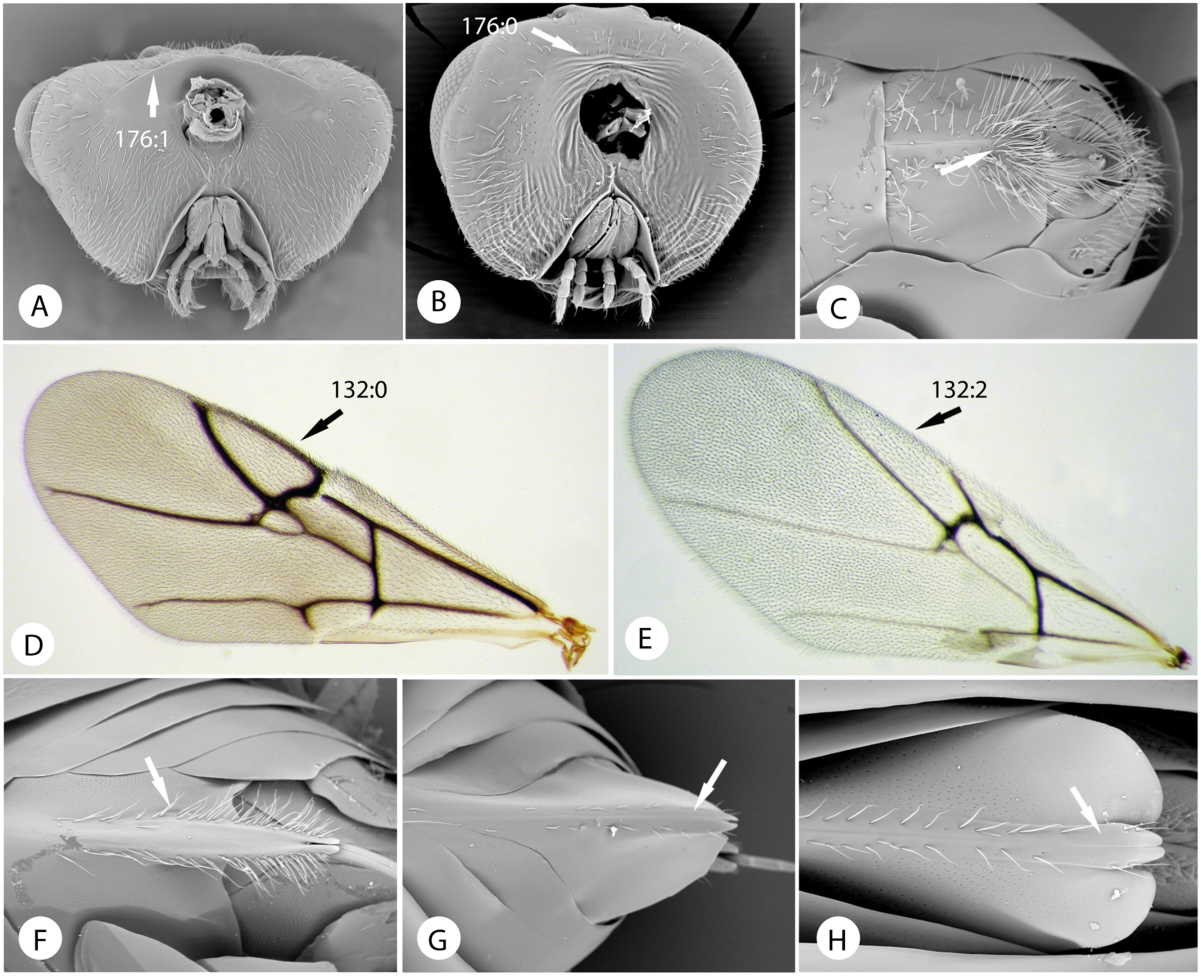
Morphological characters of tribes of Cynipidae. A) *Qwaqwaia scolopiae* Liljeblad Nieves-Aldrey & Melika, head in posterior view. B) *Xestophanes potentillae* (Retzius in De Geer), head in posterior view. C) hypopygium in ventral view and D) fore wing of *Qwaqwaia scolopiae*. E) fore wing of *Plagiotrochus razeti* Barbotin. F) *Amphibolips castroviejoi* Nives-Aldrey & Medianero, hypopygium in ventral view. G) *Aulacidea hieracii* (L.), hypopygium. H) *Synergus ibericus* Tavares, hypopygium.

**Fig 13:**
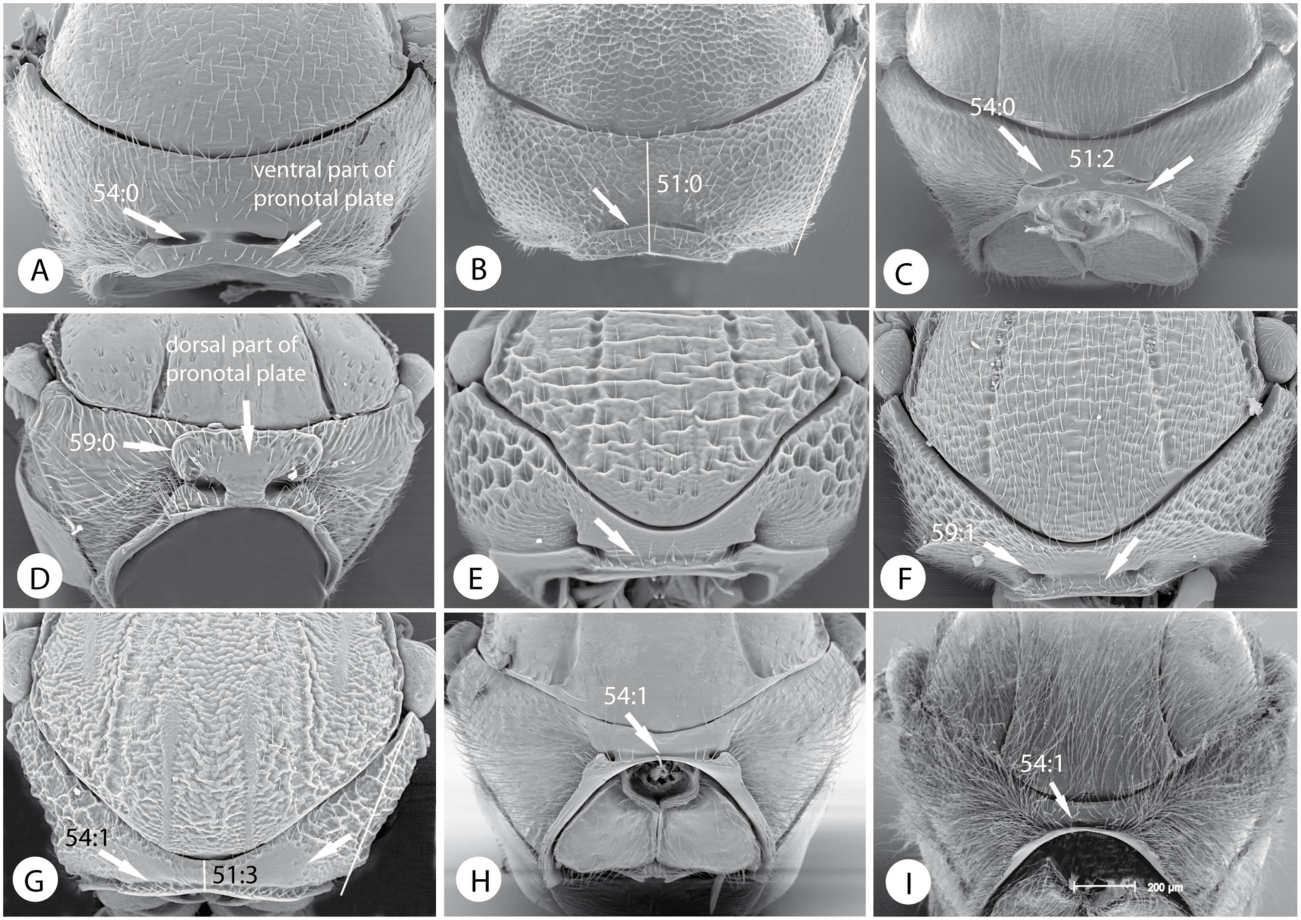
Pronotal chracters of tribes of Cynipidae. Pronotum in anterior view of: A) *Neaylax versicolor* Nieves-Aldrey. B) *Phanacis centaureae* Förster. C) *Aylax papaveris* (Perris). D) *Diastrophus rubi*. E) *Lithonecrus papuanus* Nieves-Aldrey. F) *Synergus ibericus* Tavares. G) *Callirhytis cameroni* Medianero & Nieves-Aldrey. H) *Andricus curvator* Hartig bisex. gen. I) *Cynips divisa* Hartig parth. gen.

**Fig 14:**
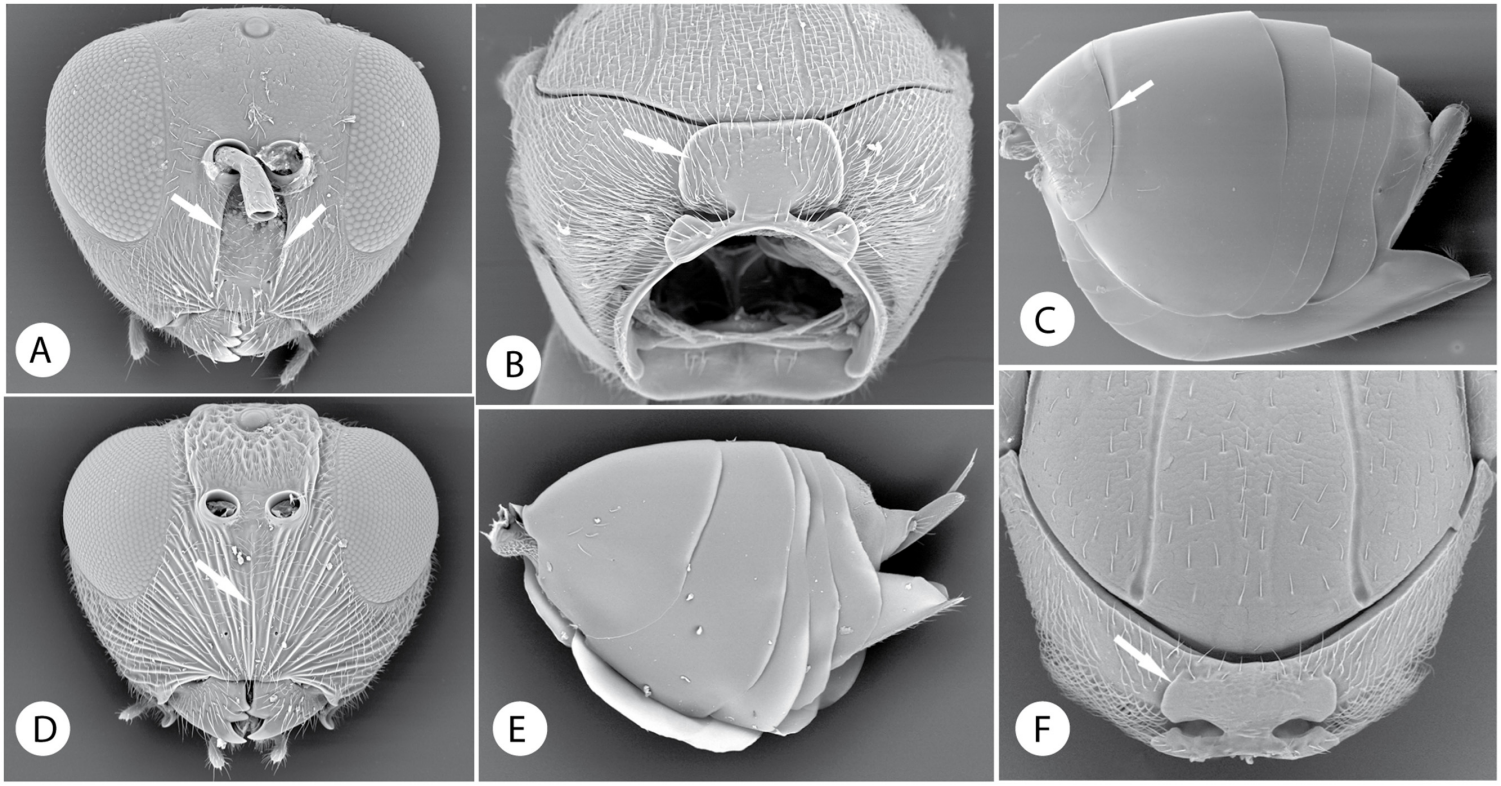
Morphological characters of Ceroptresini, Synergini, Phanacidini and Aulacideini. *Ceroptres clavicornis* Hartig: A) head in anterior view. B) pronotum in anterior view. C) metasoma in lateral view. D) head in anterior view of *Synergus ibericus*. E) metasoma in lateral view of *Phanacis helminthiae* (Stefani). F) pronotum in anterior view of *Liposthenes kerneri* (Wachtl).

**Fig 15:**
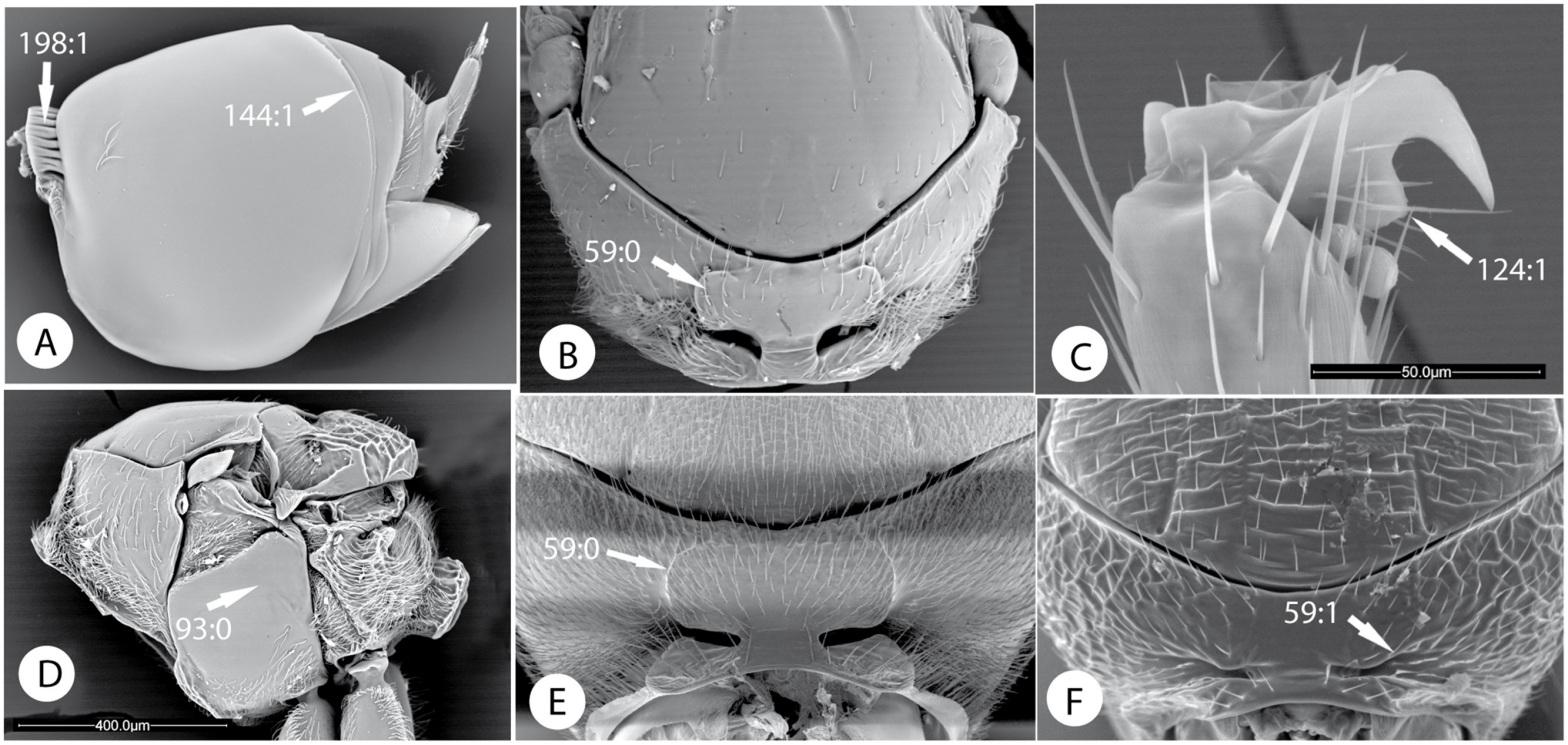
Morphological characters of Diastrophini and Synergini sensu stricto. A) Metasoma in lateral view of *Xestophanes potentillae*. B) pronotum in anterior view of *Xestophanes potentillae*. C) metatarsal claw of *Diastrophus rubi*. D) mesosoma in lateral view of *Xestophanes potentillae*. E) pronotum in anterior view of *Periclistus brandtii* (Ratzeburg). F) pronotum in anterior view of *Rhoophilus loewi* (Mayr).

**Fig 16:**
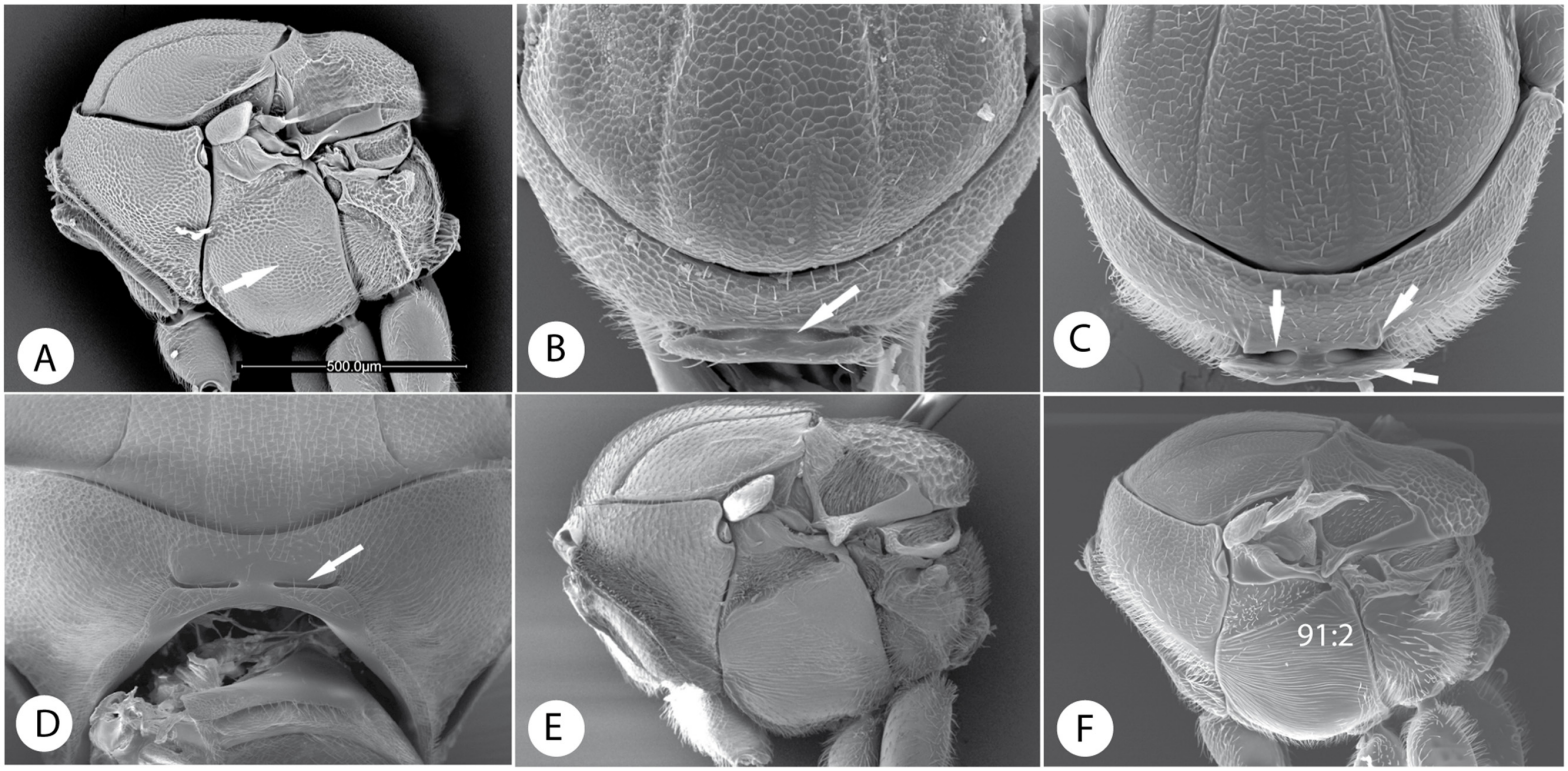
Mesosomal characters of Phanacidini, Aulacideini and Aylacini. A) Mesosoma in lateral view of *Phanacis zwoelferi* Nieves-Aldrey. B) pronotum in anterior view of *Phanacis caulicola* (Hedicke). C) of *Aulacidea martae* Nieves-Aldrey. D) of *Barbotinia oraniensis* (Barbotin). E) Mesosoma in lateral view of *Aylax papaveris*. F) of *Aulacidea martae*.

**Fig 17:**
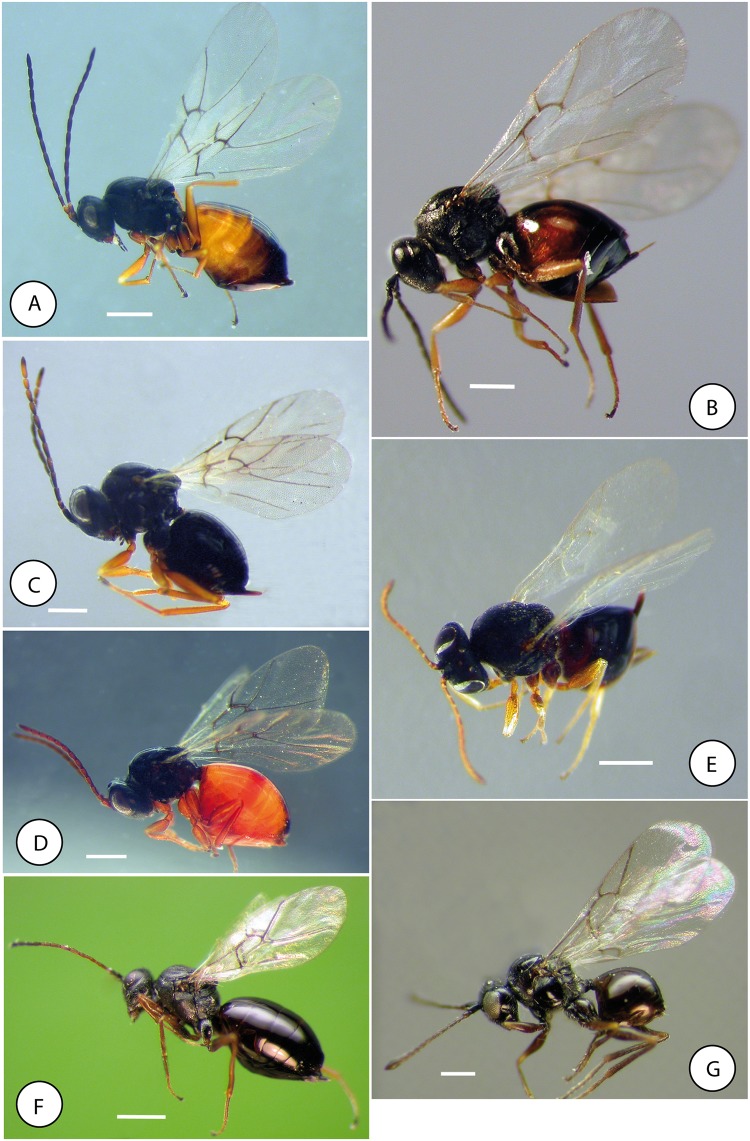
Habitus of exemplar species of tribes of Cynipidae. A) *Iraella luteipes* (Thompson) (Aylacini sensu stricto). B) *Aulacidea hieracii* (Aulacideini). C) *Periclistus brandtii* and D) *Xestophanes potentillae* (Diastrophini). E) *Ceroptres cerri* Mayr (Ceroptresini). F) *Phanacis caulicola* (Phanacidini). G) *Cecinothofagus gallaelenga* (Paraulacini). Scale bar 0.5 mm.

**Fig 18:**
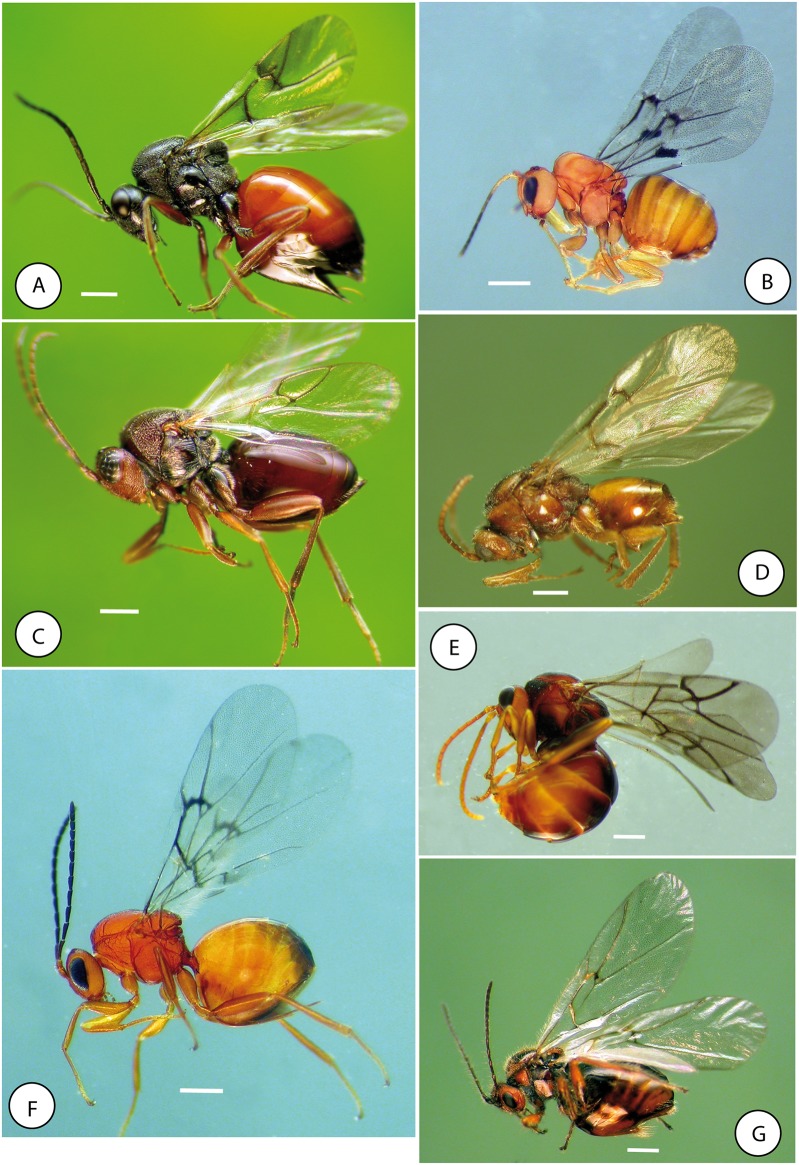
Habitus of exemplar species of tribes of Cynipidae. A) *Diplolepis mayri* (Schlechtendal) (Diplolepidini). B) *Eschatocerus acaciae* (Eschatocerini). C) *Synergus umbraculus* (Olivier) (Synergini sensu stricto). D) *Pediaspis aceris* (Pediaspidini). E) *Qwaqwaia scolopiae* (Qwaqwaiini). F) *Plagiotrochus australis* (Mayr) (Cynipini) and G) *Cynips disticha* Hartig (Cynipini). Scale bar 0.5 mm.

Below, we discuss the characteristic morphological and life-history features of each tribe and summarize what is known about its diversity and distribution. We also give a key to the tribes. Terminology follows [35, 45]. The diagnosis includes the most distinct and unique traits of each tribe according to our analysis (Tables 3 and 4; Figs 9–16). In the description of each tribe, we use numbers to refer to characters and character states in our morphological data matrix (S1 Appendix). For instance, (1:0) refers to character 1 state 0. The habitus of exemplar species of each tribe is presented in Figs 17 and 18.

### Cynipidae

#### Key to adults of the extant tribes of the Cynipidae

1. Female antenna clavate; last flagellomere wider than the penultimate (Fig 9D); male antenna with either F2, F3 or both modified (Fig 9F). Ventral area of gena with 5–9 vertical carinae, genal carina present (Fig 9G). Dorsolateral margin of dorsal part of the pronotal plate strongly projecting laterad (Fig 9A); scutellar foveae shallow or indistinct (Fig 9C); mesopleural impression present, conspicuous (Fig 9B). Profemur with ventral swelling composed of 4–5 rows of sharp, closely spaced, deep costulae (Fig 9E). Inquilines (or parasitoids) in galls induced by Pteromalidae (Chalcidoidea) on *Nothofagus*....................**Paraulacini**

Female antenna filiform or slightly expanded towards apex; last flagellomere not wider than penultimate (Figs 10E and 11I); male antenna not modified or with only F1 modified. Ventral area of gena without vertical carinae. Dorsolateral margin of dorsal part of pronotal plate not projecting laterad (Fig 13C and 13D). Scutellar foveae and mesopleural impression present or absent. Ventral swelling with rows of costulae of profemur absent. Gall inducers on several plants or inquilines in galls of cynipids, excepting one species that is an inquiline in Lepidoptera galls.....................**2**

**2**. Last antennal flagellomeres with conspicuous coeloconic sensillae (Fig 10E). Frons between antennal toruli with a strong longitudinal carina (Fig 10B). Notauli and scutellar foveae absent; dorso-axillar area large, triangular and situated in same plane as mesoscutellum (Fig 10D); lateral propodeal carina completely absent (Fig 10A). Rs+M and R1 of fore wing inconspicuous, radial cell with Rs quite separate from anterior wing margin; basal vein absent (Fig 10C). Neotropical gall inducers on *Acacia* or *Prosopis*....................**Eschatocerini**

Last antennal flagellomeres without conspicuous coeloconic sensillae. Frons between antennal toruli without a strong longitudinal carina. Notauli and scutellar foveae usually present, with dorso-axillar area situated in a different plane compared to the mesoscutellum (Fig 11A and 11D); lateral propodeal carina usually present (Fig 11J). Rs+M and R1 of fore wing usually conspicuous, radial cell with Rs not quite separate from anterior wing margin; basal vein present (Fig 12D and 12E). Gall inducers on other plants or inquilines....................**3**

**3**. Scutellar foveae faint or absent (Fig 11A). Mesopleuron with a mesopleural longitudinal impression (Fig 11B and 11F). Female antenna with 12 or more flagellomeres (Fig 11I); male antenna without modified F1 (Fig 11H). Metatarsal claws simple. Either hypopygium plough-shaped (Fig 11G) or hypopygial spine short. Gall inducers on *Rosa* or *Acer*....................**4**

Scutellar foveae usually well differentiated and deep, sometimes confluent and forming a transverse depression (Fig 11D). Mesopleuron smooth or sculptured but without a mesopleural longitudinal impression (Fig 16A and 16F). Female antenna usually with 10–11 flagellomeres, rarely more; male antenna usually with modified F1. Metatarsal claws simple or toothed. Hypopygium never plough-shaped, either with a short or long hypopygial spine. Gall inducers on other plants or inquilines....................**5**

**4**. Pronotum short dorsomedially, admedian depressions of pronotum not visible, pronotal plate absent (Fig 11E). Mesopleural impression broad, crenulate (Fig 11F). Mesoscutellum dorsally convex, without a rounded impressed area. Lateral propodeal carinae absent (Fig 11K). Hypopygium plough-shaped (Fig 11G). Holarctic gall inducers on *Rosa*....................**Diplolepidini**

Pronotum longer dorsomedially, admedian depressions of pronotum clearly visible and with a conspicuous pronotal plate (Fig 11C). Mesopleural impression linear, narrow, without crenulate sculpture, (Fig 11B). Mesoscutellum dorsally flat with a rounded impressed area (Fig 11A). Lateral propodeal carinae present (Fig 11J). Hypopygium not plough-shaped. Holarctic gall inducers on *Acer* (including the genus *Hymalocynips* from Nepal with biology unknown)....................**Pediaspidini**

**5**. Occiput with strong and sharp occipital carina (Fig 12A). Hypopygium abrupt, not prolonged into a ventral spine; with a dense tuft of long setae (Fig 12C). Radial cell closed (Fig 12D. South African gall inducers on *Scolopia*....................**Qwaqwaiini**

Occiput without distinct and sharp occipital carina, sometimes with some strong parallel occipital rugae (Fig 12B). Hypopygium with more or less distinct, elongated, needle-like ventral spine, with subapical setae only rarely forming a dense tuft (Fig 12F–12H). Radial cell closed or more commonly open on anterior margin of fore wing (Fig 12E)....................**6**

**6**. Basal part of the pronotal plate quite small and very short medially; admedian depressions of pronotum absent, or forming a weak, shallow and continuous depression. Pronotum dorsomedially short, 1/7 or less of length of outer lateral margin (Fig 13G–13I). Holarctic gall inducers on Fagaceae, mainly *Quercus*....................**Cynipini**

Basal part of the pronotal plate usually bigger and more visible; admedian depressions of pronotum usually clearly visible, more or less widely separated (Fig 13A, 13C and 13D). Pronotum dorsomedially longer, 1/5 to 1/3 as long as greatest length of outer lateral margin, rarely shorter but then admedian depressions present (Fig 13C, 13E and 13F)....................**7**

**7**. A distinctly raised vertical carina from ventral margin of each antennal socket present, at least close to antennal socket (Fig 14A). Upper face, vertex and mesopleuron almost smooth. Third abdominal tergum small and free (not fused with the fourth) in both sexes, with a dense hair patch anterolaterally (Fig 14C). Female antenna with 10 flagellomeres. Dorsal part of pronotal plate complete, with its lateral margins marked throughout and reaching anterior margin of mesoscutum (Fig 14B). Radial cell of fore wing closed along anterior margin....................**Ceroptresini**

Distinctly raised vertical carina from ventral margin of each antennal socket absent but sometimes several weaker carinae present in this region (Fig 14D). Upper face, vertex and mesopleuron usually sculptured. Third abdominal tergum either free, but not small (Fig 14E), or fused with the fourth into one large sclerite (Fig 15A); anterolateral hair patch present or not. Female antenna with 10–12 flagellomeres. Dorsal part of pronotal plate usually not complete; lateral margins not reaching anterior margin of mesoscutum (Fig 14F). Radial cell of fore wing open or closed along anterior margin....................**8**

**8**. Abdominal terga 3+4 fused into one large sclerite, occupying nearly entire metasoma, in both sexes (Fig 15A). Dorsal part of pronotal plate incomplete, its lateral margins only marked ventrally (Figs 13E, 13F, and 15F). Head and/or mesosoma usually strongly sculptured. Posterodorsal margin of axillula not marked. Nucha and abdominal tergum 2 usually ring-shaped and longitudinally sulcate (Fig 15A). Metatarsal claws toothed. Inquilines in cynipid galls on Fagaceae or inquilines in Lepidoptera galls on *Rhus* spp., rarely true gall inducers....................**Synergini sensu stricto**

Abdominal terga 3–8 free in most cases (Fig 14E); if terga 3+4 fused in females into one large sclerite then the corresponding terga are not fused in males and the pronotal plate is distinct, with lateral margins marked almost entirely (Fig 15E). Posterodorsal margins of axillula marked. Nucha usually shorter and not sulcate longitudinally; MT1 usually crescent-shaped and smooth (Fig 14E). Metatarsal claws simple or toothed (Fig 15C). Holarctic gall inducers on herbaceous plants or inquilines in galls on *Rubus* or *Rosa*....................**9**

**9**. Dorsal part of pronotal plate distinct, with lateral margins entirely marked (Fig 15B and 15E). Metatarsal claws with an acute basal lobe or tooth (Fig 15C). Female antenna with 10 flagellomeres. Abdominal terga 3–8 either free in the two sexes or 3+4 fused in females, free in males. Mesoscutum and mesopleura usually smooth and shining (Fig 15D), except in the genus *Periclistus*, where they are strongly sculptured. Gall inducers and inquilines associated with Rosaceae....................**Diastrophini**

Dorsal part of pronotal plate usually less distinct, and with lateral margins only marked ventrally (Fig 16C). Metatarsal claws simple. Female antenna with 10–12 flagellomeres. Abdominal terga 3–8 always free, terga 3+4 not fused in either sex (Fig 14F). Mesoscutum and mesopleura usually sculptured (Fig 16A and 16F)....................**10**

**10**. Admedian depressions of pronotum indistinct and shallow, sometimes united medially by a transverse impression (Figs 13B and 16B). Dorsal part of pronotal plate absent (Fig 13B). Mesopleuron with reticulate or rugulose-striate sculpture (Fig 16A). R1 clearly reaching anterior margin of wing; radial cell closed at least partially on anterior margin. Gall inducers on Asteraceae, rarely Apiaceae and Lamiaceae....................**Phanacidini**

Admedian depressions distinct, usually deep and separated more or less widely (Figs 13A, 16C and 16D). Dorsal part of pronotal plate present, lateral margins of pronotal plate at least visible ventrally (Fig 16D). Mesopleuron with different sculpture, usually longitudinally striate (Fig 16F), rarely (*Iraella*) reticulate. R1 reaching or not reaching anterior margin of wing; radial cell closed or open along anterior margin. Gall inducers on Papaveraceae, Lamiaceae, Valerianaceae and Asteraceae....................**11**

**11**. Pronotum dorsomedially relatively shorter, about 1/5 as long as greatest length of outer lateral margin; admedian depressions narrowly separated and more strongly transverse (Figs 13C and 16D). Female antenna with 12 flagellomeres. Mesopleuron striate-reticulate or reticulate (Fig 16E). Gall inducers on *Papaver*....................**Aylacini sensu stricto**

Pronotum dorsomedially longer, 1/4 to 1/3 as long as greatest length of outer lateral margin; admedian depressions usually round or oval, usually more widely separated (Figs 13A and 16C). Female antenna with 10–11 flagellomeres. Mesopleuron usually longitudinally striate (Fig 16F). Gall inducers on Lamiaceae, Asteraceae, Valerianaceae and Fumarioideae (Papaveraceae)....................**Aulacideini**

#### Cynipini

*Cynipsides* Billberg, 1820 *Enumeratio Insectorum in Musaeo Gust*. *Joh*. *Billberg*, p. 101.

Cynipsera Latreille, 1802. Corrected to Cynipidae (erroneous identification (Chalcidoidea) of *Cynips* Linnaeus, 1758, and therefore not available).

*Cynipites* Newman, 1834. *Entomol*. *Mag*., 2: 407.

Habitus, female: Fig 18F and 18G.

Diagnosis: Occiput broadly impressed medially; rising gradually towards vertex (28:2). Pronotum very short dorsomedially, 1/7 or less of the length of outer lateral margin (Fig 13G, 13H and 13I). Admedian depressions of pronotum united medially, forming a transverse impression anteriorly on the pronotum (54:1) (Fig 13G and 13H). Ventral part of pronotal plate small and very reduced medially. Laterodorsal surface of pronotum forming a distinctly inflected broad strip along dorsal margin (63:1). Mesopleuron without a mesopleural impression. 2r vein of fore wing short (130:2) (Fig 12E). Anterior surface of mesocoxa not strongly protruding, peak some distance from base of coxa (120:1). Hypopygium usually with ventral spine distinctly projecting posteriorly (Fig 12F). Cercus well removed from apex of ninth abdominal tergum (156:1).

Biology: Gall inducers on species of *Quercus*, *Castanea*, *Chrysolepis* and *Lithocarpus* (Fagaceae).

Diversity and distribution: 34 genera and about 1000 species. Holarctic, Neotropical, Oriental.

#### Diplolepidini

*Diplolepariae* Latreille, 1802. corrected to Diplolepidae.

*Rhoditini* Hartig, 1840.

(*) The family name Diplolepidae proposed by Latreille, 1802, has priority over the name Cynipidae based on Cynipsides Billberg and Cynipites Newman. However, changing the family name at this point would not be convenient and we follow the suggestion of Nieves-Aldrey [37] of conserving the traditional name for cynipids. Eventually, formal conservation of Cynipidae by application to the International Commission of Zoological Nomenclature might be considered to settle the question once and for all.

Habitus, female: Fig 18A.

Diagnosis: Pronotum short dorsomedially. Pronotal plate not marked (59:2) (Fig 11E). Scutellar foveae faint or absent. Mesopleuron with a broad, crenulate mesopleural impression (166:1) (Fig 11F). Lateral propodeal carinae indistinct (108:1) (Fig 11K). Metanotal trough broad, apically truncate (104:1). 2r of fore wing with a prominent median vein stump projecting anterolaterally. Nucha dorsally short (110:1). Hypopygium plough-shaped (Fig 11G). Ovipositor articulation present as a weak flexion point or a distinct articulation (168:1).

Biology: Gall inducers on *Rosa* spp. (Rosaceae) (233:1).

Diversity and distribution: Two genera, *Diplolepis* and *Liebelia*, and 58 species. Holarctic.

#### Pediaspidini

*Pediaspidini* Ashmead, 1903. *Psyche* (Cambridge Mass.), 10: 147.

*Himalocynipinae* Yoshimoto, 1970. *Can*. *Entomol*., 102: 1583.

Habitus, female: Fig 18D.

Diagnosis: Facial strigae radiating from clypeus distinct but not reaching past 0.6 distance to compound eye (7:2). Sculpture on vertex dorsad compound eye more or less erased (15:1). Ventral area of gena with smooth sculpture, without vertical carinae (227:0). Ventral part of clypeus broadly projecting over mandibles. Female antenna with 12 or more flagellomeres; last flagellomere not wider than the penultimate (Fig 11I). Male antenna without modified F1 (Fig 11H). Dorsolateral margin of pronotal plate not projecting laterad (Fig 11C); admedian depressions of pronotum deep and widely separated (Fig 11C); area posterior to transscutal fissure flat or convex. Subventral impression of pronotum broad and shallow (66:1). Surface sculpture on lateral surface of pronotum (excluding carinae) largely glabrous (not sculptured) (62:1) (Fig 11B). Anterior mesoscutal margin in dorsal view angled laterally, broadly rounded medially (70:1). Scutellar foveae absent; a round, distinctly margined posteromedian scutellar impression present (88:1) (Fig 11A). Mesopleural impression linear and not sculptured (Fig 11B). Profemur not modified. Mesocoxa with a hump present laterobasally (122:1).

Biology: Gall inducer on *Acer* spp. (Sapindaceae) (233:4).

Diversity and distribution: Two genera, *Himalocynips* and *Pediaspis*, with one species each. Palaearctic.

#### Eschatocerini

*Eschatocerini* Ashmead, 1903. *Psyche* (Cambridge Mass.), 10: 147.

Habitus, female: Fig 18B.

Diagnosis: Subocular (malar) furrow present (8:1) (Fig 10B). Toruli situated very high on the head, their inner margins in close contact separated by a strong longitudinal carina (Fig 10B). Maxillary palps three-segmented. Female antenna filiform, with 11 flagellomeres, male antenna without modified flagellomeres; coeloconic sensillae type A on female antenna very large and situated far from distal margin of flagellomere (Fig 10E). Notauli and scutellar foveae absent (84:3) (Fig 10D). Dorso-axillar area large, triangular and situated in same plane as mesoscutellum. Lateral propodeal carina completely absent (Fig 10A). Rs+M and R1 of fore wing inconspicuous. Radial cell with Rs quite separate from anterior wing margin. Basal vein absent. Bulla in R1+Sbc absent (136:0). R1 ending distinctly before reaching anterior margin (132:3) (Fig 10C). Hair fringe along apical margin of wing absent (139:1). Metatarsal claws simple.

Biology: Gall inducer on *Prosopis* spp. and *Acacia* spp. (Fabaceae) (233:2).

Diversity and distribution: A single genus, *Eschatocerus*, with 3 species. South Neotropical.

#### Qwaqwaiini

*Qwaqwaiini* Liljeblad, Nieves-Aldrey & Melika, 2011. *Zootaxa*, 2806: 37.

Habitus, female: Fig 18E.

Diagnosis: Right mandible with two teeth. Ventral margin of clypeus straight. Clypeo-pleurostomal lines ventrally converging (4:1). Occiput with a strong, sharp, distinct carina (176:1) (Fig 12A). Second segment of maxillary palp long (41:1) (Fig 12A). Parascutal carina extending to notaulus. A mesopleural impression present (216:1). Lateral propodeal carina indistinct (180:1). Tarsal claws simple. Radial cell closed along anterior margin, with a short abscissa from R1, before meeting wing margin, forming a narrow costal cell (Fig 12D). Areolet present, large. Cu_1_ and Cu_1a_ not separated by a gap. Third abdominal tergum short, covering only about 1/3 of metasoma. Hypopygium abrupt, without any visibly prolonged ventral spine, with a conspicuous and dense tuft of setae (Fig 12C).

Biology: Gall inducer on *Scolopia* spp. (Salicaceae).

Diversity and distribution: One genus and species *Qwaqwaia scolopiae*. Afrotropical (229:4).

#### Paraulacini

*Paraulacini* Nieves-Aldrey & Liljeblad, 2009. *Zootaxa*, 2200: 5.

Habitus, female: Fig 17G.

Diagnosis: Gena with 5–9 vertical carinae in the ventral region (227:1) (Fig 9G). Genal part of occipital carina present. Ventral part of clypeus not or only slightly projecting over mandibles. Gular sulci free, well separated at hypostoma (19:2). Female antenna with 10 flagellomeres, F10 clavate (Fig 9D). Modified flagellomere of male antenna always F2, F3 or both (Fig 9F). Dorsolateral margin of pronotal plate projecting laterally (Fig 9A). Lateral pronotal carina present (60:0). Scutellar foveae always shallow or indistinct; round, distinctly margined posteromedian scutellar impression absent (Fig 9C). Mesopleural impression present (216:1) (Fig 9B). Profemur with the basal third swollen and carrying a structure of 4–5 rows of sharp, closely spaced and deep costulae (228:1) (Fig 9E).

Biology: inquilines or parasitoids in chalcidoid galls (Pteromalidae) on *Nothofagus* spp. (Nothofagaceae) (230:0).

Diversity and distribution: Includes the genera *Paraulax* and *Cecinothofagus* with 6 species. South Neotropical (229:2); recorded from Chile and Argentina only.

#### Aylacini sensu stricto

*Aylacini* Ashmead, 1903.

*Aulacini* Ashmead, 1903. *Psyche* (Cambridge, Mass.) 10: 147.

*Aylaxini*: Quinlan, 1968. *Trans*. *Entomol*. *Soc*. *London*, 120: 275.

*Aylacinae* Kovalev, 1982. *Tr*. *Zool*. *INST*. *Akad*. *Nauk SSSR*, 110: 85.

*Aulacideini* Fergusson in Gauld & Bolton, 1988: 143.

Incorrect emendation of *Aulacini* Ashmead, 1903.

*Aylacini* Nieves-Aldrey, 1994. *J*. *Hymenop*. *Res*., 3: 177.

The tribe Aylacini sensu lato, which has long been known to be a paraphyletic assemblage of genera, is here restricted to the genera *Aylax*, *Barbotinia and Iraella*, all of which are gall inducers on plant species of the genus *Papaver*. The remaining genera formerly included in Aylacini are transferred to the new tribes Aulacideini, Phanacidini and Diastrophini.

Habitus, female: Fig 17A.

Diagnosis: Ventral part of clypeus usually strongly projecting over mandibles. Projecting part of clypeal margin sinuate or incised (2:1). Transition between dorsomesal margin of eye and surface of face smooth, face not raised (14:1). Prementum and stipes elongate (36:1). Female antenna 14-segmented; male 15-segmented. Pronotum dorsomedially short, less than 0.17 as long as greatest length of outer lateral margin (51:2) (Figs 13C and 16D). Admedian depressions narrowly separated and strongly transverse (Figs 13C and 16D). Mesopleuron striate-reticulate or reticulate (Fig 16E).

Biology: Gall inducers on *Papaver* spp. (Papaveraceae) (233:7).

Diversity and distribution: Three included genera—*Barbotinia*, *Aylax*, *Iraella*—with five described species. Palearctic.

#### Aulacideini

***Aulacideini*** Nieves-Aldrey, Nylander & Ronquist, **new tribe** [urn:lsid:zoobank.org:act: 5C81B532-61D0-4C24-94FC-0DAD3D56A99E]. Type genus: *Aulacidea* Ashmead, 1897.

This tribe is proposed here for a large group of herb galling genera previously included in Aylacini. The species are mostly associated with host plants in the Lamiaceae or Asteraceae. The results of the present study clearly indicate that some of the current genera are unnatural, and that the generic classification of this tribe is in need of revision.

Habitus, female: Fig 17B.

Diagnosis: Sculpture on occiput transversely wrinkled (29:0). Transition between dorsomesal margin of eye and surface of face smooth, face not raised (14:1). Female antenna 12–13-segmented; male 13–14-segmented. Pronotum dorsomedially 1/4 to 1/3 as long as greatest length of outer lateral margin (Figs 13A and 16C); admedian depressions distinct, spherical to oval, and widely separated (Fig 13A). Ventral corner of spiracular incision of pronotum pointed (64:0). Distance between metepimeron and metepisternum intermediate, about as long as width of metepimeron (106:1). Sculpture anteriorly on mesopleuron, below mesopleural triangle, covered with regular, closely set striae (93:1) (Fig 16F). Sculpture of speculum longitudinally, horizontally costate-costulate (91:2) (Fig 16F). Radial cell open or closed along anterior margin. Sternal part of petiolar annulus (ventral margin of petiole) present, slightly projecting (148:1).

Biology: Gall inducers on Asteraceae, Lamiaceae, Valerianaceae and the tribe Fumarioideae (Papaveraceae).

Diversity and Distribution: Included genera: *Antistrophus*, *Aulacidea*, *Cecconia*, *Hedickiana*, *Isocolus*, *Liposthenes*, *Neaylax*, *Panteliella*, *Rhodus*. 75 species. Holarctic.

#### Phanacidini

***Phanacidini*** Nieves-Aldrey, Nylander & Ronquist, **new tribe** [urn:lsid:zoobank.org:act: 41EE46B6-C9FC-4A5E-9269-EED5E9980CEE]. Type genus: *Phanacis* Förster, 1860.

This new tribe is proposed here for a monophyletic group of herb gallers, generally small species, which predominantly induce galls on plants of the family Asteraceae. Many of the species are stem gallers.

Habitus, female: Fig 17F.

Diagnosis: Gular sulci indistinct (20:1). Cardo bent distally some distance from apex, large part visible in posterior view of head (38:0). Male antenna with 14 segments (47:1). Admedian depressions of pronotum indistinct and shallow, sometimes united medially by a transverse linear impression (55:2) (Figs 13B and 16B). Pronotal plate absent. Anterior mesoscutal margin in dorsal view evenly rounded throughout (70:3). Lateral part of mesopectus short and high, ratio of maximum height to maximum width > 1.60 (90:2). Mesopleuron with reticulate or rugulose-striate sculpture (Fig 16A). Sculpture on speculum rugulose (91:1). R1 tubular along basal part of anterior margin of marginal cell; marginal cell partially closed (132:1).

Biology: Gall inducers on several genera of Asteraceae, rarely on *Phlomis* (Lamiaceae) and *Eryngium* (Apiaceae) (233:5).

Diversity and Distribution: The tribe includes the genera *Asiocynips*, *Diakontschukia*, *Phanacis*, *Timaspis*, *Zerovia* with about 40 species in total. Palearctic, one species South Afrotropical (possibly introduced). Introduced in South America and Australia.

#### Diastrophini

***Diastrophini*** Nieves-Aldrey, Nylander & Ronquist, **new tribe** [urn:lsid:zoobank.org:act: 287E967F-088D-4CC9-8ED1-ADD2F4EB7A66]. Type genus: *Diastrophus* Hartig, 1840.

This tribe is proposed for the apparently monophyletic group that includes the gallers *Diastrophus* and *Xestophanes*, formerly included in the tribe Aylacini, and the inquilines *Periclistus* and *Synophromorpha*, formerly included in the Synergini. Both the gallers and the inquilines are associated with host plants in the family Rosaceae.

Habitus, female: Fig 17C and 17D.

Diagnosis: Clypeo-pleurostomal lines absent (3:1). Gular sulci indistinct (21:1). Female antenna 12-segmented. Longitudinal ridge on F1 of male antenna present, extending part of length of F1 (49:1). Pronotum long medially; pronotal plate distinct, lateral margins marked entirely, almost reaching anterior margin of pronotum (59:0) (Figs 13D, 15B and 15E). Mesoscutum and mesopleuron without sculpture (*Diastrophus* and *Xestophanes*) (Fig 15D) or sculptured (*Periclistus* and *Synophromorpha*). Metatarsal claws toothed; apex strongly bent, base expanded to a lobe or tooth (124:1) (Fig 15C). Claw with long subapical seta (125:1). Abdominal terga 3–8 either free in the two sexes (*Diastrophus*) or 3+4 fused in females; free in males (*Xestophanes*, *Periclistus* and *Synophromorpha*). Sternal part of petiolar annulus (ventral marginal flange of petiole) present, distinctly projecting (148:2).

Biology: Gall inducers in galls on *Rubus* spp. and *Potentilla* spp. (Rosaceae), rarely on *Smilax* (Smilacaceae), and inquilines in cynipid galls on *Rubus* spp. (*Synophromorpha*) and *Rosa* spp. (Rosaceae) (*Periclistus*) (233:1).

Diversity and distribution: The tribe includes the genera *Diastrophus*, *Xestophanes*, *Synophromorpha* and *Periclistus* (last two genera transferred from Synergini). 32 species. Holarctic and Neotropical (one species).

#### Ceroptresini

***Ceroptresini*** Nieves-Aldrey, Nylander & Ronquist, **new tribe** [urn:lsid:zoobank.org:act: EDCA3533-F989-47B4-8B69-94F8D3879A89]. Type genus: *Ceroptres* Hartig, 1840.

The genus *Ceroptres* formerly included in the Synergini is here raised to its own tribe. The species belonging to the genus appear to form a monophyletic group, but there is little evidence currently grouping them with any of the other cynipid tribes.

Habitus, female: Fig 17E.

Diagnosis: Head in frontal view with two distinctly raised vertical carinae issuing from ventral margin of antennal socket, at least present close to antennal socket (Fig 14A). Ventral margin of clypeus straight, not projecting over mandibles. Clypeo-pleurostomal lines absent (3:1). Upper face, vertex and mesopleuron almost smooth. Distance between occipital and oral foramina longer than height of occipital foramen (22:1). Female antenna with 10 flagellomeres. Pronotum large medially. Pronotal plate complete; lateral margins reaching anterior margin of mesoscutum and pronotum (Fig 14B). Lateral part of mesopectus long and low, ratio of maximum height to maximum width < 1.12 (90:0). Lateral propodeal carina broad, flattened above (109:1) Radial (marginal) cell closed. Metatarsal claws toothed (124:1). Claw with long subapical seta (125:1). Third abdominal tergum small and free (not fused with the fourth) in the two sexes; with a dense hair patch anterolaterally (Fig 14C). Third valvula of ovipositor long, projecting beyond apex of the ninth abdominal tergum more than one width of a third valvula (160:1).

Biology: Associated with cryptic cynipid galls on *Quercus* (235:0). They are probably inquilines but there are no detailed observations of their life history. We observed a female of *Ceroptres* sp. laying egg into an almost mature gall of *Callirhytis clavula* on white oak (*Quercus alba*) in Pennsylvania, USA (Liu, unpublished data).

Diversity and Distribution: Includes the single genus *Ceroptres* with about 21 species. Holarctic.

#### Synergini sensu stricto

*Synergini* Ashmead, 1896

*Synerginae* Ashmead, 1896. *Trans*. *Am*. *Entomol*. *Soc*., 23: 186

The tribe Synergini, which used to include all the familiar Northern Hemisphere inquilines, is narrowed down here to include only the inquilines associated with Fagaceae, plus the afrotropical *Rhoophilus*, which is an inquiline in galls on *Rhus*. There is strong molecular evidence for the monophyly of this clade. The genus *Ceroptres* is moved to its own tribe, while *Periclistus* and *Synophromorpha* are transferred to the tribe Diastrophini.

Habitus, female: Fig 18C.

Diagnosis: Head and/or mesosoma usually strongly sculptured (Fig 14D). Ventral clypeal margin not projecting over mandibles. Ventral part of hypostoma distinctly raised, projecting from cranial margin (18:1). Distance between occipital and oral foramina long (22:1). Dorsal part of pronotal plate incomplete; lateral margins of dorsal part of posterior part of pronotal plate only marked ventrally (59:1) (Fig 13E and 13F). Mesoscutum with transverse carinae (76:0). Posterodorsal margin of axillula not marked. Speculum horizontally costate-costulate (91:2). Metatarsal claws toothed (124:1). Nucha and abdominal tergum 2 usually ring-shaped and longitudinally sulcate (198:1) (Fig 15A). Abdominal terga 3+4 fused into one large syntergum in both sexes, occupying nearly entire metasoma (144:1) (Fig 15A).

Biology: Inquilines (230:1) (rarely true gall inducers) in cynipid galls on several Fagaceae genera, mainly *Quercus*. One genus (*Rhoophilus*) is an inquiline in Cecidosidae (Lepidoptera) galls on *Rhus* spp. (Anacardiaceae); it may be the sister group of the remaining lineages in the group.

Diversity and distribution: Includes the genera *Agastoroxenia*, *Lithonecrus*, *Lithosaphonecrus*, *Rhoophilus*, *Saphonecrus*, *Synophrus*, *Synergus*, *Ufo*. About 180 species. Holarctic, Neotropical, Oriental. One new genus and species found recently in the Oceanian region [60].

## Supporting Information

S1 Appendix Morphological and biological characters. Definitions of the morphological characters and life-history traits used in the analyses.
(DOCX)

S1 Commands MrBayes command blocks. Command blocks for all analyses described in the paper.
(NEX)

S1 Dataset Combined data. Nexus file for MrBayes runs.
(NEX)

S2 Dataset Molecular data. Nexus file for MrBayes runs.
(NEX)

S3 Dataset Morphological and biological data. Nexus file for MrBayes runs.
(NEX)

S1 Fig Gene tree for COI. Results from Bayesian MCMC analysis, with posterior clade probabilities.
(PDF)

S2 Fig Gene tree for 28S. Results from Bayesian MCMC analysis, with posterior clade probabilities.
(PDF)

S3 Fig Gene tree for LWRh. Results from Bayesian MCMC analysis, with posterior clade probabilities.
(PDF)

S4 Fig Gene tree for EF1aF1. Results from Bayesian MCMC analysis, with posterior clade probabilities.
(PDF)

S5 Fig Gene tree for EF1aF2. Results from Bayesian MCMC analysis, with posterior clade probabilities.
(PDF)

S6 Fig Tree based on morphological data, without life-history traits. Results from Bayesian MCMC analysis, with posterior clade probabilities.
(PDF)

S7 Fig Tree based on morphology and molecules, without life-history traits. Results from Bayesian MCMC analysis, with posterior clade probabilities.
(PDF)

S8 Fig Tree based on morphology and molecules, with morphological characters unordered. Results from Bayesian MCMC analysis, with posterior clade probabilities.
(PDF)

S9 Fig Tree based on combined data, with incompletely coded sequence data excluded. Results from Bayesian MCMC analysis, with posterior clade probabilities.
(PDF)

S10 Fig Tree based on molecular data, with incompletely coded sequence data excluded. Results from Bayesian MCMC analysis, with posterior clade probabilities.
(PDF)

S1 Table Material examined. Detailed information on the taxa and sequences used in the analyses, including sequence accession numbers.
(DOC)

S2 Table Inferred ancestral traits. Inferred ancestral traits of Cynipidae and the twelve cynipid tribes recognized in the paper. For the twelve tribes, we also give the distinctness and uniqueness index of each character state.
(XLSX)
