# Supplementary material for: Phylogeny, Evolution and Classification of Gall Wasps: The Plot Thickens
Source: PLoS One. 2015 May 20;10(5):e0123301. doi: 10.1371/journal.pone.0123301 (PMC4439057; doi:10.1371/journal.pone.0123301)
Supplement: S1 Appendix — Definitions of the morphological characters and life-history traits used in the analyses. (DOCX) [file pone.0123301.s001.docx]

**S1 Appendix**

List of morphological and biological characters used in the analysis. The following abbreviations are used for papers from which characters were coded: LR98 = [28], NL96 = [43], R94 = [27], R95 = [7], R99 = [14], RR00 = [44] (reference numbers refer to main paper). At the end of the list, we comment on the characters appearing in those papers and not used in the present study. Throughout the character list, we precede a reference to a previously described character by ‘modifed from’ if the character has been extended or modified, or with a tilde (‘~’) if the character definition is similar but not identical to the one used here. In all other cases, the character definition used here is identical to the original reference.

We have explicitly commented on all cases where the coding found in the original paper was complemented by us or by using information from previously published papers. In LR98, ibaliids and liopterids were coded for the groundplan characters of their families, but here we use coding of the exemplar taxa throughout. The complete matrix used by us is available from TreeBase (reference number: 15832).

*Morphological characters*

Characters 1–164 are taken from LR98 and are listed first, followed by characters from other sources. Unless explicitly stated otherwise, characters refer to females. Abbreviations: FX = flagellomeres X; Xtg = abdominal tergum X.

1. (LR98:1) *Shape of ventral part of clypeus*: (0) rounded, broadly projecting over mandibles (Fig. R94:8a); (1) not projecting from cranial margin or slightly and narrowly projecting medially (Fig. R94:7).

2. (LR98:2) *Shape of projecting ventral clypeal margin*: (0) straight or rounded; (1) with a median incision (Fig. R94:8).

1. 3. (R94:2, LR98:3) *Clypeo-pleurostomal lines*: (0) present, at least visible by different sculpture (Fig. R94: 8); (1) absent (Fig. R94: 7).

4. (LR98:4) *Direction of clypeo-pleurostomal lines*: (0) ventrally diverging  (Fig. R94:8); (1) ventrally converging (Fig. R94:7).

5. (R94:3, LR98:5) *Epistomal sulcus*: (0) present, at least marked by a distinct change in curvature of lower face (Fig. R94:8); (1) absent (Fig. R94:7).

6. (LR98:6) *Relative distance between anterior tentorial pits*: (0) long, pits  closer to ventral clypeal margin than to each other (Fig. R94:7); (1) short, pits closer to each other than to ventral clypeal margin (Fig. LR98:7a).

7. (LR98:7, modified from R94:4 & R94:5) *Facial strigae radiating from clypeus*: (0) laterally reaching or almost reaching compound eye (Fig. R94:7); (1) laterally reaching past 0.7 distance to compound eye; (2) distinct but not reaching past 0.6 distance to compound eye (Fig. LR98:7a); (3) entirely absent or only a few strigae indicated close to clypeus (Fig. R94:8). Ordered.

1. 8. (R94:6, LR98:8, RR00:3) *Subocular (malar) furrow (impression)*: (0) absent (Fig. R94:7); (1) present (Fig. R94:8). Coding complemented.

9. (LR98:9) *Raised vertical carina from ventral margin of antennal socket*: (0) absent; (1) present, at least close to antennal  socket.

1. 10. (R94:12, LR98:10) *Shape of surface of upper face*: (0) flat (Fig. R94:7); (1) slightly raised medially; (2) distinctly raised medially (Fig. R94:8). Ordered.

11. (R94:7, LR98:11) *Shape of antennal rim*: (0) distinctly widened lateroventrally (Fig. R94:7); (1) not distinctly widened lateroventrally (Fig. R94:8).

12. (LR98:12, modified from R94:9) *Size of antennal socket*: (0) small, ratio of  max. width of head to max. width of antennal socket excluding antennal rim > 9.0 (Fig. R94:7); (1) large, ratio < 8.5 (Fig. R94:8).

1. 13. (R94:10, LR98:13) Orbits: (0) flat, not impressed (Fig. R94:7); (1) slightly but distinctly impressed (Fig. R94:8).

14. (LR98:14) *Transition between dorsomesal margin of eye and surface of face* : (0) smooth, surface of face slightly raised just before meeting margin of eye; (1) smooth, face not raised; (2) abrupt, distinct angle between face and eye. Unordered.

15. (Modified from R94:13) *Sculpture on vertex dorsad compound eye*: (0) regular and non-parallel (polygonal); (1) more or less erased; (2) punctate. Unordered.

16. (Modified from R94:13) *Regular sculpture on vertex dorsad compound eye*: (0) concave (alveolate-reticulate); (1) flat (coriarious); (2) convex (acinose-colliculate). Ordered.

1. 17. (Modified from R16) *Median hairy strip of gula*: (0) broad, at least in upper half; (1) narrow throughout (Fig. R94:9); (2) reduced or absent (Fig. R94:10). Unordered.

18. (R94:17, LR98:18) Shape of ventral part of hypostoma: (0) not or only slightly projecting from cranial margin, only slightly raised (Fig. R94:10); (1) distinctly projecting from cranial margin, distinctly raised (Fig. R94:9).

19. (LR98:19, modified from R94:15) *Position of gular ridges*: (0) united well before reaching hypostomata (Fig. R94:9); (1) free, but meeting at hypostomata; (2) free, well separated at hypostomata (Fig. R94:10). Ordered.

20. (LR98:20) *Appearance of gular sulci*: (0) distinctly marked; (1) indistinct; (2) absent, at most barely indicated. Ordered.

21. (LR98:21) *Distinctness of gular ridges*: (0) distinctly raised; (1) reduced, not ridge-like.

22. (R94:14, LR98:22) Distance between occipital and oral foramina: (0) shorter than height of occipital foramen including postoccipital rim (Fig. R94:10); (1) longer than height of occipital foramen (Fig. R94:9).

 23. (LR98:23) *Position of posterior tentorial pits*: (0) high, dorsal margin of pits positioned higher than ventral postoccipital rim of occipital foramen (Fig. R94:ß9); (1) low, dorsal margin of pits positioned lower.

24. (LR98:24) *Shape of posterior tentorial pits*: (0) more or less rounded (Fig. LR98:7b); (1) slitlike (Fig. R94:10).

25. (R94:18, LR98:25) *Depression laterad hypostoma, close to ventral margin of cranium*: (0) absent (Fig. R94:10); (1) present (Fig. R94:9).

26. *Ridge from hypostomata close to ventral margin of cranium* *reaching laterad*: (0) absent; (1) present (Fig. R94:9).

 27. (R95:5, LR98:27) *Shape of posterior surface of cranium*: (0) distinctly impressed below occipital foramen, postocciput and gula set in cavity (Fig. R95:2a); (1) almost flat, not impressed below occipital foramen, postocciput and gula not set in cavity (Fig. R95:2b). Coding complemented.

28. (LR98:28) *Shape of occiput medially*: (0) flat or only slightly impressed close to postocciput (Fig. LR98:7c); (1) distinctly impressed close to occiput, impression separated by a sharp edge from rest of occiput (Fig. LR98:7d); (2) broadly impressed, rising gradually towards vertex (Fig. LR98:7e). Unordered.

29. (LR98:29) *Sculpture on occiput*: (0) transversely wrinkled; (1) not wrinkled.

30. (LR98:30) *Shape of odontoidea*: (0) narrow and more or less pointed laterally, abruptly broadened close to mesal margin (Fig. LR98:7b); (1) broader and more rounded laterally, gradually broadened towards mesal margin (Figs R94:9, R4:10).

31. (R94:19, LR98:31) *Number of teeth with corresponding internal rods on right mandible*: (0) three (Fig. R94:11); (1) two (Fig. R94:12).

32. (R94:20, LR98:32) *Basal swelling on anterior side of mandible*: (0) small or indistinct (Fig. R94:12); (1) large, conspicuous (Fig. R94:11).

33 (R94:21, LR98:33) *Size of oval window*: (0) large (Fig. R94:13); (1) small or almost absent (Fig. R94:14).

34. (R94:22, LR98:34) *Shape of dorsal margin of upper tooth on left mandible*: (0) evenly rounded, no extra tooth indicated (Fig. R94:13); (1) produced into an extra tooth (Fig. R94:14).

35. (R94:23, LR98:35) *Posterior region of ventral surface of mandible*: (0) horizontal, set off from posterior surface of mandible by a distinct carina (Fig. R94:13); (1) oblique, gradually continued in posterior surface of mandible (Fig. R94:14).

36. (LR98:36) *Shape of prementum and stipes*: (0) short (Fig. R94:10); (1) elongate (Fig. LR98:7b).

37. (R94:24, LR98:37) *Longitudinal, mesal carina on posterior surface of stipes*: (0) present (Fig. R94:9); (1) absent (Fig. R94:10).

38. (LR98:38, modified from R94:25) *Shape of cardo*: (0) bent distally some distance from apex, large part visible in posterior view of head. (Fig. R94:9); (1) bent distally close to apex, only small part visible posteriorly; (2) straight, not bent distally, not or almost not visible posteriorly (Fig. R94:10). Ordered.

39. (R94:26, LR98:39) *Shape of apical peg on last segment of maxillary and labial palpus*: (0) long and narrow (Figs R94:15, R94:17); (1) short and broad, situated apically (Figs R94:16, R94:18).

40. (R94:28, LR98:40) *Articulation between fourth and fifth segments of maxillary palpus*: (0) normal, free articulation (Fig. R94:15); (1) fifth segment rigidly inserted into fourth (Fig. R94:16); (2) fifth and fourth segments fused. Ordered.

41. (LR98:41, modified from R94:30) *Length of second segment of maxillary* *palp*: (0) short, ratio of length of second segment to length of third to fifth segment combined < 0.50 (Fig. R15); (1) long, ratio > 0.54 (Fig. R16).

42. (R94:31, LR98:42) *Shape of first segment of maxillary palpus*: (0) subrectangular, broader than long (Fig. R94:16); (1) triangular, longer than broad (Fig. R94:15).

43. (R94:32, LR98:43) *Number of segments of labial palpus*: (0) three normal segments (Figs R94:17, R94:18); (1) three segments, second strongly reduced in size; (2) two segments. Ordered.

44. (R94:33, LR98:44) *Shape of first segment of labial palpus*: (0) long, gradually tapering towards base (Fig. R94:16); (1) short, abruptly tapering towards base (Fig. R94:17).

45. (R94:34, LR98:45) *Number of completely separated flagellomeres of female antenna*: (0) 10; (1) 11; (2) 12; (3) 13. Ordered.

46. (R94:35, LR98:46) *Length of F1 of female antenna*: (0) short, ratio of length of F1 to length of F2 ≤ 1.00; (1) long, ratio ≥ 1.10.

47. (LR98:47, modified from R94:36) *Number of flagellomeres of male antenna*: (0) 13 or more; (1) 12; (2) 11. Ordered *Paramblynotus* coded for actual state (1) rather than for likely ground-plan state of Liopteridae.

1. 48. (LR98:48, ~RR00:5, ~R95:10; modified from R94:38) *Modified part of male F1*: (0) present, clearly set off as a separate flattened or bare surface (Fig. R94:19); (1) absent or at most indicated basally (Fig. LR98:7f). The *Ibalia* and liopterid exemplars of this study coded for actual state (0) rather than the likely ground-plan state of their families.
2. 49. (R94:39, LR98:49) *Longitudinal ridge on F1 of male antenna*: (0) absent (Fig. R94:20); (1) present, extending part of length of F1; (2) present, extending entire length of F1 (Fig. R94:19). Unordered.
3. 50. (LR98:50) *Shape of F1 of male antenna*: (0) short, ratio of length of F1 to width of F2 < 3.5 (Fig. R94:19); (1) long, ratio > 4.2 (Fig. R94:7f).
4. 51. (LR98:51, modified from R94:42) *Shape of pronotum*: (0) long medially, ratio of median distance between dorsal and ventral margins to shortest lateral distance between anterior margin and anteroventral corner of mesopleural triangle ≥ 0.43 (Fig. R94:26); (1) intermediate, ratio 0.35–0.34 (Fig. LR98:8b); (2) short, ratio 0.29–0.22 (Fig. R94:25); (3) very short, ratio ≤ 0.17 (Fig. R94:27). Ordered.
5. 52. (LR98:52) *Shape of anteroventral margin of pronotum in anterodorsal view*:  (0) evenly rounded (Fig. R94:29); (1) with a median incision  (Fig. R94:30).

53. (R95:14, LR98:53) *Shape of anterior flange of pronotum*: (0) long, horizontal (Figs R95:2a, R95:5, R95:7a); (1) short, oblique (Figs R95:2b, R95:6, R95:7b). Coding complemented.

54. (R94:40, LR98:54) *Admedian depressions of pronotum*: (0) separated medially (Fig. R94:29); (1) united medially, forming a transverse impression anteriorly on the pronotum (Fig. R94:30).

55. (LR98:55) *Shape of admedian depressions*: (0) round (Fig. LR98:8b); (1) oval  (Fig. R94:29); (2) linear (Fig. LR98:9a, R94:25). Ordered.

56. (R94:41, LR98:56) *Shape of dorsal pronotal margin medially*: (a) sharp, with a distinct dorsal edge (Fig. R94:29); (1) blunt, rounded dorsally (Fig. R94:30).

57. (R95:20, LR98:57) *Dorsal pronotal area (area behind pronotal crest)*: (0) present and exposed, at least marked by different sculpture (Fig. R95:7a); (1) absent or hidden by the mesoscutum (Fig. R95:7b). Coding complemented; *Parnips* and *Plectocynips* coded as having state 1 following R95 contra LR98.

58. (LR98:58) *Sculpture on pronotal plate*: (0) at least partly glabrous-glabrate  and shining; (1) coriarious and dull.

59. (R94:43, LR98:59) *Lateral margin of posterior part of pronotal plate*: (0) marked entirely (Fig. R94:26); (1) marked only ventrally (Fig. R94:27); (2) not marked (Fig. R94:25). Ordered.

60. (R94:44, LR98:60) *Lateral pronotal carina*: (0) present (R94:25); (1) absent (Figs R94:26, R94:27). Coding complemented.

61. (LR98:61, modified from R94:45) *Ridges on lateral surface of pronotum*:  (0) lacking (Fig. R94:26); (1) some irregular, horizontal costulae posteriorly in lower half (Figs R9425, R94:27); (2) many regular, radiating costulae dorsally and posteriorly. Unordered.

62. (LR98:62) *Surface sculpture on lateral surface of pronotum (excluding ridges)*: (0) at least superficially sculptured; (1) largely glabrous.

63. (LR98:63, modified from R94:51) *Shape of laterodorsal surface of pronotum*: (0) more or less vertical (Fig. R94:26); (1) broad strip along dorsal margin distinctly inflected (Fig. R94:27); (2) gradually curved inwards dorsally, particularly subposteriorly (Fig. LR98:8a). Unordered.

64. (LR98:64) *Shape of ventral corner of spiracular incision of pronotum*: (0) pointed (Fig. LR98:8a); (b) rounded (Fig. R94:25).

65. (R94:46, LR98:65) *Shape of lateroventral margin of pronotum*: (0) distinctly concave (Fig. R94:26); (1) straight or very slightly concave (Figs R94:25, R94:27).

66. (LR98:66) *Shape of subventral impression of pronotum*: (0) narrow and  more or less distinct (Figs R94:25–R94:27); (1) broad and shallow (Fig. LR98:8b).

67. (R94:48, LR98:67) *Position of profurcal pit*: (0) anterior to middle of furcasternum (Fig. R94:23); (1) at or behind middle of furcasternum (Fig. R94:24).

68. (R94:49, LR98:67) *Shape of profurcal pit*: (0) rounded, small (Fig. R94:23); (1) transverse, large (Fig. R94:24).

1. 69. (LR98:69) *Lateral profile of anterior part of mesoscutum*: (0) not recurved, anteriormost part same as anteroventral margin (Figs R94:26, R94:27); (1) recurved, anteriormost part dorsad anteroventral margin.
2. 70. (LR98:70) *Shape of anterior mesoscutal margin in dorsal view*: (0) angled laterally, narrowly rounded medially (Fig. R94:29); (1) angled laterally, broadly rounded medially (Fig. R94:30); (2) angled laterally, truncate medially (Fig. LR98:9b); (3) evenly rounded throughout (Fig. LR98:9a). Unordered.
3. 71. (LR98:71) *Incision in anterior margin of mesoscutum at anterior end of anteroadmedian signum*: (0) absent or indistinct (Fig. R94:28); (1) present, distinct (Fig. R94:29).
4. 72. (R94:50, LR98:72) *Impression mesad parascutal carina*: (0) anteriorly ending just in front of tegula (Fig. R94:29); (1) anteriorly continuing to anterior end of notaulus (Fig. R94:30).
5. 73. (LR98:73, modified from R94:51) *Relation between anterolateral mesoscutal*  *margin and dorsal pronotal margin*: (0) mesoscutal margin not projecting over pronotum (Fig. R94:26); (1) mesoscutal margin projecting over pronotum (Fig. R94:27).
6. 74. (LR98:74) *Shape of mesoscutum midlaterally, in cross section*: (0) slightly rounded (Figs R94:26, R94:27); (1) distinctly rounded; (2) conspicuously rounded (Fig. LR98:8a). Ordered.
7. 75. (LR98:75, modified from R94:52, ~R99:2, ~RR00:7) Surface sculpture of mesoscutum: (0) entirely glabrous; (1) partly glabrous; (2) entirely dull. Ordered. Note that this character was not described properly in LR98.
8. 76. (R94:53, LR98:76) Transverse ridges on mesoscutum: (0) present (Fig. R94:29); (1) absent (Fig. R94:30).
9. 77. (R94:54, LR98:77) Pubescence on mesoscutum: (0) about as dense as pubescence laterally on pronotum; (1) distinctly less dense; (2) hairs almost absent. Ordered.
10. 78. (R94:55, LR98:78) Lateral bar: (0) resent (Figs R94:26, R94:27); (1) absent.
11. 79. (R94:56, LR98:79) Posterodorsal margin of axillula: (0) not marked (Fig. R94:27); (1) distinctly marked (Fig. R94:26).
12. 80. (LR98:80) *Shape of posterior part of axillular surface*: (0) shallowly impressed (Fig. R94:26); (1) deeply impressed (Fig. LR98:8a).
13. 81. (LR98:81) *Axillar carina separating lateral axillar area from dorsal axillar area*: (0) more or less distinct (Figs R94:26, R94:27); (1) indistinct or absent (Fig. LR98:8c).
14. 82. (R94:57, LR98:82) *Shape of subaxillular bar*: (0) broad, vertical, evenly continuing posteriorly in shining strip of mesoscutellum (Fig. R94:26); (1) narrow, horizontal, rapidly expanding posteriorly in shining strip of mesoscutellum (Fig. R94:27).
15. 83. (LR98:83) *Posterodorsal part of shining strip*: (0) without a dorsal projection; (1) with a dorsal projection (Figs R94:26 and R94:27).
16. 84. (LR98:84) *Notauli*: (0) percurrent and distinct (Figs R94:29, R94:30); (1) percurrent or almost percurrent, but anterior half indistinct (Fig. LR98:9b); (2) present posteriorly, but absent in anterior half (Fig. LR98:9a); (3) entirely absent. Ordered.
17. 85. (LR98:85) *Median mesoscutal impression*: (0) present, extending some distance from posterior margin of mesoscutum (Fig. LR98:9c); (1) present only as a slight impression at posterior margin of mesoscutum (Fig. R94:30); (2) absent. Ordered.
18. 86. (LR98:86) *Scutellar foveae*: (0) present, at least as transverse furrows (Fig. LR98:9a); (1) strongly reduced or absent.
19. 87. (LR98:87) *Sculpture in scutellar foveae*: (0) glabrous to glabrate, except occasionally for some rugosity, without distinct microsculpture; (1) finely coriarious.
20. 88. (LR98:88) *Round, distinctly margined posteromedian scutellar impression*: (0) absent (Fig. LR98:9a); (1) present (Fig. LR98:9c).

89. (R95:32, LR98:89) *Shape of ventral part of mesopectus and insertion of mesocoxae*: (0) mesopectus protruding ventrally, mesocoxae directed obliquely backwards, inserted on separate, oblique posterior area (Fig. R95:5); (1) mesopectus not protruding ventrally, mesocoxae directed downwards, not inserted on separate, oblique posterior area (Fig. R95:6). Coding complemented.

 90. (LR98:90) *Shape of lateral part of mesopectus*: (0) long and low, ratio of maximum height to maximum width ≤ 1.12; (1) intermediate, ratio 1.30–1.51 (Figs R94:26, R94:27); (2) short and high, ratio ≥ 1.60 (Fig. LR98:8c). Ordered.

91. (R94:58, LR98:91) *Sculpture on speculum*: (0) glabrous with or without punctures (Fig. 94:27); (1) rugulose; (2) longitudinally, horizontally costate-costulate (Fig. R94:26). Unordered.

92. (LR98:92) *Direction of regular costulae-strigae on speculum*: (0) horizontal or directed obliquely upwards posteriorly (Fig. R94:26); (1) directed obliquely downwards posteriorly.

 93. (LR98:93) *Sculpture anteriorly on mesopleuron, below mesopleural triangle*: (0) without regular sculpture (Fig. R94:27); (1) covered with regular, closely set striae, occasionally these striae only indicated (Fig. R94:26); (2) covered with regular, reticulate sculpture. Unordered.

 94. (LR98:94) *Line marking ventral border of mesopleural triangle*: (0) clearly set off (Figs R94:26, R94:27); (1) diffuse, no clear border (Fig. LR98:8c). *Paramblynotus* coded for actual state (0) rather than the likely ground-plan state of Liopteridae.

95. (LR98:95) *Posterior subalar pit*: (0) large, deep (Fig. LR98:8b); (1) small, shallow (Fig. LR98:8c).

96. (LR98:96) *Sculpture on lower half of mesopleural triangle*: (0) smooth or irregular (Fig. R94:27); (1) strigate (Fig. R94:26).

97. (LR98:97) *Ventral surface of mesopectus*: (0) only slightly bulging ventrad medially (Figs R94:26, R94:27); (1) prominently bulging ventrad medially (Figs. LR98:8b,c).

98. (R94:60, LR98:98) *Shape of acetabular carina medially*: (0) absent, not marked (Fig. R94:25); (1) distinct, only slightly raised; (2) distinct, strongly raised (Fig. R94:23). Ordered.

99. (R94:62, LR98:99) *Longitudinal carina from mesocoxal foramen towards acetabular carina, laterally delimiting mesosubpleuron*: (0) absent (Fig. R94:24); (1) present (Fig. R94:23).

100. (R94:63, LR98:100) *Shape of rim surrounding mesocoxal foramen*: (0) narrow throughout or slightly expanded posteriorly, ratio of posterior width to anterior width < 2.0 (Fig. R94:24); (1) distinctly expanded posteriorly, ratio > 2.0 (Fig. R94:23).

101. (R94:64, LR98:101) *Position of mesocoxal foramen*: (0) removed from posterior margin of mesosubpleuron, ratio of distance between posterior margin of mesocoxal foramen and posterior margin of mesosubpleuron to longitudinal width of mesocoxal foramen > 0.32 (Fig. R94:23); (1) close to posterior margin of mesosubpleuron, ratio < 0.32 (Fig. R94:24).

1. 102. (R94:65, LR98:102) *Shape of metascutellum*: (0) subrectangular (Fig. R94:22); (1) distinctly constricted medially (Fig. R94:21).
2. 103. (R94:66, LR98:103) *Sculpture on bar ventral to metanotal troug*h: (0) at least partly smooth or almost smooth (Fig. R94:26); (1) entirely covered with irregular sculpture (Fig. R94:27).

104. (LR98:104, modified from R94:67) *Shape of metanotal trough*: (0) narrow, apically rounded (Fig. R94:27); (1) broad, apically truncate (Fig. LR98:8b).

105. (R94:68, LR98:105) *Position of anterior end of metapleural sulcus*: (0) low, ratio of distance between upper metapectal margin and anterior end of metapleural sulcus to distance between anterior end of metapleural sulcus and anteroventral metepisternal margin > 0.70 (Figs. R94:27, R94:28); (1) high, ratio < 0.65 (Fig. R94:26).

106. (LR98:106) *Distance between metepimeron and metepisternum*: (0) short, distinctly shorter than width of metepimeron (Fig. R94:26); (1) intermediate, about as long as width of metepimeron; (2) long, much longer than width of metepimeron (Fig. LR98:8a). Ordered.

1. 107. (LR98:107) *Carina from ventral margin of calyptra to metapleural*  *sulcus*: (0) present (Fig. LR98:8b); (1) absent (Fig. LR98:8a).
2. 108. (LR98:108) *Lateral propodeal carina*: (0) present; (1) absent.
3. 109. (LR98:109) *Shape of lateral propodeal carina*: (0) narrow, not flattened above; (1) broad, flattened above.

110. (R94:69, LR98:110)  Length of nucha dorsally: (0) long, ratio of length of nucha to width of metacoxa > 0.29 (Figs R94:26, R94:27); (1) short, ratio < 0.26 (Fig. R94:28).

111. (LR98:111) *Posterodorsal edge of nucha, in lateral view*: (0) rounded (Figs R94:26, R94:28); (1) angled (Fig. R94:27).

112. (R94:70, LR98:112) *Position of metacoxal foramen*: (0) removed from anterior margin of metasubpleuron, ratio of shortest distance between anterior margin of subpleuron and metacoxal foramen to longitudinal length of metacoxal foramen > 0.29 (Fig. R94:24); (1) closer to anterior margin, ratio < 0.22 (Fig. R94:23).

113. (R94:72, LR98:113) *Blunt projection from anterior margin of metasubpleuron mesad metacoxal foramen*: (0) absent (Fig. R94:24); (1) present (Fig. R94:23).

114. (R94:73, LR98:114) Position of metafurcal pit: (0) anteriorly situated, ratio of distance between anterior margin of metasubpleuron and metafurcal pit to distance between metafurcal pit and anterior margin of rim surrounding petiolar foramen ≤ 1.5 (Fig. R94:24); (1) more posteriorly situated, ratio > 1.6 (Fig. R94:23).

1. 115. (LR98:115) *Triangle formed by petiolar and metacoxal foramina*: (0) narrow, ratio of distance between centres of metacoxal foramina to longitudinal distance between anterior margin of metacoxal foramen and anterior margin of petiolar foramen < 2.6 (Fig. R94:23); (1) broad, ratio > 3.0 (Fig. R94:24).
2. 116. (LR98:116, modified from R94:74) *Position of petiolar foramen*: (0) anteriorly situated, ratio of distance between anterior margin of metasubpleuron and anterior margin of petiolar foramen to length of petiolar foramen, in ventral view < 1.8; (1) posteriorly situated, ratio > 2.0.

117. (R94:75, LR98:117) *Carina from posteroventral corner of procoxa towards basal area*: (0) absent (Fig. R94:36); (1) present (Fig. R94:35).

118. (R94:76, LR98:118) *Anterior, lateral crest of procoxa*: (0) present (Fig. R94:35); (1) absent (Fig. R94:36).

119. (R94:77, LR98:119) *Shape and position of annular girdle of procoxa*: (0) posteriorly only just touching peripheral margin of basal area (Fig. R94:35); (1) posteriorly following peripheral margin of basal area for some distance (Fig. R94:36).

120. (R94:79, LR98:120) *Shape of anterior surface of mesocoxa*: (0) strongly protruding, peak close to base of coxa (Fig. R94:33); (1) less strongly protruding, peak farther from base of coxa (Fig. R94:34).

121. (R94:80, LR98:121) *Shape of annular girdle of mesocoxa*: (0) distinctly set off from rest of coxa (Fig. R94:31); (1) posteriorly continuous with posterior surface of coxa (Fig. R94:32).

122. (LR98:122) *Hump laterobasally on mesocoxa*: (0) absent; (1)  present.

123. (LR98:123, modified from R94:78] *Shape of mesofemur*: (0) approximately same  width throughout, only slightly widened subbasally (Fig. R94:32); (1) distinctly and abruptly widened subbasally (Fig. R94:31).

124. (R94:82, LR98:124) *Shape of metatarsal claw*: (0) apex slightly bent, base not expanded (Fig. R94:40); (1) apex strongly bent, base expanded to a lobe or tooth (Fig. R94:39).

125. (R94:83, LR98:125) Long subapical seta on claw: (0) absent (Fig. R94:40); (1) present (Fig. R94:39).

126. (LR98:126, modified from R94:81) *Pubescence on lateral surface of metacoxa*: (0) restricted to two distinct lateral bands, no hairs in the middle; (1) less distinct and less regular bands, some hairs in the middle; (2) not arranged into bands, more evenly pubescent. Ordered.

127. (LR98:127) *Vertical strigae on lateral surface of metacoxa*: (0) absent; (1) present.

128. (R95:51, LR98:128) *Longitudinal carina on posterior surface of metatibia*: (0) absent; (1) present. *Ibalia* coded for actual state (1) rather than the likely ground-plan state of Ibaliidae.

129. (LR98:129, modified from R94:84) *Shape of 2r*: (0) simple or with a slight process or bend medially (Fig. R94:37); (1) with a prominent vein stump medially projecting anterolaterally (Fig. R94:38).

130. (LR98:130, modified from R94:85) *Length of 2r*: (0) long, ratio of length of 2r to length of R1 + Sc ≥ 0.73 (Fig. LR98:10b); (1) intermediate, ratio 0.47–0.65 (Fig. LR98:10a); (2) short, ratio ≤ 0.44 (Fig. R94:38). Ordered.

131. (LR98:131) *Direction of R1 laterad 2r*: (0) directed anteriorly, more or less perpendicular to anterior wing margin (Fig. LR98:10a); (1) directed more obliquely laterally (Fig. LR98:10b).

132. (LR98:132, ~RR00:14, ~R95:42, modified from R94:86) *Extent of R1*: (0) tubular along the entire anterior margin of marginal cell (Fig. R94:37); (1) tubular only along basal part of anterior margin of marginal cell; (2) ending at or close to anterior margin, not continuing laterally (Fig. R94:38); (3) ending distinctly before reaching anterior margin. Ordered. Coding complemented.

133. (R95:45, LR98:133, RR00:13) *Forewing areolet*: (0) absent (Figs R95:11a–b); (1) present (Figs R95:11c–d). Coding complemented. *Ibalia* coded for actual state (0) rather than the likely ground-plan state of the Ibaliidae.

134. (LR98:134) *Length of basalis*: (0) short, ratio of R1 + Sc to basalis >1.1(Fig. LR98:10a); (1) long, ratio < 1.1 (Fig. LR98:10b).

135. (R95:46, LR98:135) *Position of Rs + M, particularly the mesal end*: (0) situated closer to anterior margin of wing, its mesal end directed towards the middle or anterior half of the basalis (Figs R95:11a–c); (1) situated closer to the posterior margin of the wing, its mesal end directed towards the posterior end of basalis (Fig. R95:11d). Coding complemented.

136. (R95:44, LR98:136) *Bulla in R_1_ + Sc*: (0) absent (Figs R95:11a–b); (1) present (Figs R94:11c–d). Coding complemented.

137. (LR98:137) *Angle between R* + *Sc and R1* + *Sc*: (0) no change in direction, R + Sc smoothly continuing in R1 + Sc (Fig. LR98:10b); (1) distinct change in direction, R + Sc angled in relation to R1 + Sc (Fig. LR98:10a).

138. (LR98:138) *Shape of M at junction with 2r-m*: (0) distinctly angled; (1) straight (Fig. LR98:10a).

139. (LR98:139) *Hair-fringe along apical margin of wing*: (0) present; (1) very short or absent.

140. (R94:87, LR98:140, R99:5, RR00:18) *Size of 3tg of female*: (0) small, ratio of median length of 3tg to median length of 4tg < 1.0 (Fig. R94:41); (1) medium, ratio 1.5–1.8; (2) large, ratio > 2.0 (Fig. R94:42). Ordered. Coding complemented.

141. (R94:88, LR98:141) *Shape of anterior margin of 3tg of female*: (0) straight, not upcurved (Fig. R94:41); (1) upcurved (Fig. R94:42).

142. (LR98:142, modified from R95:57) *Shape of posterior margin of 3tg of female in lateral* *view*: (0) more or less vertical (Figs R94:41, R94:42); (1) distinctly oblique (Fig. LR98:11a). Coding complemented. Liopterids coded for actual state (1 for *Paramblynotus*, 0 for *Dallatorrella*) rather than the likely ground-plan state of family.

143. (R94:89, LR98:143, RR00:20) *Pubescence on 3tg of female*: (0) with a few scattered hairs or sparsely pubescent (Fig. R94:42); (1) with a dense hair patch anterolaterally (Fig. R94:41). Coding complemented.

144. (R94:90, LR98:144) *Relationship between 3tg and 4tg of female*: (0) 3tg and 4tg free sclerites (Fig. R94:42); (1) 3tg and 4 tg fused (Fig. R94:41).

 145. (R94:91, LR98:145) *Posterior margin of 4tg of female*: (0) straight or almost straight; (1) with a distinct, rounded incision medially.

 146. (R94:92, LR98:146) *Sculpture on 5tg of female*: (0) glabrous, not punctate; (1) closely and distinctly punctate.

147. (R94:93, LR98:147) *Anterior margin of 5tg of female*: (0) more or less deeply notched medially; (1) straight.

148. (R94:94, ~R95:54, LR98:148) *Ventral, marginal flange of petiole of female*: (0) absent (Fig. R94:44); (1) present, slightly projecting; (2) present, distinctly projecting (Fig. R94:43). Ordered. *Barbotinia* coding corrected from 2 to 0.

149. (R94:95, LR98:149) *Pubescence on ventral flange of petiole of female*: (0) flange nude, without hairs; (1) flange with a marginal fringe of hairs (Fig. R94:43).

150. (LR98:150) *Irregularly plicate to flabellate protuberance midventrally, close* *to posterior margin of petiole of female*: (0) absent; (1) present.

 151. (R94:96, LR98:151) *Sternal apodeme of 3st of female*: (0) distinct (Fig. R94:43); (1) indistinct or absent (Fig. R94:44).

 152. (R94:97, LR98:152) *Shape of apex of 7st of female*: (0) ventral spine not projecting, united almost to apex with lateral parts of 7st (Fig. R94:48); (1) ventral spine distinctly projecting, not united apically with lateral parts of 7st (Fig. R94:47).

153. (R94:98, LR98:153) *Size of ventral hairs on 7st of female*: (0) about the same length as hairs on 8tg (Fig. R94:48); (1) distinctly longer than hairs on 8tg (Fig. R94:47).

 154. (LR98:154) *Shape of ventral margin of metasoma of female in lateral view*: (0) oblique, more or less evenly rounded; (1) distinctly angled, anteriorly vertical, posteriorly horizontal (Fig. LR98:11a).

155. (R94:99, LR98:155) *Shape of dorsal margin of 9tg of female*: (0) straight, or very slightly curved dorsally for the attachment of the transverse intratergal muscle (Fig. R94:45); (1) distinctly curved dorsally to form a lobe for the attachment of the transverse intratergal muscle (Fig. R94:46).

 156. (R94:100, LR98:156) *Position of cercus of female*: (0) relatively close to apex of 9tg (Fig. R94:45); (1) well removed from apex of 9tg (Fig. R94:46).

157. (R94:101, LR98:157) *Insertion of cercus of female*: (0) cercus normally articulated to 9tg or more or less rigidly attached to 9tg (Fig. R94:46); (1) cercus inserted in a wide, slightly sclerotized membrane or plate in a hollow in 9tg (Fig. R94:45).

 158. (R94:103, LR98:158) *Shape of third valvula*: (0) distinctly broadened apically (Fig. R94:46); (1) not broadened apically (Fig. R94:45).

159. (LR98:159, modified from R94:102) *Length of terebra*: (0) long, articulation between second valvifer and second valvulae situated posterior to dorsalmost part of second valvifer (Fig. R94:46); (1) intermediate, position around dorsalmost part of second valvifer (Fig. R94:45); (2) short, position of articulation well anterior to dorsalmost part of second valvifer (Fig. LR98:11b). Ordered.

160. (R94:104, LR98:160) *Length of third valvula*: (0) short, projecting beyond apex of 9tg not more than one width of a third valvula (Fig. R94:46); (1) long, projecting beyond apex of 9tg more than one width of a third valvula (Fig. R94:45).

161. (R94:105, LR98:161) *Structure of 9tg of male*: (0) brown, at least partly sclerotized; (1) opaque, membranous except for anterior margin.

162. (R94:106, LR98:162) *Shape of apical part of aedeagus*: (0) only slightly expanded subapically (Fig. R94:49); (1) distinctly and abruptly expanded subapically (Fig. R94:50).

163. (R94:107, LR98:163) *Length of paramere*: (0) long, reaching beyond digitus (Fig. R94:49); (1) short, not reaching beyond digitus (Fig. R94:50).

164. (R94:108, LR98:164) *Shape of basidorsal margin of parameral plates*: (0) distinctly incised medially (Fig. R94:50); (1) not or only weakly incised medially (Fig. R94:49).

165. (= R99:1) *Antenna of female*: (0) connate; (1) moniliform.

166. (= R99:3) *Mesopleural furrow or carina*: (0) absent; (1) present.

167. (= R99:6) *Abdominal tergum 4 and 5 of female*: (0) free; (1) fused.

168. (~ R99:7) *Ovipositor articulation*: (0) absent; (1) present as a weak flexion point or as a distinct articulation. This coding lumps state 1 and 2 of R99:7.

169. (= R99:8) *Margin of ninth abdominal tergum of female*: (0) smooth, without any distinct processes; (1) with a distinct process just anterior to the flexion point. This feature is interpreted as independently derived from the similar but more posteriorly situated of some Cynipini (character LR98:155).

170. (= R99:10) *Apex of first valvula of ovipositor*: (0) narrow; (1) broad.

171. (= R99:apo1) *Shape of mesoscutellum*: (0) with more or less distinct dorsal, lateral and posterior surfaces, at least with some sculpture; (1) globose, smoothly rounded without distinct surfaces and without sculpture.

172. (= R99:apo2) *Scutellar plate*: (0) absent; (1) present.

173. (=R99:apo3) *Glandular release pit of mesoscutellum*: (0) absent; (1) present.

174. (= RR00:1) *Shape of head*: (0) roundish or squarish with large mouth and mandibles that only slightly overlap; (1) triangular with small mouth and broadly overlapping mandibles.

175. (= RR00:2) *Impression of lower face*: (0) absent; (1) present.

176. (= RR00:4) *Occiput*: (0) convexly rounded, without horizontal costulae or strigae; (1) flat or concave with horizontal costulae or strigae.

177. (~ RR00:6, part) *Lateral pronotal carina*: (0) usually short, with at least one distinct bend on each side where it changes slope; (1) long, continuous, and bent in one smooth bow from one side to the other. State 1 represents state 0 in RR00:6.

178. (~ RR00:6, part) *Ventral pronotal margin*: (0) not raised, distinctly separated from pronotal plate; (1) distinctly raised and fused with pronotal plate. State 0 represents states 1 and 2 in RR00 and state 1 represents state 3 and 4.

179. (~ RR00:9) *Median scutellar carina*: (0) absent; (1) present. State 2 in RR00:9 is autapomorphic for *Callaspidia* among the taxa studied here.

180. (= RR00:11) *Lateral propodeal carina*: (0) present and distinct; (1) absent or indistinct.

181. (~ RR00:12) *Longitudinal carina on posterior surface of metatibia*: (0) absent or weak; (1) strong. The double tibial carinae (state 2 in RE00:12) are autapomorphic for *Callaspidia* among the taxa studied here.

182. (= RR00:15) *Pubescence of forewing*: (0) covering most parts of wing; (1) largely absent.

183. (~ RR00:16) *Length of petiole*: (0) short, shorter than broad; (1) long, longer than broad. We lumped states 0 and 2 of RE00:16, since this interpretation is uncertain and needs to be confirmed by additional data from dissections.

184. (~ RR00:17) *Tergal flange of petiole*: (0) fused with the sternal part forming a continuous annulus; (1) distinct, separate from sternal flange (if the latter is present); (2) reduced or absent. States 2 and 3 of RR00:17 lumped since state 3 is autapomorphic for *Acanthaegilips* among the taxa examined here. Ordered 012.

185. (~ RR00:19) *Shape of third tergum*: (0) posterolateral margin rounded or straight; (1) posterolateral margin sinuous, tergum saddle-shaped. State 1 and 2 of RE00:10 were lumped since state 1 is autapomorphic for *Acanthaegilips* among the taxa examined here.

186. (~NL96:29) *Anterior lateral crest of metacoxa*: (0) absent; (1) present. Character states 1 and 2 lumped.

187. (=NL96:32) *Ratio of length of metatarsomere 1 to length of metatarsomeres 2-5 combined*: (0) < 2; (1) > 2.

188. (=NL96:33) *Direction of vein 2r of fore wing*: (0) 2r parallel to distal part of M; (1) 2r almost at a right angle to distal part of M.

189. (=NL96:37) *Shape of metasoma*: (0) moderately laterally compressed; (1) strongly laterally compressed, knifelike.

190. (=NL96:48) *Predominant sculpture on gena*: (0) irregular, punctate; (1) parallel.

191. (~NL96:50) *Sculpture on occiput*: (0) glabrous or occasionally horizontally strigate, not longitudinally costulate; (1) longitudinally costulate.

192. (=R95:apo, ~R95:41) *Shape of marginal cell*: (0) short, Rs/2r < 4.7; (1) long, ratio > 10.0.

193. (=R95:1) *Median frontal carina*: (0) absent; (1) present. (ci=0.60, ri=0.60).

194. (=R95:37) *Posterolateral propodeal process*: (0) present (Fig. 6); (1) absent. States reversed compared to Ronquist (1995).

195. (~R95:48) *Shape of metatrochanter*: (0) long, not bent laterad towards apex, anteriormost part of metafemur well removed from base of trochanter; (1) short, slightly to strongly bent laterad towards apex, anteriormost part of metafemur inserted close to base of trochanter.

196. (=R95:50) *Basal ring of metafemur*: (0) present; (1) absent.

197. (=R95:53) *Tubular process on metatarsomere 2*: (0) absent; (1) present.

198. (=R95:56) *Sculpture of tergal part of annulus*: (0) at least partly longitudinally sculptured; (1) smooth.

199. (=R95:58) *Size of abdominal tergum 7 of female*: (0) not enlarged; (1) conspicuously enlarged.

200. (R95:18) *Median dorsal length of pronotum behind anterior plate*: (0) short (Fig. 7b); (1) long (Fig. 7a). (ci=1.00, ri=1.00).

201. (R95:19) *Shape of pronotal crest*: (0) not incised medially (Fig. 8a); (1) with a small, but distinct, narrow incision medially (Fig. 8b). (ci=1.00, ri=1.00).

202. (R95:21) *Median length of dorsal pronotal area*: (0) short (not ill.); (1) long (Fig. 7b). (ci=1.00, ri=1.00).

203. (=R95:22) *Shape of lateral part of pronotum*: (0) sloping evenly downwards (Fig. 7b); (1) with distinct dorsal surface, laterad dorsal surface sloping abruptly downwards (Fig. 7a). (ci=1.00, ri=1.00).

204. (=R95:27) *Pair of posterior processes from dorsal surface of mesoscutellum*: (0) absent (Fig. 7b); (1) present (Fig. 7a). (ci=0.50, ri=0.50).

205. (=R95:31) *Mesofemoral groove*: (0) absent (Figs. 6, 9); (1) present (indicated in Fig. 5, but surface curvature difficult to see). (ci=1.00, ri=1.00).

206. (=R95:33) *Shape of mesosubpleuron*: (0) mesosubpleuron almost flat; (1) mesosubpleuron with distinct median, longitudinal impression. (ci=1.00, ri=1.00).

207. (=R95:34) *Shape of metapleural sulcus and metepisternum*: (0) metapleural sulcus abruptly bent at middle, metepisternum subrectangular (Fig. 5); (1) metapleural sulcus slightly and evenly curved, metepisternum subtriangular (Fig. 6). (ci=0.50, ri=0.67).

208. (=R95:35) *Position of metacoxal foramen*: (0) distance between anterior margin of metasubpleuron and foramen subequal to or longer than diameter of foramen (Fig. 5); (1) distance distinctly shorter than diameter of foramen (Fig. 6). (ci=1.00, ri=1.00).

209. (~R95:36) *Opening of propodeal spiracle*: (0) partly covered posteriorly by small, fingerlike process from outer margin of spiracular opening; (1) laterally and posteriorly covered by large, lidlike process (calyptra). State 0 removed (absent among the taxa studied here), state 1 recoded as state 0 and state 2 as state 1.

210. (R95:47) *Shape of basal part of mesofemur*: (0) subcylindrical; (1) distinctly expanded on the dorsal side.

211. (R95:49) *Shape of metafemur*: (0) long and slender, distinctly longer than metacoxa; (1) short and stout, about as long as metacoxa. (ci=1.00, ri=1.00).

212. (~R95:52) *Length of metatarsomere 1*: (0) shorter than or as long as metatarsomeres 2-5 combined (Fig. 14a); (1) longer than metatarsomeres 2-5 combined (Fig. 14b). (ci=0.50, ri=0.33).

213. (R95:64) *Shape of ventral end of lateral pronotal carina*: (0) curved posteriorly, not reaching ventral pronotal margin; (1) straight, vertical, reaching raised ventral pronotal margin. (ci=1.00, ri=1.00).

214. (R95:65) *Sculpture on lateral surface of pronotum*: (0) glabrous or punctate, sometimes partly costate-strigate; (1) at least partly foveate. (ci=0.50, ri=0.89).

215. (R95:71) *Laterodorsal process of mesoscutellum*: (0) absent; (1) indicated; (2) present, distinct. Ordered 012. (ci=0.50, ri=0.88).

216. (R95:76) *Mesopleural impression*: (0) absent; (1) present. (ci=0.33, ri=0.71).

217. (R95:77) *Lateroventral carina of mesopectus*: (0) completely absent (Fig. 9a; cf. also Figs. 5, 6); (1) at least partly present (Fig. 9b). (ci=1.00, ri=1.00).

218. (R95:78) *Shape of acetabulum and acetabular carina*: (0) acetabulum almost flat, acetabular carina v-shaped or u-shaped; (1) acetabulum divided into two furrows by strong median keel, acetabular carina ω-shaped. (ci=1.00, ri=1.00).

219. (R95:80) *Lateral depressions of dorsellum*: (0) absent or only indicated; (1) shallow but distinct; (2) deep. Ordered 012. (ci=0.50, ri=0.80).

220. (R95:81) *Position of upper part of metapleural sulcus*: (0) meeting anterior metapectal margin at about mid-height of margin; (1) meeting anterior metapectal margin far above mid-height of margin. (ci=1.00, ri=1.00).

221. (R95:84) *Intermetacoxal processes*: (0) absent; (1) present. (ci=1.00, ri=1.00).

222. (R95:86) *Insertion of metasoma on propodeum*: (0) low, postsubpleuron short; (1) high, postsubpleuron long. (ci=0.50, ri=0.88).

223. (R95:92) *Length of metatibia*: (0) longer than metafemur; (1) shorter than metafemur. (ci=0.50, ri=0.89).

224. (R95:93) *Metatibial lobe*: (0) absent; (1) present. (ci=1.00, ri=1.00).

225. (R95:100) *Abdominal sterna 4-6 (females)*: (0) at least partly exposed; (1) entirely covered by abdominal sternum 3. (ci=0.50, ri=0.86).

226. (~R95:110) *Shape of eudorsal margin of abdominal tergum 8 in lateral view (females)*: (0) vertical to declivous; (1) reclivous.

227. *Sculpture on gena*: (0) smooth or irregular, without vertical carinae; (1) composed of 5-9 vertical carinae .

228. *Rows of transverse , sharp costulae on ventral side of profemur*: (0) absent (1) present

*Biological characters*

229. *Geographic distribution*: (0) Palearctic; (1) Nearctic; (2) Neotropic; (3) Australian; (4) South African. Unordered.

230. *Feeding niche of larva*: (0) insect parasitoid; (1) inquiline; (2) gall inducer. Unordered.

231. (R99:12) *Microhabitat of larva*: (0) wood; (1) plant gall; (2) aphid community; (3) decomposing organic matter (fungi, dung, carrion, rotting fruit, wilting plants) or in living plants but not in galls or wood. Unordered.

232. (R99:11) *Host insect order*: (0) Hymenoptera; (1) Coleoptera; (2) Neuroptera; (3) Diptera; (4) Lepidoptera. Unordered.

233. (LR98:166) *Host plant family*: (0) Fagaceae or Nothofagaceae; (1) Rosaceae; (2) Fabaceae; (3) Anacardiaceae; (4) Sapindaceae; (5) Asteraceae; (6) Lamiaceae; (7) Papaveraceae; (8) Valerianaceae; (9) Salicaceae. This character was coded also for the parasitoids of gall insects in the Thrasorinae and Parnipinae (Figitidae). Other insect parasitoids in the Cynipoidea do not appear to be restricted to attacking hosts living on a single host-plant family. Nothofagaceae and Fagaceae were treated as a single state because of the close relationship between these two families. Unordered.

234. *Host plant growth form*: (0) woody; (1) herbaceous. This character was coded also for the parasitoids that attack hosts inside plants.

235. *Gall structure*: (0) cryptic, causing no external deformation of the plant and minimal change to the tissue outside the inner gall; (1) distinct swelling, outer gall prominent but of same structure as adjacent parts of the plant; (2) complex, outer gall prominent and radically different from adjacent parts of the plant. Unordered.

236. *Gall position*: (0) reproductive organs, such as fruit, seed or inflorescence; (1) undifferentiated bud; (2) leaf; (3) stem, twig, or runner; (4) root. Unordered.

237. *Gall chambers*: (0) single, galls isolated from each other (this includes some herb stem galls where the chambers are not clustered adjacent to each other); (1) many, individual galls merge into a large common gall.

238. *Gall attachment*: (0) integral or occasionally semi-detachable (can be broken off plant without causing much damage); (1) detachable, normally falls from plant when mature.

239. (LR98:165) *Reproduction*: (0) normal sexual reproduction, no alternating generations; (1) alternating sexual and agamic generations. Some of the Cynipini species studied here are only known from one generation but it is presumed that they have alternating generations. In all cases, other members of the same genus are known to have alternating generations.

*Remarks on omitted characters*

RR00:3. Same as LR98:8, coding complemented.

RR00:5. Same as LR98:48, coding complemented.

RR00:7. Same as LR98:75.

RR00:8. Uninformative in the context of the present analysis.

RR00:13. Same as LR98:133, coding complemented.

RR00:18. Same as LR98:140.

RR00:20. Same as LR98:143, coding complemented.

RR00:21. Uninformative in the context of the present analysis.

RR00:22. Equivalent to biological character 4 in this study.

R99:2. Same as LR98:75, coding complemented.

R99:4. Uninformative in the context of the present analysis. Note that characters 4 and 5 appear in the wrong order in Table 3 of R99.

R99:5. Same as LR98:140, coding complemented.

R99:9. Omitted; data missing for the majority of taxa in the present analysis.

R99:11. Equivalent to biological character 4 in this study.

R99:12. Equivalent to biological character 3 in this study.

NL96:4. Same as LR98:48; coding complemented.

NL96:34. Same as LR98:133, coding complemented.

R95:10. Same as LR98:48, coding complemented.

R95:23. Same as LR98:60, coding complemented.

R95:32. Same as LR98:89, coding complemented.

R95:42. Same as LR98:132, coding complemented.

R95:44. Same as LR98:136, coding complemented.

R95:45. Same as LR98:133, coding complemented.

R95:46. Same as LR98:135, coding complemented.

R95:55. Same as RR00:17, coding complemented.

R95:57. Same as LR98:142, coding complemented.
