# Supplementary figures and images for: Phylogeny, Evolution and Classification of Gall Wasps: The Plot Thickens
Source: PLoS One. 2015 May 20;10(5):e0123301. doi: 10.1371/journal.pone.0123301 (PMC4439057; doi:10.1371/journal.pone.0123301)

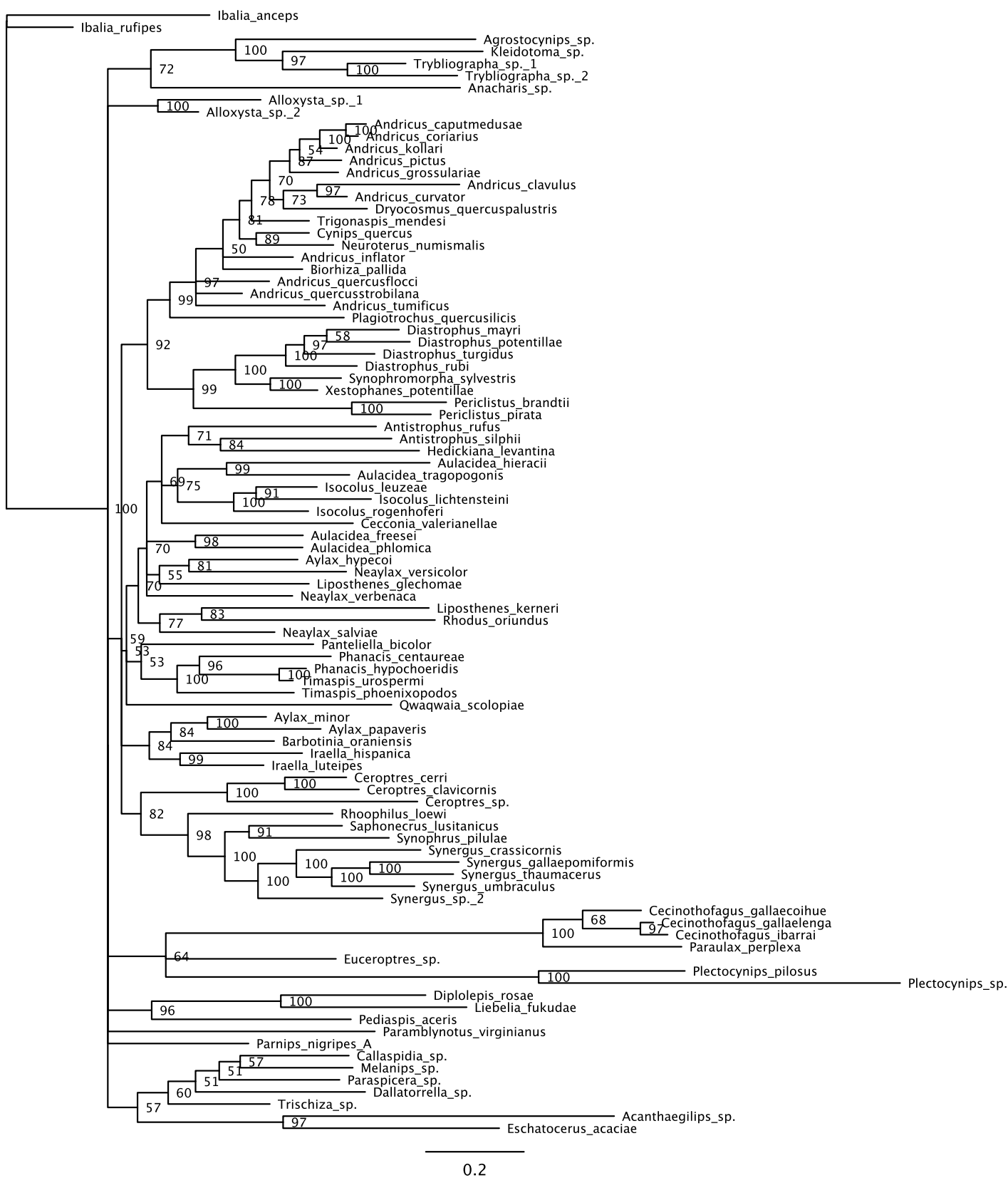

S1 Figure. Gene tree for COI with posterior clade probabilities (%) on branches.

Supplement: S1 Fig — Results from Bayesian MCMC analysis, with posterior clade probabilities. (PDF) [file pone.0123301.s006.pdf]

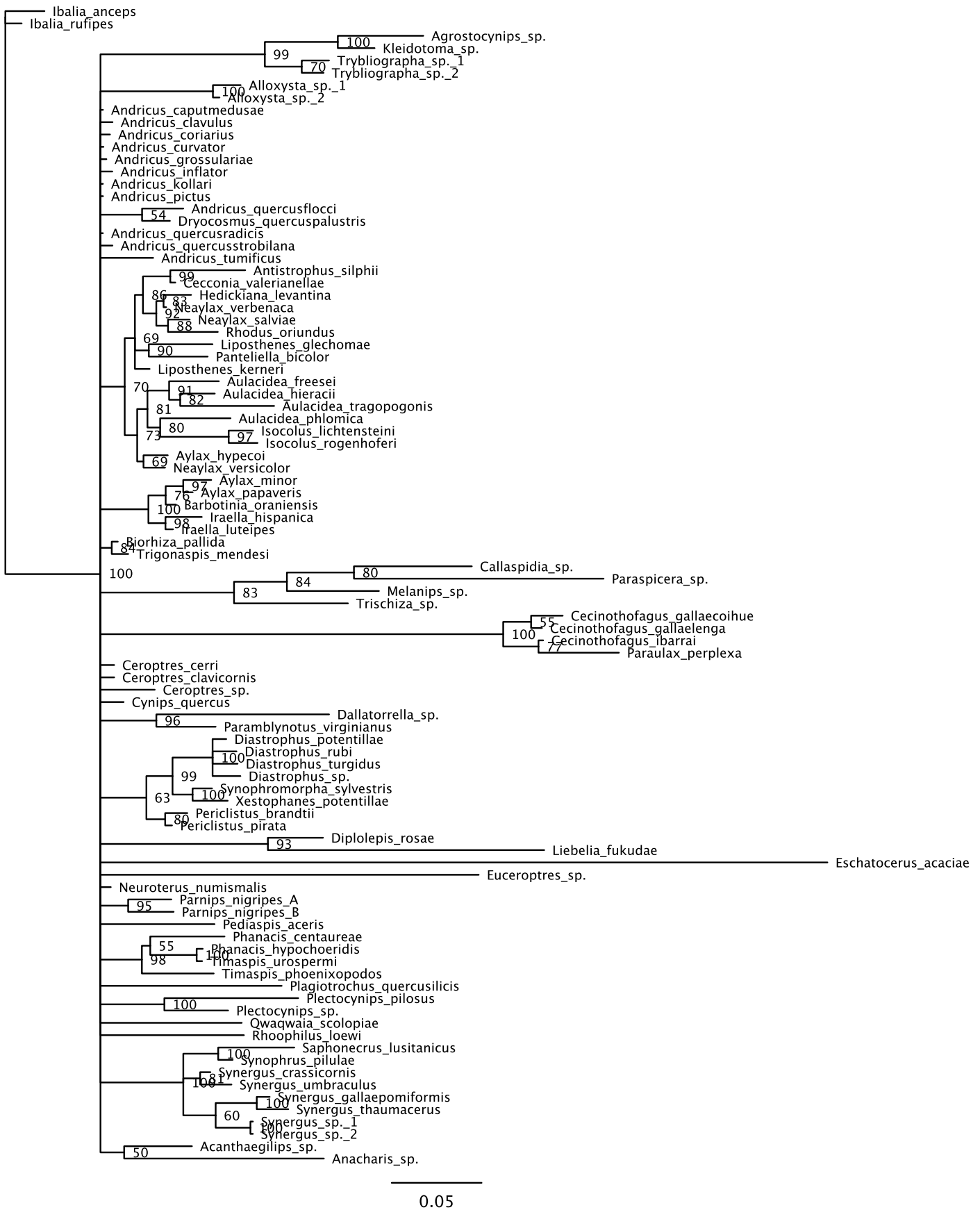

S2 Figure. Gene tree for 28S with posterior clade probabilities (%) on branches.

Supplement: S2 Fig — Results from Bayesian MCMC analysis, with posterior clade probabilities. (PDF) [file pone.0123301.s007.pdf]

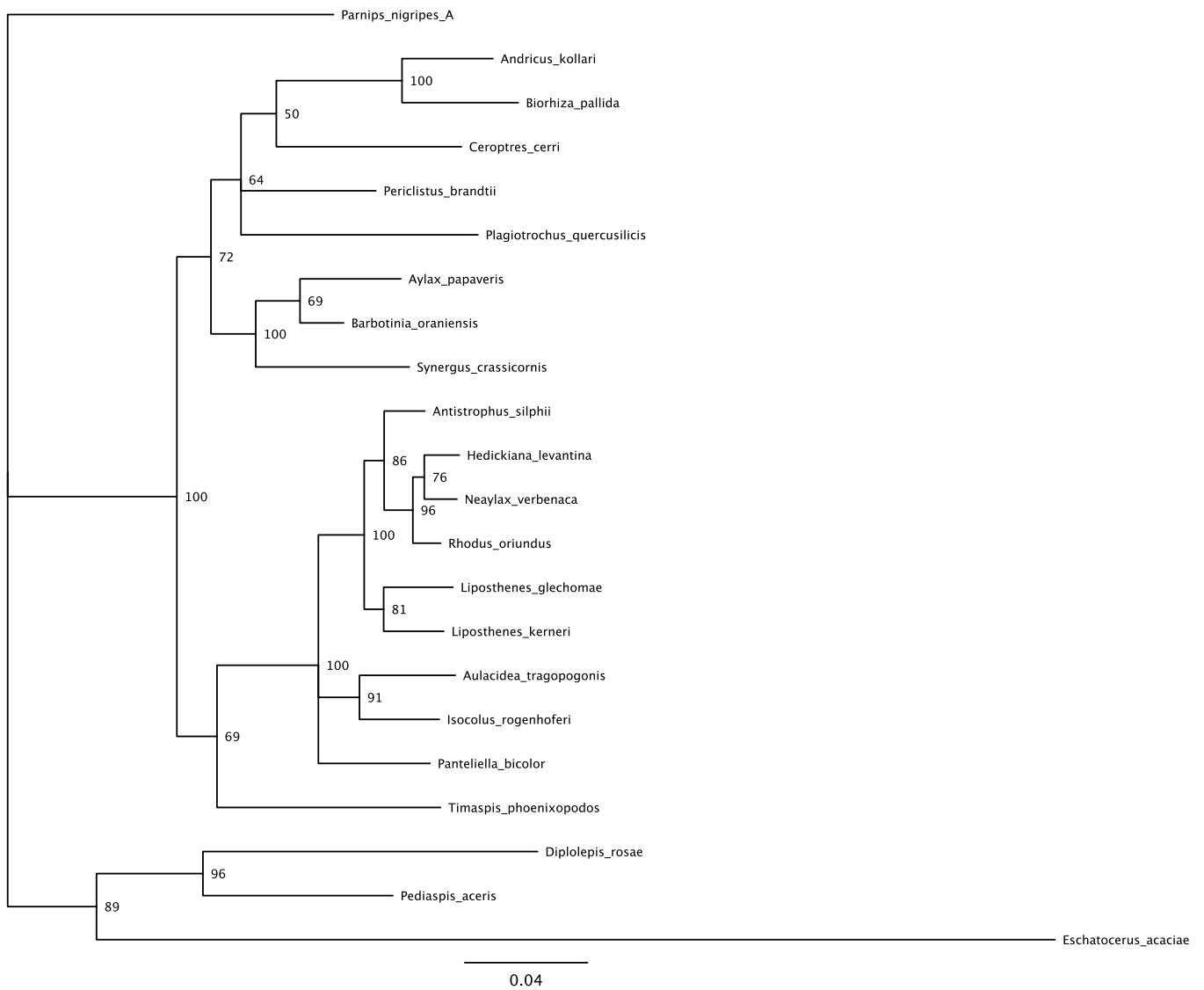

S3 Figure. Gene tree for LWRh with posterior clade probabilities (%) on branches.

Supplement: S3 Fig — Results from Bayesian MCMC analysis, with posterior clade probabilities. (PDF) [file pone.0123301.s008.pdf]

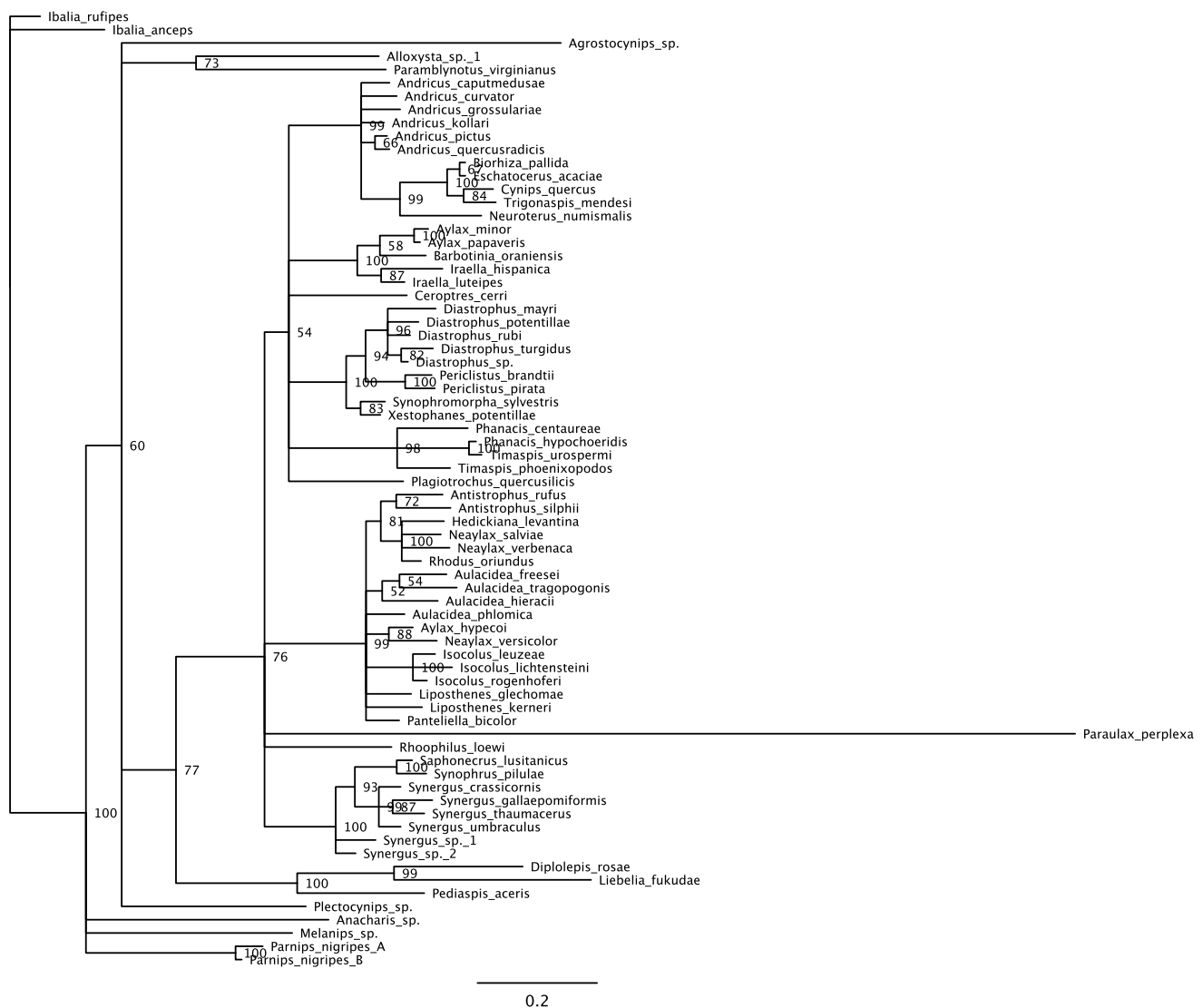

S4 Figure. Gene tree for EF1aF1 with posterior clade probabilities (%) on branches.

Supplement: S4 Fig — Results from Bayesian MCMC analysis, with posterior clade probabilities. (PDF) [file pone.0123301.s009.pdf]

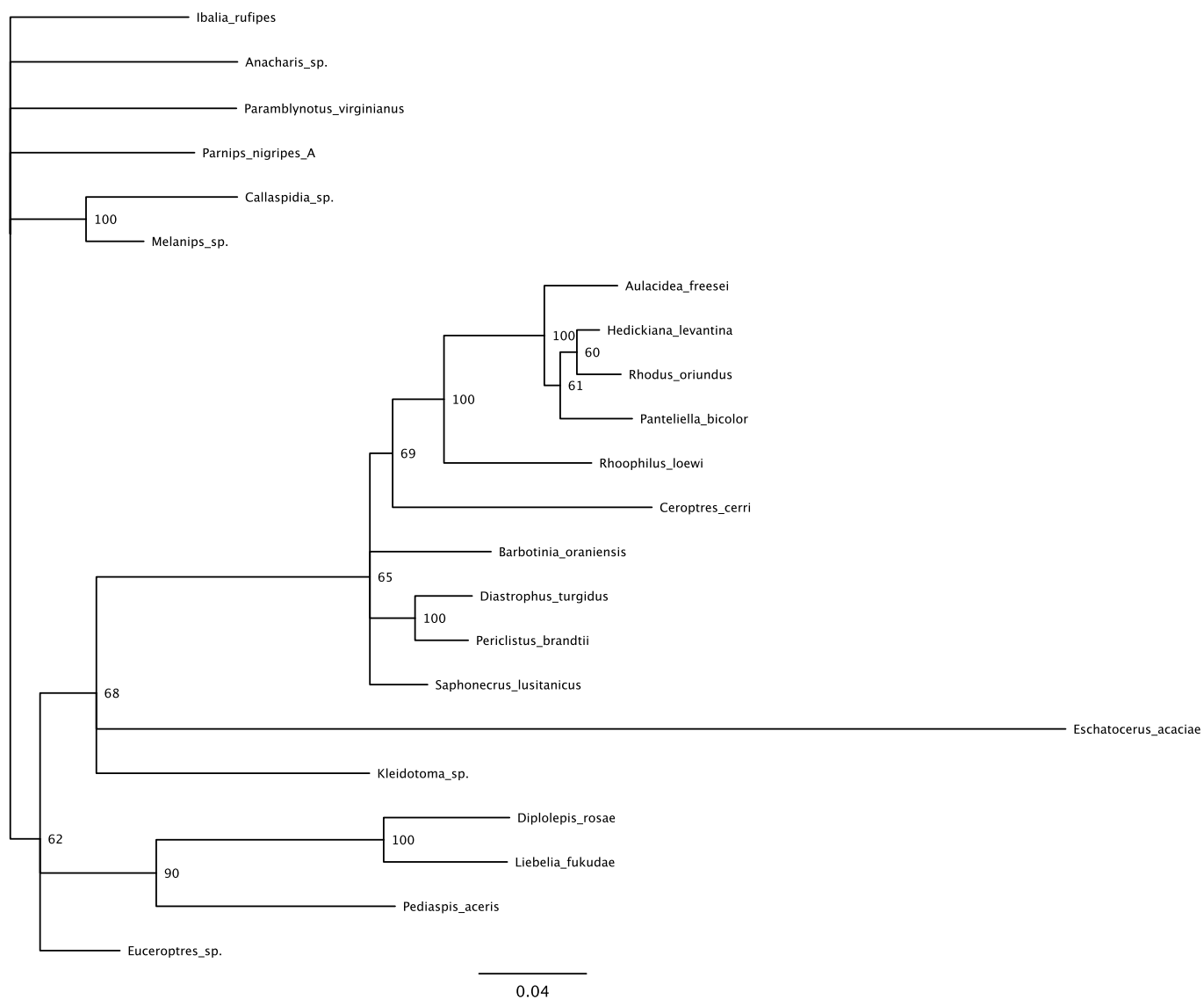

S5 Figure. Gene tree for EF1aF2 with posterior clade probabilities marked on branches.

Supplement: S5 Fig — Results from Bayesian MCMC analysis, with posterior clade probabilities. (PDF) [file pone.0123301.s010.pdf]
