# Supplementary material for: Phylogeny, Evolution and Classification of Gall Wasps: The Plot Thickens
Source: PLoS One. 2015 May 20;10(5):e0123301. doi: 10.1371/journal.pone.0123301 (PMC4439057; doi:10.1371/journal.pone.0123301)
Supplement: S7 Fig — Results from Bayesian MCMC analysis, with posterior clade probabilities. (PDF) [file pone.0123301.s012.pdf]

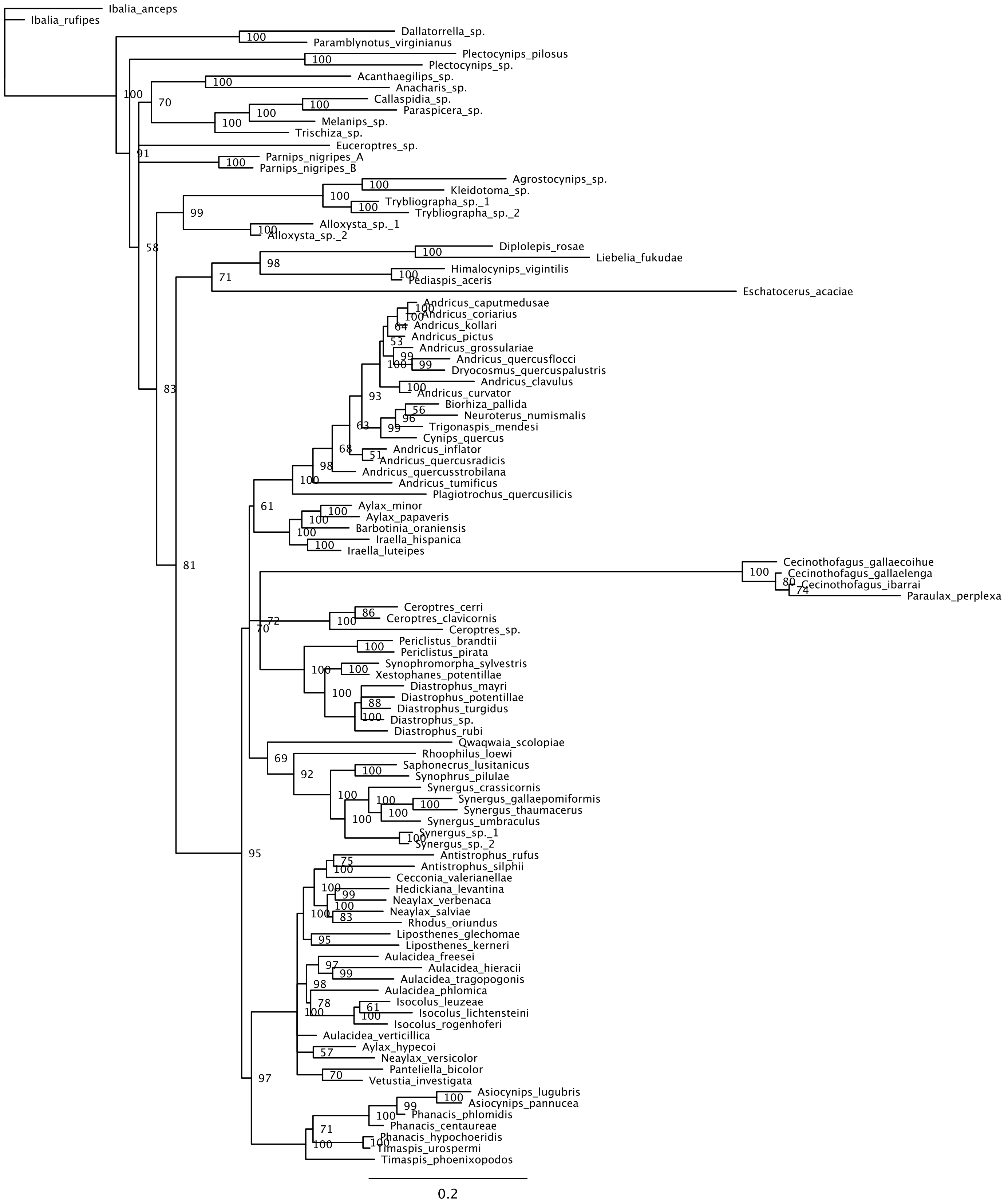

S7 Figure. Tree based on morphology and molecules, without biological data, with posterior clade probabilities (%) on branches.
