# Supplementary material for: Phylogeny, Evolution and Classification of Gall Wasps: The Plot Thickens
Source: PLoS One. 2015 May 20;10(5):e0123301. doi: 10.1371/journal.pone.0123301 (PMC4439057; doi:10.1371/journal.pone.0123301)
Supplement: S10 Fig — Results from Bayesian MCMC analysis, with posterior clade probabilities. (PDF) [file pone.0123301.s015.pdf]

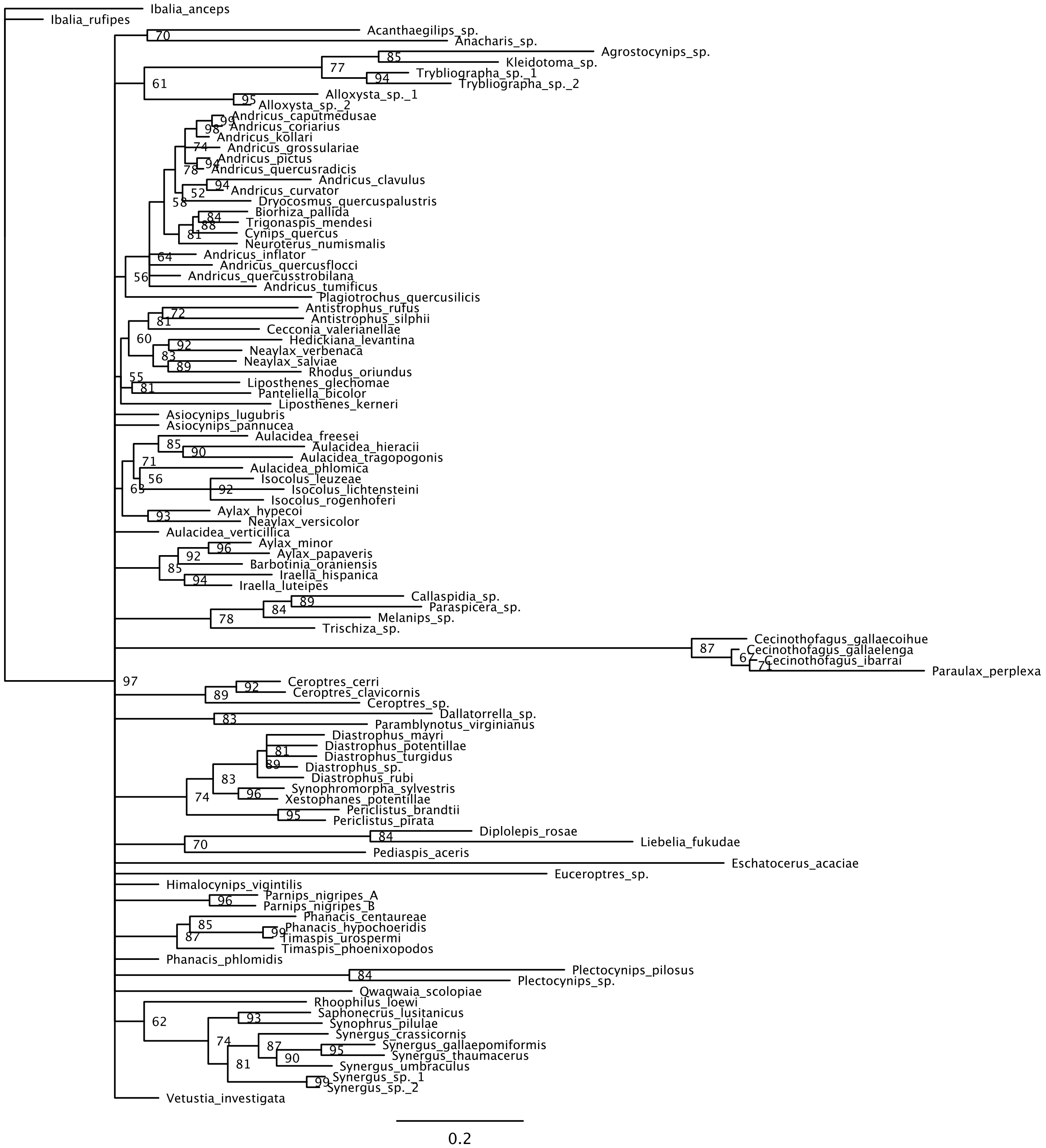

S10 Figure. Analysis of molecular data with incompletely coded gene regions removed. Posterior clade probabilities (%) on branches.
