# Supplementary material for: Phylogeny, Evolution and Classification of Gall Wasps: The Plot Thickens
Source: PLoS One. 2015 May 20;10(5):e0123301. doi: 10.1371/journal.pone.0123301 (PMC4439057; doi:10.1371/journal.pone.0123301)
Supplement: S1 Table — Detailed information on the taxa and sequences used in the analyses, including sequence accession numbers. (DOC) [file pone.0123301.s016.doc]

**S1 Table**. **Studied taxa**. Gall wasp taxa (Cynipoidea) included in the analysis, together with brief information on their life history. For the Cynipidae, we give both the current classification, followed by the one proposed here in square brackets, if different. Genbank accession numbers are given for all DNA sequences, and a TreeBase reference (this study) for the morphological data. Missing data are indicated by "–".

| **Taxon** | **Voucher^a^** | **Collecting site** | **Leg^b^** | **Life history^c^** | **Host^d^** | **Morphol.^e^** | **COI** | **28S** | **EF1** | **EF12** | **LWRh** |
| --- | --- | --- | --- | --- | --- | --- | --- | --- | --- | --- | --- |
| CYNIPIDAE |  |  |  |  |  |  |  |  |  |  |  |
| Synergini s. lat. [Ceroptresini] | | | | | | | | | |  |  |
| *Ceroptres cerri* | FR491 FR498 | Madrid, Spain Hungary | JLNA GS | Inquiline | *Plagiotrochus* spp. (Cy) | – | AY368910 – | AY368935 – | DQ012555 LN811093 | – LN811111 | AY371052 – |
| *Ceroptres clavicornis* | ZL011 | Spain | JPV | Inquiline | Cynipini | 15832 | DQ012635 | DQ012593 | – | – | – |
| *Ceroptres* sp. | FR399 | Kentucky, USA | EE | (Inquiline) | *Andricus quercuscornigera* | – | DQ012636 | DQ012594 | – | – | – |
|  |  |  |  |  |  |  |  |  |  |  |  |
| Synergini s. lat. [Synergini s. str.] | | | | | | | | | | | |
| *Rhoophilus loewi* | FRj021 | Cape Province, South Africa | SvN | Inquiline | *Scyrotis* (Ce) (Anacardiaceae) | 15832 | DQ012650 | DQ012607 | DQ012569 | LN811112 | – |
| *Saphonecrus lusitanicus* | FRj013 | Salamanca, Spain | JLNA | Inquiline | *Plagiotrochus* (Cy) | 15832 | DQ012651 | DQ012608 | LN811094 | LN811113 | – |
| *Synergus crassicornis* | FRj011 | Arganda, Spain | JLNA | Inquiline | *Plagiotrochus* (Cy) | 15832 | AY368909 | AY368936 | AY368962 | – | AY371051 |
| *Synergus gallaepomiformis* | FRj009 | Tiszafured, Hungary | GS | Inquiline | Cynipini (Cy) | – | DQ012652 | DQ012610 | AF395167 | – | – |
| *Synergus thaumacerus* | FR308 | Madrid, Spain | JLNA | Inquiline | *Trigonaspis* (Cy) | – | DQ012654 | DQ012612 | DQ012573 | – | – |
| *Synergus umbraculus* | FRj014 | Madrid, Spain | JLNA | Inquiline | Cynipini | – | DQ012655 | DQ012613 | – | – | – |
| *Synergus* sp. 1 | FRf004 | Texas, USA | FR | (Inquiline) | Unknown | – | – | DQ012609 | DQ012571 | – | – |
| *Synergus* sp. 2 | FR400 | Kentucky, USA | EE | Inquiline | *Andricus quercuscornigera* (Cy) | – | DQ012653 | DQ012611 | DQ012572 | – | – |
| *Synophrus pilulae* | FRf248 | Senckut, Hungary | FR | Inquiline | *Andricus burgundus* (Cy) | 15832 | DQ012656 | LN811092 | LN811095 | – | – |
|  |  |  |  |  |  |  |  |  |  |  |  |
| Synergini s. lat. [Diastrophini part.] | | | | | | | | | | | |
| *Periclistus brandtii* | FRj002 FR615 | Knivsta, Sweden Sweden | FR FR | Inquiline | *Diplolepis* (Di) | 15832 | AF395181 – | AF395152 – | AF395173 LN811096 | –  LN811114 | AF395189 – |
| *Periclistus piratus* | FR413 | Bouchette, Canada | FR | Inquiline | *Diplolepis* (Di) | – | DQ012649 | DQ012606 | DQ012567 | – | – |
| *Synophromorpha sylvestris* | FR401 | Virginia, USA | DS | Inquiline | *Diastrophus nebulosus* (Ay) | 15832 (*S. rubi*) | AY368911 | AY368937 | AY368961 | – | – |
|  |  |  |  |  |  |  |  |  |  |  |  |
| Aylacini s. lat. [Aylacini s. str.] | | | | | | | | | | | |
| *Aylax hypecoi* | FR505 | Plovdiv, Bulgaria | AS | Fruit galler (H) | *Hypecoum* (Pa) | – | DQ012630 | DQ012588 | DQ012552 | – | – |
| *Aylax minor* | FRf287 | Madrid, Spain | JLNA | Fruit galler (H) | *Papaver* (Pa) | – | DQ012631 | DQ012589 | DQ012553 | – | – |
| *Aylax papaveris* | FR339 | Vombsjön, Sweden | FR | Fruit galler (H) | *Papaver* (Pa) | 15832 | AY368923 | AY368949 | AY368974 | – | AY371061 |
| *Barbotinia oraniensis* | FRj003 | Madrid, Spain | FR | Fruit galler (H) | *Papaver* (Pa) | 15832 | AF395179 | AF395150 | AF395171 | LN811115 | AF395187 |
| *Iraella* *hispanica* | FR580 | Tarragona, Spain | JLNA | Flower galler (H) | *Papaver* (Pa) | – | DQ012632 | DQ012590 | DQ012554 | – | – |
| *Iraella luteipes* | FR333 | Madrid, Spain | JLNA | Stem galler (H) | *Papaver* (Pa) | 15832 | AY368924 | AY368950 | AY368975 | – | – |
|  |  |  |  |  |  |  |  |  |  |  |  |
| Aylacini s. lat. [Aulacideini] | | | | | | | | | | | |
| *Antistrophus rufus* | FR493 | Illinois, USA | JT | Stem galler (H) | *Silphium* (As) | – | DQ012626 | – | DQ012548 | – | – |
| *Antistrophus silphii* | FRf239 | Illinois, USA | JT | Stem galler(H) | *Silphium* (As) | 15832 (*A. pisum*) | AY368917 | AY368943 | AY368968 | – | AY371055 |
| *Aulacidea freesei* | FR577 | Madrid, Spain | JLNA | Stem galler (H) | *Silybum* (As) | – | DQ012627 | DQ012585 | DQ012549 LN811097 | LN811116 | – |
| *Aulacidea hieracii* | FR410 | Äspet, Sweden | FR | Stem galler (H) | *Hieracium* (As) | – | DQ012628 | DQ012586 | DQ012550 | – | – |
| *Aulacidea phlomica* | FRf242ph | Szalas, Hungary | FR | Stem galler (H) | *Phlomis* (La) | 15832 | DQ012629 | DQ012587 | DQ012551 | – | – |
| *Aulacidea tragopogonis* | FRf265 | Madrid, Spain | FR | Stem galler (H) | *Tragopogon* (As) | 15832 | AY368922 | AY368948 | AY368973 | – | AY371060 |
| *Aulacidea verticillica* |  |  |  | Flower galler (H) | *Salvia* (La) | 15832 | – | – | – | – | – |
| *Cecconia valerianellae* | FR674 | Ebeltoft, Denmark | FR | Fruit galler (H) | *Valerianella* (Va) | 15832 | LN811086 | LN811089 | – | – | – |
| *Hedickiana levantina* | FRj017 | Judayta, Jordan | BM | Stem galler (H) | *Salvia* (La) | 15832 | AY368919 | AY368945 | AY368970 LN811098 | LN811117 | AY371057 |
| *Isocolus leuzeae* | FR500 | Guadalajara, Spain | JLNA | Fruit galler (H) | *Leuzea* (As) | – | DQ012643 | – | DQ012561 | – | – |
| *Isocolus lichtensteini* | FR329 | Madrid, Spain | JLNA | Stem galler (H) | *Centaurea* (As) | – | DQ012644 | DQ012600 | DQ012562 | – | – |
| *Isocolus scabiosae* | FRf243 | Uppsala, Sweden | FR | Fruit & stem galler (H) | *Centaurea* (As) | 15832 | AY368921 | AY368947 | AY368972 | – | AY371059 |
| *Liposthenes glechomae* | FRf003 | Skogsby, Sweden | FR | Stem galler (H) | *Glechoma* (La) | 15832 | AY368915 | AY368941 | AY368966 | – | AY371053 |
| *Liposthenes kerneri* | FR323 | Madrid, Spain | JLNA | Fruit galler (H) | *Nepeta* (La) | 15832 | AY368916 | AY368942 | AY368967 | – | AY371054 |
| *Neaylax salviae* | FR328 | Madrid, Spain | JLNA | Fruit galler (H) | *Salvia* (La) | 15832 | DQ012646 | DQ012602 | DQ012564 | – | – |
| *Neaylax verbenaca* | FR326 | Madrid, Spain | JLNA | Flower galler (H) | *Salvia* (La) | – | AY368920 | AY368946 | AY368971 | – | AY371058 |
| *Neaylax versicolor* | FRj019 | Salamanca, Spain | JLNA | Fruit galler (H) | *Fumaria* (Pa) | – | DQ012647 | DQ012603 | DQ012565 | – | – |
| *Panteliella bicolor* | FRf242 | Szalas, Hungary | FR | Leaf & stem galler (H) | *Phlomis*(La) | 15832 (as *P. fedtschenkoi*) | AF395180 | AF395153 | AF395172 LN811099 | LN811118 | AF395188 |
| *Rhodus oriundus* | FRj010 | Creete, Greece | FR | Bud galler (H) | *Salvia* (La) | 15832 | AY368918 | AY368944 | AY368969 LN811100 | LN811119 | AY371056 |
| *Vetustia investigata* |  |  |  | Flower galler (H) | *Phlomis* (La) | 15832 | – | – | – | – | – |
|  |  |  |  |  |  |  |  |  |  |  |  |
| Aylacini s. lat. [Phanacidini] | | | | | | | | | | | |
| *Asiocynips lugubris* |  |  |  | – | – | 15832 | – | – | – | – | – |
| *Asiocynips pannucea* |  |  |  | – | – | 15832 | – | – | – | – | – |
| *Phanacis hypochoeridis* | FR321 | Madrid, Spain | FR | Stem galler (H) | *Hypochoeris* (As) | 15832 | AY368926 | AY368952 | AY368977 | – | – |
| *Phanacis centaureae* | FR344 | Madrid, Spain | FR | Stem galler (H) | *Centaurea* (As) | 15832 | AY368927 | AY368953 | AY368978 | – | – |
| *Phanacis phlomidis* |  |  |  | Stem galler (H) | *Phlomis* (La) | 15832 | – | – | – | – | – |
| *Timaspis phoenixopodos* | FRf263 FR330 | Madrid, Spain Madrid, Spain | FR JLNA | Stem galler (H) | *Lactuca* (As) | 15832 | AY368925 – | AY368951 – | AY368976 LN811101 | – – | AY371062 |
| *Timaspis urospermi* | FR504 | Malaga, Spain | JLNA | Stem galler (H) | *Urospermum* (As) | – | DQ012657 | DQ012614 | DQ012574 | – | – |
|  |  |  |  |  |  |  |  |  |  |  |  |
| Aylacini s. lat. [Diastrophini part.] | | | | | | | | | | | |
| *Diastrophus mayri* | FRj020 | Härnösand, Sweden | OP | Stem galler (H) | *Potentilla* (Ro) | – | DQ012639 | – | DQ012558 | – | – |
| *Diastrophus* sp. | FR398 | Kyoto, Japan | FR | Galler (H) | *Rubus* (Ro) | – | – | DQ012597 | DQ012557 | – | – |
| *Diastrophus turgidus* | FRf289 | Quebec, Canada | FR | Stem galler (W) | *Rubus* (Ro) | 15832 | AY368913 | AY368939 | AY368964 | LN811120 | – |
| *Diastrophus potentillae* | FR403 | New York, USA | EQ | Stem galler (H) | *Potentilla* (Ro) | 15832 | AY368914 | AY368940 | AY368965 | – | – |
| *Diastrophus rubi* | FR406 | Sweden | OP | Stem galler (W) | *Rubus* (Ro) | – | DQ012640 | DQ012598 | DQ012559 | – | – |
| *Xestophanes potentillae* | FRf238 | Knivsta, Sweden | FR | Stem galler (H) | *Potentilla* (Ro) | 15832 | AY368912 | AY368938 | AY368963 | – | – |
|  |  |  |  |  |  |  |  |  |  |  |  |
| Paraulacini | | | | | | | | | | | |
| *Cecinothofagus gallaecoihue* | JLNA | Puerto Varas, Chile | JLNA | Inquiline? | *Aditrochus* (Nothofagaceae) | – | FJ998296 | FJ998292 | – | – | – |
| *Cecinothofagus gallaelenga* | JLNA | Punta Arenas, Chile | JLNA | Inquiline? | *Aditrochus* (Nothofagaceae) | – | FJ998297 | FJ998293 | – | – | – |
| *Cecinothofagus ibarrai* | JLNA | Ensenada, Chile | JLNA | Inquiline? | *Aditrochus* (Nothofagaceae) | 15832 | FJ998298 | FJ998294 | – | – | – |
| *Paraulax perplexa Paraulax* sp. | JLNA FR492 | Los Queules, Chile Conguillio, Chile | JLNA FR |  | Inquiline? | 15832 | FJ998299 – | FJ998295 – | – LN811102 | – – | – – |
|  |  |  |  |  |  |  |  |  |  |  |  |
| Qwaqwaiini | | | | | | | | | | | |
| *Qwaqwaia scolopiae* | FR675 | Wakerstroom, South Africa | SN | Galler (W) | *Scolopia* (Sa) | 15832 | LN811088 | LN811091 | – | – | – |
|  |  |  |  |  |  |  |  |  |  |  |  |
| Eschatocerini | | | | | | | | | | | |
| *Eschatocerus acaciae* | FRf281 | Tucumán, Argentina | SO | Galler (W) | *Acacia*, *Prosopis* (Fa) | 15832 | AY368928 | AY368954 | AY368979 | LN811121 | AY371063 |
|  |  |  |  |  |  |  |  |  |  |  |  |
| Diplolepidini | | | | | | | | | | | |
| *Diplolepis rosae* | FRj001 DM201 | Knivsta, Sweden Sweden | FR FR | Fruit, bud, leaf galler (W) | *Rosa* (Ro) | 15832 | AF395174 – | AF395157 – | AF395166 – | – LN811122 | AF395182 |
| *Liebelia fukudae* | FRf254 | Tanesashi, Japan | YA | Bud galler (W) | *Rosa* (Ro) | 15832 | DQ012645 | DQ012601 | DQ012563 LN811103 | LN811123 | – |
|  |  |  |  |  |  |  |  |  |  |  |  |
| Pediaspidini | | | | | | | | | | | |
| *Pediaspis aceris* | FRf240 | Sopron, Hungary | FR | Part: Stem galler (W)  Sex: Leaf galler (W) | *Acer* (Sa) | 15832 | AY368929 | AY368955 | LN811104 | LN811124 | AY371064 |
| *Himalocynips vigintilis* |  | Nepal |  |  |  | 15832 | – | – | – | – | – |
|  |  |  |  |  |  |  |  |  |  |  |  |
| Cynipini | | | | | | | | | | | |
| *Andricus caputmedusae* | FRf251 | Demjén, Hungary | FR | Part: Fruit galler (W) | *Quercus* (Fg) | 15832 | DQ012619 | DQ012578 | DQ012544 | – | – |
| *Andricus clavulus* | ZL015 | Connecticut, USA | ZL | Stem galler (W) | *Quercus* (Fg) | – | DQ012633 | DQ012591 | – | – | – |
| *Andricus coriarius* | FRf264 | Madrid, Spain | JLNA | Part: Bud galler (W) | *Quercus* (Fg) | – | DQ012620 | DQ012579 | – | – | – |
| *Andricus curvator* | FRj007 | Jabyzanlsza, Hungary | GS | Part: Bud galler (W)  Sex: Leaf galler (W) | *Quercus* (Fg) | 15832 | DQ012621 | AF395155 | AF395169 | – | – |
| *Andricus grossulariae*^f^ | FRf257 | Madrid, Spain | JLNA | Part: Flower galler (W)  Sex: ? | *Quercus* (Fg) | 15832 | DQ012624 | DQ012582 | DQ012545 | – | – |
| *Andricus inflator* | ZL013 | Uppsala, Sweden | ZL | Part: Bud galler (W)  Sex: Stem galler (W) | *Quercus* (Fg) | – | DQ012623 | DQ012581 | – | – | – |
| *Andricus kollari* | FRj006 | Jabyzanlsza, Hungary | GS | Part: Stem galler (W)  Sex: Bud galler (W) | *Quercus* (Fg) | – | AF395176 | AF395156 | AF395168 | – | AF395184 |
| *Andricus pictus* | FRf256 | Madrid, Spain | JLNA | Part : Bud galler (W)  Sex: ? | *Quercus* (Fg) | – | DQ012625 | DQ012583 | DQ012546 | – | – |
| *Andricus quercusflocci* | ZL012 | New York, USA | ZL | Part: Leaf galler (W)  Sex: ? | *Quercus* (Fg) | 15832 | DQ012622 | DQ012580 | – | – | – |
| *Andricus quercusradicis* | FRf260 | Madrid, Spain | FR | Part: Root galler (W)  Sex: Stem galler (W) | *Quercus* (Fg) | 15832 | – | DQ012584 | DQ012547 | – | – |
| *Andricus quercusstrobilana* | ZL014 | New York, USA | ZL | Stem galler (W) | *Quercus* (Fg) | – | DQ012617 | DQ012576 | – | – | – |
| *Andricus tumificus* | ZL016 | Pennsylvania, USA | EQ | Leaf galler (W) | *Quercus* (Fg) | – | DQ012634 | DQ012592 | – | – | – |
| *Biorhiza pallida* | FRf286 | Stavsudda, Sweden | FR | Part: Root galler (W)  Sex: Bud galler (W) | *Quercus* (Fg) | 15832 | AY368931 | AY368957 | AY368982 | – | AY371065 |
| *Cynips quercus* | FRf258 FR174 | Madrid, Spain Madrid, Spain | JLNA FR | Galler (W) | *Quercus* (Fg) | 15832 | DQ012638 | DQ012596 | DQ012556 LN811105 | – | – |
| *Dryocosmus quercuspalustris* | ZL017 | Kentucky, USA | EE | Galler (W) | *Quercus* (Fg) | – | DQ012637 | DQ012595 | – | – | – |
| *Neuroterus numismalis* | FRf261 | Madrid, Spain | FR | Part: Leaf galler (W)  Sex: Leaf galler (W) | *Quercus* (Fg) | 15832 | AY368930 | AY368956 | AY368981 | – | – |
| *Plagiotrochus quercusilicis* | FRj005 | Spain | GS | Part: Stem galler (W)  Sex: Leaf Galler (W) | *Quercus* (Fg) | 15832 | AF395178 | AF395154 | AF395162 | – | AF395186 |
| *Trigonaspis mendesi* | FRf259 | Madrid, Spain | JLNA | Part: Leaf galler (W)  Sex: Stem & root galler (W) | *Quercus* (Fg) | – | DQ012658 | DQ012615 | DQ012575 | – | – |
|  |  |  |  |  |  |  |  |  |  |  |  |
| FIGITIDAE |  |  |  |  |  |  |  |  |  |  |  |
| Parnipinae |  |  |  |  |  |  |  |  |  |  |  |
| *Parnips nigripes A* | FRj004 DM222 | Madrid, Spain Madrid, Spain | JLNA JLNA | Parasitoid | *Barbotinia* (Ay) | 15832 | AY368932 – | AY368958 – | AY368983 LN811106 | – LN811125 | AY371066 – |
| *Parnips* *nigripes B* | FR677 | Tarragona, Spain | JLNA | Parasitoid | *Iraella* (Ay) | – | – | DQ012605 | DQ012566 | – | – |
|  |  |  |  |  |  |  |  |  |  |  |  |
| Euceroptrinae |  |  |  |  |  |  |  |  |  |  |  |
| *Euceroptres* *montanus*. | MB425 | California, USA | MB | Parasitoid | (Cy) | 15832 | AY675820 | AY675673 |  | LN811126 | – |
|  |  |  |  |  |  |  |  |  |  |  |  |
| Plectocynipinae |  |  |  |  |  |  |  |  |  |  |  |
| *Plectocynips pilosus* | FR676 | Conguillio, Chile | JLNA | (Parasitoid)^h^ | *Aditrochus* (Chalcidoidea) | 15832 | LN811087 | LN811090 | – | – | – |
| *Plectocynips* sp. | MB264 | Chile | MB | (Parasitoid)^h^ | (*Aditrochus* (Chalcidoidea)) | – | AY675670 | AY675817 | DQ012568 | – | – |
|  |  |  |  |  |  |  |  |  |  |  |  |
| Anacharitinae |  |  |  |  |  |  |  |  |  |  |  |
| *Acanthaegilips* sp. | MB271 | Honduras | UCR | Parasitoid | Hemerobiidae, Chrysopidae | 15832 | AY675679 | AY675826 | – | – | – |
| *Anacharis* sp. | MB274 MB310 | Georgia, USA Ghana | UCR | Parasitoid | Syrphidae | 15832 | AY675674 – | AY675821 – | DQ012543 – | – LN811127 | – |
|  |  |  |  |  |  |  |  |  |  |  |  |
| Charipinae |  |  |  |  |  |  |  |  |  |  |  |
| *Alloxysta* sp. 1 | FRj015 MB344 | Uppsala, Sweden California, USA | FR MB | Parasitoid | Aphidiinae (Br) | 15832 | DQ012618 – | DQ012577 – | DQ012542 – | – LN811128 | – – |
| *Alloxysta* sp. 2 | MB300 | California, USA | MB | Parasitoid | Aphidiinae (Br) | 15832 | AY675705 | AY675849 | – | – | – |
|  |  |  |  |  |  |  |  |  |  |  |  |
| Aspicerinae |  |  |  |  |  |  |  |  |  |  |  |
| *Callaspidia* sp. | MB059 DM235 | Moscow, Russia Tennessee, USA | MB FR | Parasitoid | Syrphidae | 15832 | AY675692 – | AY675837 – | – – | – LN811129 | – – |
| *Paraspicera* sp. | ZL092 | Virginia, USA | DS | Parasitoid | Syrphidae, Chamaemyiidae | 15832 | DQ012648 | DQ012604 | – | – | – |
|  |  |  |  |  |  |  |  |  |  |  |  |
| Eucoilinae |  |  |  |  |  |  |  |  |  |  |  |
| *Agrostocynips* sp. | MB294 MB435 | Mexico Costa Rica | MB MB | Parasitoid | Agromyzidae | 15832 | AY675798 – | AY675927 – | DQ012541 LN811107 | – – | – – |
| *Kleidotoma* sp. | MB273 MB183 | Georgia, USA Moscow, Russia | MB MB | Parasitoid | Sepsidae, Epydridae | 15832 | AY675782 – | AY675914 – | – – | – LN811130 | – – |
| *Trybliographa* sp. 1 | MB163 | Moscow, Russia | MB | Parasitoid | Calliphoridae, Anthomyiidae | 15832 | AY675713 | AY675855 | – | – | – |
| *Trybliographa* sp. 2 | ZL091 | Virginia, USA | DS | Parasitoid | Calliphoridae, Anthomyiidae | 15832 | DQ012659 | DQ012616 | – | – | – |
|  |  |  |  |  |  |  |  |  |  |  |  |
| Figitinae |  |  |  |  |  |  |  |  |  |  |  |
| *Melanips* sp. | MB281 FR473 | California, USA Texas, USA | MB FR | Parasitoid | Chamaemyiidae, Syrphidae | 15832 | AY675690 – | AY675835 – | – LN811108/ LN811109 | – LN811131/ LN811132 | – – |
| *Trischiza* sp. | MB049 | Montana, USA | MB | Parasitoid | Schizophora | 15832 | AY675699 | AY675843 | – | – | – |
|  |  |  |  |  |  |  |  |  |  |  |  |
| LIOPTERIDAE |  |  |  |  |  |  |  |  |  |  |  |
| Mayrellinae |  |  |  |  |  | – |  |  |  |  |  |
| *Paramblynotus virginianus* | FR409 | Virginia, USA | DS | Parasitoid | Unknown | 15832 (*P. zonatus*) | AY368933 | AY368959 | AY368984 | LN811133 | – |
|  |  |  |  |  |  |  |  |  |  |  |  |
| Dallathorellinae |  |  |  |  |  |  |  |  |  |  |  |
| *Dallatorella* sp. | MB341 | New Guinea | MB | Parasitoid | Unknown | 15832 | AY675667 | AY675816 | – | – | – |
|  |  |  |  |  |  |  |  |  |  |  |  |
| IBALIIDAE |  |  |  |  |  |  |  |  |  |  |  |
| Ibaliinae |  |  |  |  |  |  |  |  |  |  |  |
| *Ibalia rufipes* | FRf292 | Nås, Sweden | FR | Parasitoid | *Sirex* (Si) | 15832 | AY368934 | AY368960 | AY368985 LN811110 | LN811134 | – |
| *Ibalia anceps* | FR408 | Virginia, USA | DS | Parasitoid | *Tremex* (Si) | 15832 | DQ012641 | DQ012599 | DQ012560 | – | – |

^a^Repository of vouchers: FR = coll. F. Ronquist, Swedish Museum of Natural History, Stockholm, Sweden; MB = coll. M. Buffington, National Museum of Natural History, Washington, DC, USA; JLNA = coll. J. L. Nieves-Aldrey, Museo Nacional de Ciencias Naturales, Madrid, Spain; ZL = coll. Z. Liu, Department of Biological Sciences, Eastern Illinois University, Charleston, IL, USA.

^b^AS = A. Stojanov, BM = B. Massa, DS = D. Smith, EE = E. A. Eliason, EQ = ZL & E. Quinter, FR = F. Ronquist, GS = G. Stone, JLNA = J.-L. Nieves-Aldrey, JPV = J. Pujade-Villar, JT = J. F. Tooker, OP = O. Plantard, SN = Stefan Neser, SO = Sergio Ovruski, SvN = S. van Noort, UCR = University of California, Riverside, ZL = Z. Liu.

^c^Gall inducers are classified as being gallers on either woody (W) or herbaceous (H) host plants. Life history in parenthesis is inferred from the life history of related lineages.

^d^Host genus or family of the exemplar species. The host of inquilines is the gall inducer they are associated with. Abbreviations of higher taxa: An= Anacardiaceae, As= Asteraceae, Ay = Aylacini (Cynipidae), Br = Braconidae, Ce = Cecidosidae (Lepidoptera), Cy = Cynipini (Cynipidae), Di = Diplolepidini (Cynipidae), Fb = Fabaceae, Fg = Fagaceae, La = Lamiaceae, Pa = Papaveraceae, Ro = Rosaceae, Sa = Salicaceae, Si = Siricidae.

^e^See Appendix S1 for a list of characters. The column only refers to the morphological characters; life-history (ecology) characters were scored for all taxa (same TreeBase reference number).

^f^*Andricus mayri* (Wachtl, 1879), not *Andricus mayri* (Kieffer, 1897) or *Andricus mayri* (Stefani, 1889).

^g^Reared from galls on *Nothofagus*, which are induced by species of *Aditrochus* (Chalcidoidea: Pteromalidae: Ormocerinae). The biology of *Paraulax*, *Cecinothofagus* and *Plectocynips* is not known with certainy, but they are likely to be either phytophagous inquilines (*Cecinothofagus*) or possibly parasitoids.
